# Amine-Linked Covalent Organic Frameworks as a Platform for Postsynthetic Structure Interconversion and Pore-Wall Modification

**DOI:** 10.1021/jacs.0c12249

**Published:** 2021-02-24

**Authors:** Lars Grunenberg, Gökcen Savasci, Maxwell W. Terban, Viola Duppel, Igor Moudrakovski, Martin Etter, Robert E. Dinnebier, Christian Ochsenfeld, Bettina V. Lotsch

**Affiliations:** † Max Planck Institute for Solid State Research, Heisenbergstrasse 1, 70569 Stuttgart, Germany; ‡ Department of Chemistry, Ludwig-Maximilians-Universität (LMU), Butenandtstrasse 5-13, 81377 Munich, Germany; § Deutsches Elektronen-Synchrotron (DESY), Notkestrasse 85, Hamburg 22607, Germany; ∥ E-conversion, Lichtenbergstrasse 4a, 85748 Garching, Germany and Center for NanoScience, Schellingstrasse 4, 80799 Munich, Germany

## Abstract

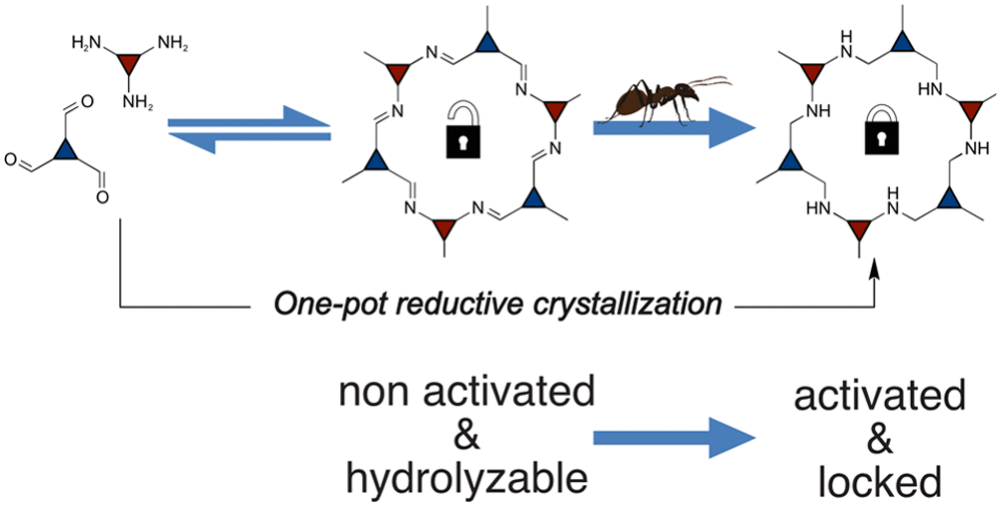

Covalent organic frameworks have emerged as a powerful synthetic platform for installing and interconverting dedicated molecular functions on a crystalline polymeric backbone with atomic precision. Here, we present a novel strategy to directly access amine-linked covalent organic frameworks, which serve as a scaffold enabling pore-wall modification and linkage-interconversion by new synthetic methods based on Leuckart–Wallach reduction with formic acid and ammonium formate. Frameworks connected entirely by secondary amine linkages, mixed amine/imine bonds, and partially formylated amine linkages are obtained in a single step from imine-linked frameworks or directly from corresponding linkers in a one-pot crystallization-reduction approach. The new, 2D amine-linked covalent organic frameworks, rPI-3-COF, rTTI-COF, and rPy1P-COF, are obtained with high crystallinity and large surface areas. Secondary amines, installed as reactive sites on the pore wall, enable further postsynthetic functionalization to access tailored covalent organic frameworks, with increased hydrolytic stability, as potential heterogeneous catalysts.

## Introduction

In recent years, covalent organic frameworks (COFs) have emerged as a versatile class of crystalline porous polymers, which have been pushing the frontiers of single-site heterogeneous catalysis ever since. The unique combination of ordered and tunable pore structures, with high surface areas and versatile (opto-)electronic properties, offers great opportunities beyond gas storage and separation, including sensing, electrochemical energy storage, optoelectronics, and heterogeneous (photo)catalysis.^1−4^

Imine-linked COFs constitute the most widely studied subclass of COFs owing to their broad synthetic scope and facile building block synthesis. The dichotomy of dynamic covalent chemistry in COF synthesis implies that, while reversible bond formation is critical for crystallization, the reversibility of imine bond formation also causes its limited stability against hydrolysis. To address this issue, several postsynthetic locking strategies have been developed in the past, e.g., converting labile imine-linked COFs into stable benzothiazole-,^5,6^ amide-,^7^ or quinoline-linked frameworks.^4,8−13^ Although these methods significantly increase the material’s hydrolytic stability, most do not activate but rather deactivate potential reactivity of the linkages for further pore-wall modification. To achieve the latter, reactive centers have to be installed into the linker moieties, which are often incompatible with synthesis conditions. This incompatibility requires an additional pore-wall activation step, e.g., the reduction of nitro groups to amines^14^ or the deprotection of ethers to alcohols.^15^ In essence, a typical synthetic route would consist of at least four sequential steps, including (a) framework crystallization, (b) linkage transformation, (c) pore-wall activation, and (d) pore-wall functionalization, to obtain both stable and decorated frameworks. Being faced with varying conversion yields and a loss of material between each step, innovative synthetic methods condensing these transformations into fewer steps, or even a single synthetic step, are highly desirable.

As a solution to the challenges discussed, we here demonstrate amine-linked covalent organic frameworks as a powerful platform for facile pore-wall modification and linkage interconversion enabled by new synthetic methods based on Leuckart–Wallach^16,17^ reduction with formic acid and ammonium formate. By fine-tuning reaction conditions, frameworks connected entirely by secondary amine linkages, mixed amine/imine bonds, and partially formylated amine-linkages are accessible in a single step from imine-linked frameworks or directly from the corresponding linkers in a one-pot crystallization-reduction approach. We thus present a novel strategy enabling direct access to amine-linked covalent organic frameworks. In addition, we reveal correlations between topologically equivalent disordered and crystalline frameworks, which are not accessible by typical X-ray powder diffraction (XRPD) analysis, using pair distribution function (PDF) analysis, solid-state nuclear magnetic resonance spectroscopy (ssNMR), and quantum-chemical calculations. These findings enable us to identify unique pH-dependent amorphization pathways and hence expand our fundamental understanding of amine-linked covalent organic frameworks as an important yet underexplored class of heterogeneous catalysts.

## Results

### Previous Strategies and Drawbacks

During our studies, we found that a reduction of imine-linkages would both increase the hydrolytic stability of the framework and introduce secondary amine-linkages as reactive centers for further functionalization of the pore wall. This transformation, familiar from small organic molecules as well as molecular cages, is usually achieved using borohydride-based reducing agents, such as sodium borohydride or sodium cyanoborohydride.^18−20^ Borohydride-based reduction has successfully been used for robust and rigid 3D systems, while 2D frameworks have only been obtained with diminished crystallinity and low surface areas at best.^11,15,21^ While highly reactive, reactions with sodium borohydride, in particular, suffer from limited selectivity and low functional group tolerance.^5,22^ With these shortcomings in mind, we sought an alternative, mild reduction procedure affording crystalline and porous amine-linked covalent organic frameworks. To this end, we identified the Leuckart–Wallach reduction with formic acid, reported for small organic molecules by Leuckart in 1885 and further developed by Wallach, as a suitable reduction strategy.^16,17^

### Synthesis of Amine-Linked COFs

**Figure 1:**
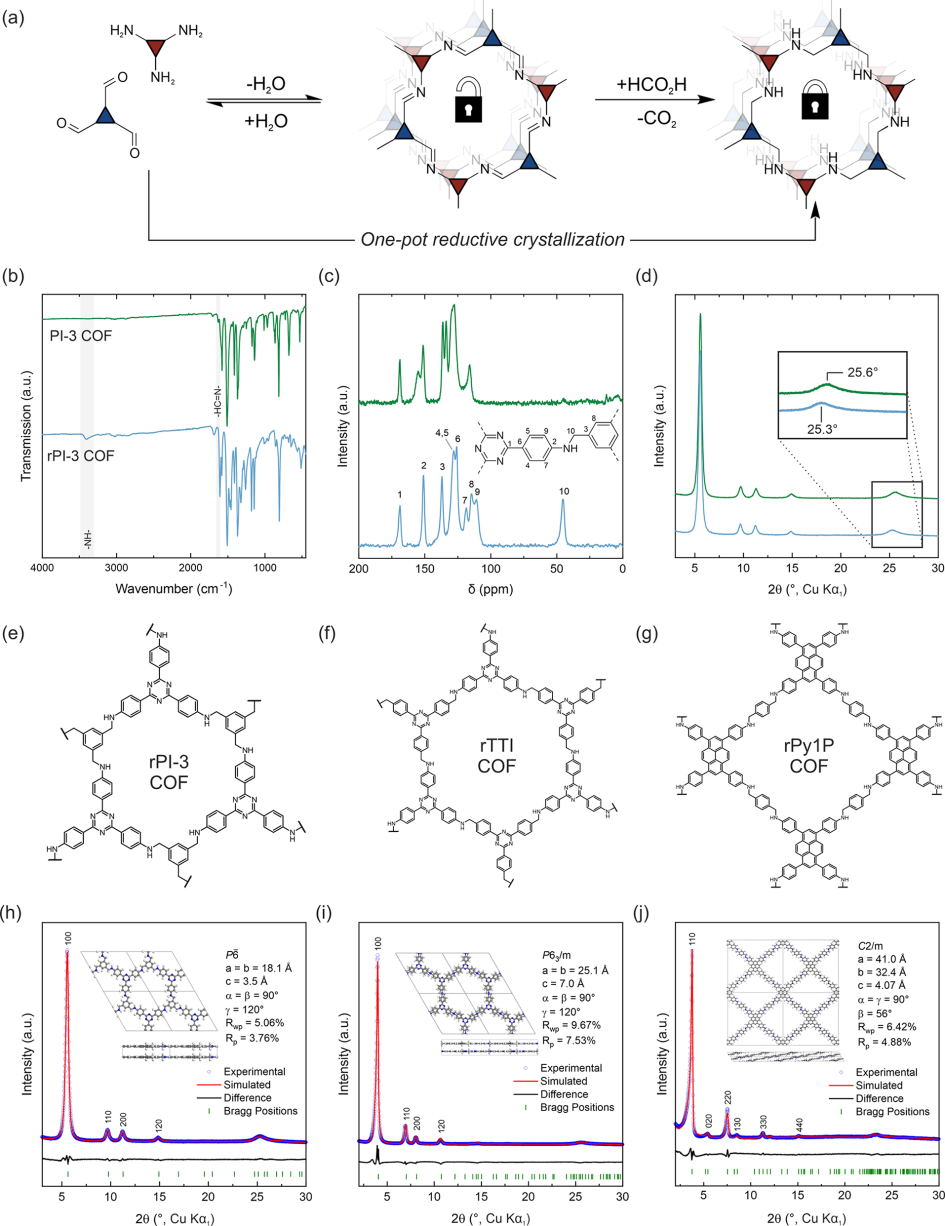
(a) Synthesis of amine-linked covalent organic frameworks. (b) FT-IR spectra, (c) ^13^C CP-MAS ssNMR spectra, and (d) XRPD pattern comparison of PI-3-COF (green) and rPI-3-COF (blue). (e and f) Chemical structure of a single pore of (e) rPI-3, (f) rTTI, and (g) rPy1P-COF. (h–j) Rietveld refinements for (h) rPI-3, (i) rTTI, and (j) rPy1P-COF.

As a model system, we first synthesized the imine-linked PI-3-COF from 1,3,5-triformyl benzene (TFB) and 4,4′,4″-(1,3,5-triazine-2,4,6-triyl)trianiline (TTA) under solvothermal conditions in a 2:1 mesitylene/1,4-dioxane mixture with aqueous 6 M AcOH at 120 °C for 72 h, according to a modified literature procedure.^23^ Upon reacting the imine-linked PI-3 framework in a sequential step with 19 equiv of formic acid in the same solvent, a new vibration appeared at 3405 cm^–1^ as probed by Fourier transform infrared spectroscopy (FT-IR), attributed to a secondary amine (v_N—H_) stretching mode. The intensity of the imine vibration (v_C=N_) at 1630 cm^–1^ gradually decreased over prolonged reaction time at 120 °C (Figures S1 and S2). Extensive screening for the highest relative intensities of the secondary amine vibrations in the IR spectrum yielded optimal synthetic conditions at 21 equiv of formic acid in a 2:1 mesitylene/1,4-dioxane mixture and a reaction time of 24 h at 120 °C. Under these conditions, the samples did not show any residual imine stretch vibration (v_C=N_), hinting at the complete transformation of the parent PI-3-COF structure (Figure 1b). ^13^C cross-polarization magic angle spinning (CP-MAS) solid-state NMR (ssNMR) spectroscopy similarly shows the disappearance of the characteristic imine carbon signal at 155.3 ppm, while a new aliphatic carbon signal at 45.4 ppm is visible for the reduced PI-3-COF (rPI-3-COF). Besides that, new signals at 119 and 114 ppm become visible for rPI-3-COF, assigned to the aromatic carbons next to the amine bond (Figure 1c). ^15^N ssNMR of rPI-3-COF shows distinct signals at −313.3 ppm for the secondary amine nitrogen and at −141.4 ppm for the triazine (Figure S30). The absence of the imine nitrogen at −59.0 ppm further suggests a quantitative reduction of imine into amine linkages in rPI-3-COF. The measured ssNMR chemical shifts are in good agreement with values obtained by quantum-chemical calculations of representative molecular and single-pore models (Table S5).

Structural analysis of rPI-3-COF via XRPD reveals high crystallinity (Table S3), represented by four narrow reflections at 2θ = 5.6°, 9.7°, 11.2°, and 14.9°, indexed as 100, 110, 200, and 210 reflections (space group *P*6̅), and a broad stacking reflection at 2θ = 25.3°. Compared to its parent imine structure (PI-3-COF), the apparent hexagonal symmetry and crystallinity are retained, while a significant shift of the broad stacking reflection at 2θ = 25.6° (PI-3-COF) toward smaller angles appears. Rietveld^24^ refinement gives a larger in-plane unit cell parameter of *a* = 18.090(7) Å and an increased stacking distance of *c* = 3.5425(12) Å in rPI-3-COF (*a* = 18.034(7) Å and *c* = 3.5058(12) Å for PI-3-COF) (Table S1). While the cell parameter *a* is affected by increased C—N (149 pm) vs C=N (127 pm)^25^ bond lengths, the stacking distance (*c* parameter) is also influenced by both enhanced steric repulsion of the benzylic (CH_2_) protons of adjacent layers and higher flexibility of the secondary amine bond in rPI-3-COF. Notably, sorption isotherms reveal the complete retention of porosity and pore-size distributions (Figure S54, S64) of the materials with Brunauer–Emmett–Teller (BET) surface areas of 1395 m^2^g^–1^ for rPI-3-COF (Figure S74) and 1404 m^2^g^–1^ for PI-3-COF (Figure S71), even exceeding those previously published for PI-3-COF (∼1000 m^2^g^–1^).^23^ It must be noted, however, that the porosity of rPI-3-COF is strongly influenced by the drying procedure: Simple vacuum-drying from dichloromethane resulted in a reduced BET surface area of 966 m^2^g^–1^ (Figure S77), while solvent exchange to methanol (Soxhlet extractor) and subsequent activation with supercritical CO_2_ (scCO_2_) gave the best results for rPI-3-COF with 1395 m^2^g^–1^ (Figures S69 and S74). While this effect was not observed for the rigid imine PI-3 framework, an increased flexibility in rPI-3-COF and a modulated pore-wall polarity upon reduction are expected to enhance solvent interactions and capillary effects, potentially intensifying drying-induced disorder and pore collapse.^26,27^ Scanning electron microscopy (SEM) and transmission electron microscopy (TEM) images reveal intergrown, coral-shaped particle morphologies with sizes between 600 to 1000 nm, decorated with 200 nm long and 60 nm wide stings, for both imine-linked and reduced PI-3-COF (Figures S82 and S85). The similarity of the morphology before and after reduction renders intermediate recrystallization processes unlikely to be at play. TEM images show uniformly distributed crystallinity and extended porous channels of hexagonal symmetry in the materials, which are consistent with the structural model derived from XRPD data and Rietveld refinement (Figures S91 and S96).

To demonstrate the general applicability of this protocol, we applied it to two additional imine COFs with larger pores, different linker compositions, and pore geometry. Both TTI-COF and Py1P-COF were successfully reduced to their new amine-linked derivatives rTTI-COF and rPy1P-COF with high crystallinity as evident from sharp reflections at 2θ = 4.0° (100), 6.9° (110), 8.1° (200), and 25.6° (stacking) for rTTI and 2θ = 3.7° (110), 5.4° (020), 7.5° (220), 8.5° (130), 11.3° (330), and 23.2° (stacking) for rPy1P-COF, respectively. During our screenings to find the optimum reduction conditions for rPy1P-COF, we noticed palladium contamination in both the building blocks and the framework, introduced by palladium-based cross-coupling reactions during linker synthesis. The TEM images of an initial sample of Py1P-COF (Pd contaminated) show unevenly distributed palladium nanoparticles in the material (Figures S94 and S95).^28^ While this contaminant did not affect the crystallization step of Py1P-COF, the metal particles “overcatalyzed” the reduction with formic acid, causing a partial digestion of the framework. To avoid this contamination, the 4,4′,4″,4″′-(pyrene-1,3,6,8-tetrayl)tetraaniline linker was further purified on a metal scavenger (Biotage Isolute Si-TMT). A purified imine-linked Py1P-COF was then crystallized and reduced with formic acid to form rPy1P-COF without any noticeable decomposition. The SEM and TEM images of rTTI and rPy1P-COF thus show full retention of the porous, crystalline features of their parent frameworks (Figures S84, S89, S93, and S100).

Similar to the rPI-3-COF model system, new N–H vibrations at 3407 (rTTI) or 3398 cm^–1^ (rPy1P) in the FT-IR spectra and secondary amine nitrogen signals at −314.9 (rTTI) and −317.6 ppm (rPy1P) in the ^15^N-ssNMR spectra prove the conversion into amine-linked frameworks (Figures S3, S4, S35, and S38). Although both samples show good porosity (1419 m^2^g^–1^ rTTI, 1042 m^2^g^–1^ rPy1P), the BET surface area of rPy1P is reduced, compared to 1883 m^2^g^–1^ in Py1P-COF (Figures S72, S73, S75, and S76). By comparing linker geometries in small-pore hexagonal rPI-3, large-pore hexagonal rTTI, and square-net rPy1P-COF, those frameworks consisting of more rigid tritopic + tritopic [3 + 3] linker combinations (rPI-3 and rTTI-COF) show full retention of the BET surface area, while the tetratopic + bitopic [4 + 2] linker combination in rPy1P-COF was obtained with a slightly reduced surface area. This is in line with the expected additional flexibility around the molecular axis of the bitopic terepthalaldehyde linker, which facilitates local distortions in the network, causing reduced accessibility of the pores and thus a smaller surface area in rPy1P-COF.

Upon reduction, the in-plane cell parameters of larger-pore square-net rPy1P-COF were barely influenced, with C–N bond lengths contributing only a small percentage to the overall pore-to-pore distance. On the contrary, the increased stacking distance caused an expansion of the unit cell from *c* = 3.818(4) Å to *c* = 4.069(9) Å, similar to the small-pore rPI-3-COF. At first sight, these trends cannot be found for the rTTI framework in direct comparison to TTI-COF. Apparent symmetry^29^ changes (peak splitting) in the XRPD pattern, however, show that upon reduction the stacking behavior of TTI-COF changes from antiparallel slip-stacked TTI (*P*1) to more eclipsed-like stacking in rTTI with an average crystallographic symmetry of *P*6_3_/*m* (Figures S9 and S13 and Tables S1 and S2). Similar symmetry correlations have been described for the TTI-COF system by Haase et al. in comparison to its randomly stacked TTI (rsTTI) framework, resulting in both in-plane and interlayer contractions.^29,30^ This trend is also observed with rTTI-COF: The increase in symmetry is accompanied by a pore contraction (*a* = 25.786(12) Å (TTI) vs *a* = 25.147(9) Å (rTTI)), caused by changed bond angles of antiparallel amine bonds, resulting in overall reduced intralayer cell parameters for rTTI. Furthermore, enhanced interlayer interactions in the eclipsed-stacked rTTI-COF compensate repulsive steric effects of benzylic protons, and thus, stacking distances decrease from 3.578(9) Å in TTI to 3.504(2) Å in the reduced framework (Tables S1 and S2).

### One-Pot Procedure: Reductive Crystallization

When comparing the conditions needed for the synthesis of the imine framework and the following reduction, acids and the same solvent mixture are used in both cases and only the amount and type of acid changes. Thus, we expected that formic acid could act as a catalyst for both the formation and reduction of the framework, condensing the individual steps into a single one-pot crystallization-reduction approach.

Indeed, with 21 equiv of formic acid in a 2:1 mixture of mesitylene/1,4-dioxane at 120 °C for 72 h, a crystalline sample of rPI3-COF was obtained directly from its corresponding aldehyde and amine building blocks (Figure S15). Compared to its two-step analogue, it was obtained in a different, spherical morphology (Figure S87). As visible from broadened signals in the ^13^C ssNMR and FT-IR spectra (Figures S7 and S49), this sample is structurally less well-defined with a major impact on the resulting porosity (BET area of 174 m^2^g^–1^, Figure S79), which may also be reduced by trapped oligomers in the pores of the framework, besides structural defects. During our studies, we noticed a significant impact of the reaction temperature on the obtained product. While formic acid catalyzes the imine condensation both at high (120 °C) and already at low (60 °C) temperatures, the subsequent reduction is fast only at an elevated temperature (Figure S16). As such, the one-pot protocol can be used to *thermally* switch between the reversible synthesis of an imine-linked COF at a low temperature or the irreversible “locking” of the framework structure by simultaneous reduction to the amine-linked COF using otherwise identical reaction conditions. We expect this unique property to be key for the adaptation to other covalent organic frameworks. Besides this “thermo-switchability”, it highlights formic acid as a versatile yet underexplored catalyst for the synthesis of imine-linked COFs at a reduced temperature.

### Reductive Formylation: Combined Reduction and Protection

Solid ammonium formate as a green, less toxic, and less corrosive alternative to formic acid was also effective for the reduction of imine bonds under solvent-free conditions. Reacting a salt-melt of ammonium formate and PI-3-COF for 3 h at 170 °C in a closed vessel afforded a product with broadened secondary amine vibrations at v_N—H_ = 3370 cm^–1^ and carbonyl stretching modes at v_C=O_ = 1669 cm^–1^ in the FT-IR spectrum (Figure S6). An additional signal at 162.8 ppm in the ^13^C ssNMR spectrum, referring to an N-formyl-carbon (Figures S43 and S46), shows that, besides the reduction, a subsequent N-formylation resulted in a partially formylated, reduced PI-3 framework (pfrPI-3-COF). A comparison of FT-IR and ssNMR spectra excludes a degradation of the chemical connectivity. The XRPD pattern shows a substantial reduction in the long-range order reminiscent of an amorphous solid, although a small feature corresponding to the 100 peak further suggests that the intralayer connectivity is maintained (Figure S17). When reacting pfrPI-3-COF with 2,3-dichloro-5,6-dicyano-1,4-benzoquinone (DDQ) in dichloromethane, the secondary amine linkages are oxidized back to the imine linkages, affording reoxidized, partially formylated reduced PI-3-COF (opfrPI-3-COF).^15^ Remarkably, after this treatment, sharp signals in the XRPD pattern similar to the parent PI-3 framework become visible (Figures S11 and S17). The feasibility of this amorphous-to-crystalline conversion suggests a significant topological and structural similarity of the reduced, amorphous COF to the crystalline compound and led us to further investigate the correlations between the crystalline and non-crystalline amine-linked frameworks.

### Crystalline vs Disordered

During our screenings to find optimal reduction conditions for the imine-linked frameworks, we noticed that a large excess of formic acid can decrease the crystallinity of the product, suggesting a profound role of protonation on the layer structure. Using 59.5 equiv of formic acid with PI-3-COF under the same conditions as above leads to a practically X-ray amorphous structure with slightly broadened but otherwise essentially identical signals as rPI-3-COF in the FT-IR and ssNMR spectra (Figures S5 and S48). SEM and TEM did not show any morphological changes of the particles (Figures S86 and S97). Stability tests of rPI-3-COF, e.g., under acidic conditions, show similar effects on the reflections in the XRPD patterns. In contrast to the imine-linked PI-3-COF, the amine-linked rPI-3-COF does not show any hydrolytic decomposition, though sharp reflections in the XRPD pattern broaden or disappear completely upon treatment with excess acid—illustrating local structural changes and distortion of the stacked 2D layers (Figures S101 and S102).

**Figure 2:**
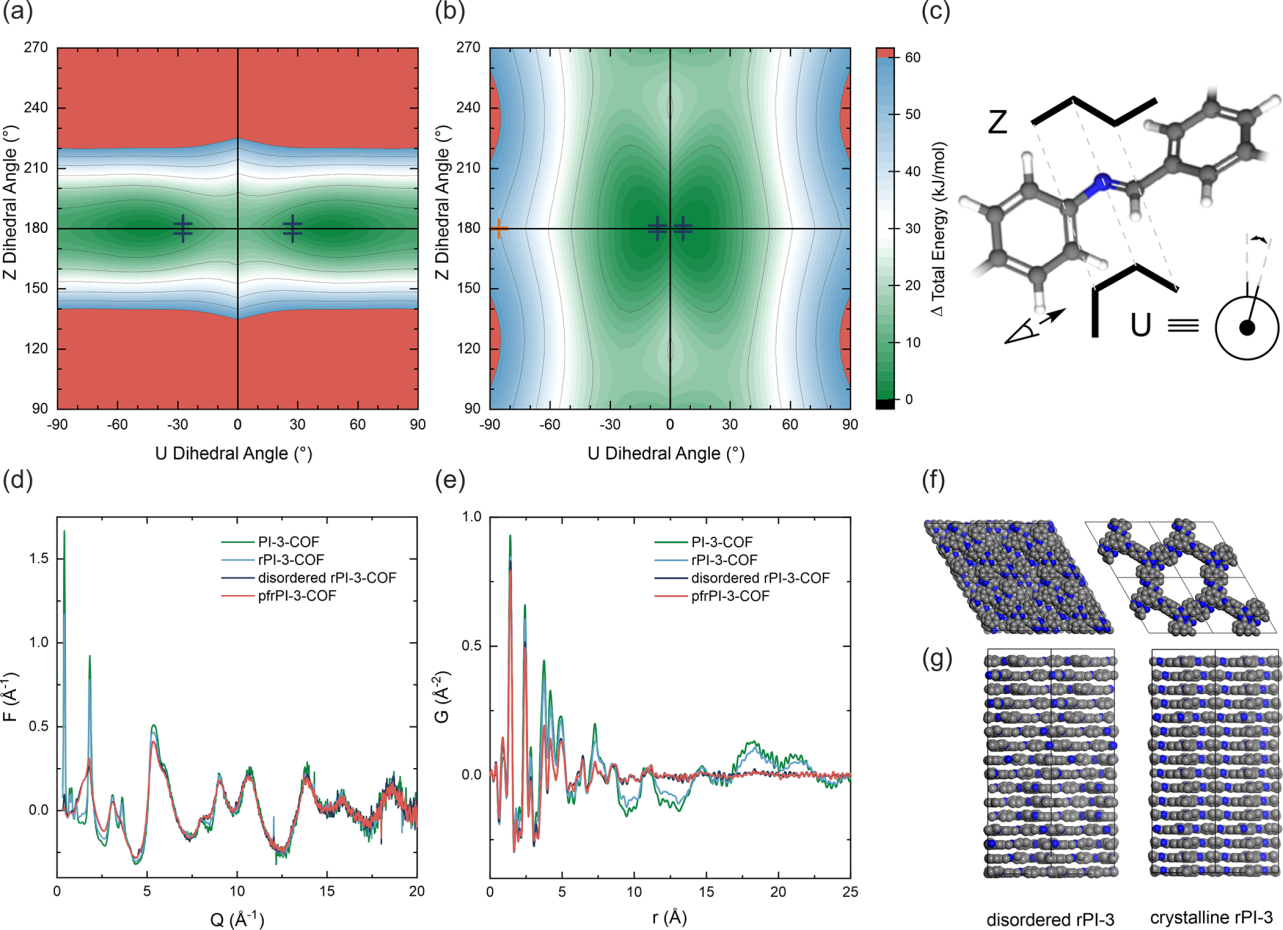
(a) Energy surface plot for different values of dihedral angles U and Z in the molecular model PI-3 M. The blue crosses highlight the lowest energy conformation for the single-pore model PI-3 SP. (b) Energy surface plot for the molecular model rPI-3 M. The blue crosses highlight the lowest energy conformation for the single-pore model rPI-3 SP. As a comparison, the conformation of a protonated molecular model H+rPI-3 M is shown on the surface (red cross). Notably, in this simple molecular model, the effect of the anion and charge repulsion, occurring in the layered real structure, are neglected. (c) Dihedral angles U and Z shown in a section of PI-3 M. (d) Reduced total scattering patterns and (e) pair distribution functions for crystalline and disordered materials are overlaid. (f and g) Sixteen-layer structure models derived from PDF analysis for disordered rPI-3-COF (left) and rPI-3-COF (right) considering random translational disorder in 1–10 directions are shown.

To elucidate conformational changes in the structure of PI-3-COF upon reduction, quantum-chemical calculations on the PBE0-D3/def2-TZVP level of theory were performed to obtain optimized structures for model compounds.^31−34^ The surface plot for combined rotations around dihedral angles U and Z in molecular models PI-3 M and rPI-3 M (Figures S103–S105) shows an increased flexibility for the amine-linked molecular model, apparent from a broad range of low energy conformations (Figure 2a–c). Optimized single-pore models PI-3 SP and rPI-3 SP (Figures S106 and S107) depict discrete points on the surface close to the lowest energy conformations of their molecular models with dihedral angles (U) of 27.4° (PI-3 SP) and 6.46° (rPI-3 SP). Although interlayer steric repulsion in the amine framework is increased due to additional benzylic protons (C-10, Figure 1c) that align perpendicular to the 2D surface, reduced 1,4-repulsion between protons at C-9 and C-10 causes a flattening of the dihedral angle U, albeit combined with increased flexibility of the structure. These results corroborate the cell parameter changes upon reduction as observed by Rietveld analysis of the crystalline rPI-3-COF. To elucidate possible amorphization pathways, resulting in a disordered structure of rPI-3 if synthesized with an excess of formic acid, additional protonation must be considered. When protonated at the amine nitrogen, the dihedral angle U undergoes a significant widening to 86.6° in the molecular model H+rPI-3 M (Figure S105)—a rotation associated with an energy barrier of approximately 50 kJ/mol in the molecular model rPI-3 M (without protonation). Considering conformational restrictions in the layer geometry of the rPI-3-COF, less pronounced but still substantial conformational changes, combined with interlayer charge repulsion, are expected, leading to a significant disruption of the periodicity of the stacked layers as a function of the pH. This effect is further supported by a vast signal broadening at 119 ppm (C-7) in the ^13^C ssNMR spectrum with increasing disorder in rPI-3-COF (Figure S48), whereas in the actual, well-ordered rPI-3-COF structure, a narrow statistical distribution of dihedral angles indicates a preferred conformation and thus a fairly sharp signal for this carbon (C-7). A protonation-dependent broad distribution in the disordered rPI-3-COF causes this signal to broaden and, ultimately, to vanish, however without disrupting the overall connectivity of the layer.

To determine the local and intermediate length-scale structure modifications due to conformation-induced disordering, we performed pair distribution function (PDF) analysis on X-ray total scattering synchrotron data. Notably, a high similarity in the reduced total scattering patterns (Figure 2d) from ∼5–20 Å^–1^ and peak positions up to approximately 7 Å in the PDFs of all samples evidence intact, imine or amine bonded layer connectivity in the disordered state. The PDFs of PI-3-COF and rPI-3-COF (Figure 2e) show distinct medium- and long-range ordered structuring, consisting of two primary oscillations due to the ordering of the stacked layers (higher frequency) and porous channels (lower frequency). The structural correlations are more strongly damped for disordered rPI-3-COF and pfrPI-3-COF, becoming relatively flat around 12 Å. This indicates that the spatial relationships of atoms in stacked layers and across porous channels are largely reduced, although, as seen in the diffraction patterns, there are still weakly correlated motifs over at least a few layers or pore distances (Figure 2e). Distinct differences could be visualized between crystalline and disordered structures by the refinement of a 16-layer structure model to the PDFs for rPI-3-COF and disordered rPI-3-COF PDFs, with random translations allowed in a single direction (Figure 2f,g). For the disordered sample, much larger translations were required to damp out the interlayer and ordered pore channel structure signals. It must be noted that these models may overpredict layer translations due to undersampling the number of layers. Furthermore, the interlayer correlations could also be damped by larger and random torsions of the amine or phenyl moieties, as shown in quantum-chemical single-pore models. Average stacking offsets were estimated by refining models to the PDFs in the range of neighboring layers, i.e., *r* < 6 Å, using PDFgui.^29,35^ The values obtained are 1.0 (PI-3-COF), 1.2 (rPI-3-COF), 3.3 (disordered rPI-3-COF), and 3.3 Å (pfrPI-3-COF). As visible from the disordered model, random layer translations drastically reduce the pore accessibility and thus help to explain reduced BET surface areas for the disordered models.

### Hybrid Materials and Functionalization

**Figure 3:**
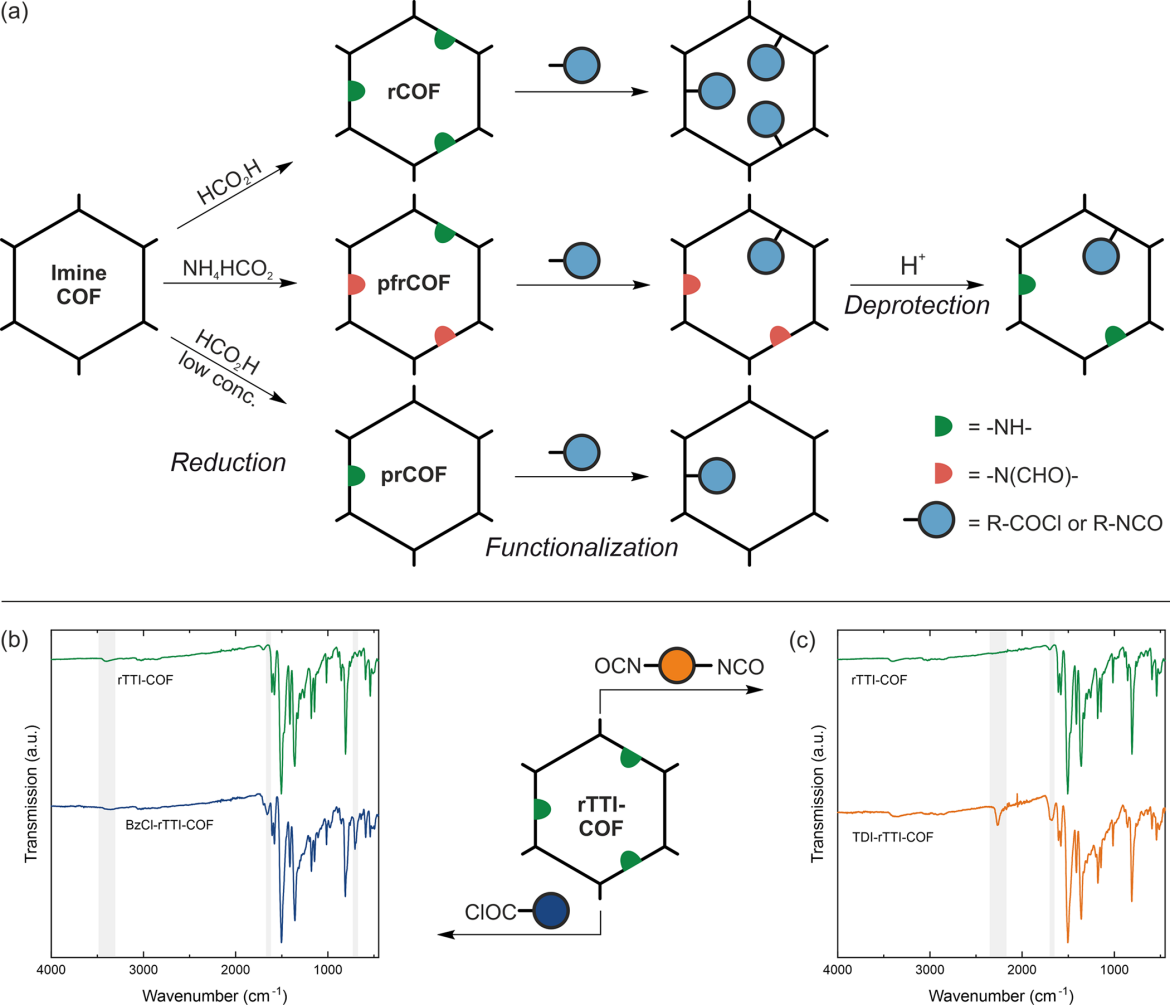
(a) Reaction sequence for postsynthetic functionalization of amine-linked covalent organic frameworks. Frameworks entirely connected by secondary amine linkages (rCOF) or hybrid materials with mixed amine/imine bonds (prCOF) and partially formylated amine-linkages (pfrCOF) are accessible in a single step from imine COFs. As experimentally shown with pfrPI-3-COF (middle), *N*-formyl groups in pfrCOFs can be deprotected under acidic conditions. Released secondary amine linkages may allow two-step functionalization to afford bifunctionalized frameworks. The amine/*N*-formyl amine ratio is arbitrary. (b and c) FT-IR spectra of rTTI-COF samples functionalized with (b) benzoyl chloride (BzCl) and (c) toluenediisocyanate (TDI) are shown and compared to rTTI-COF. Gray areas in (b) highlight reduced N—H, emerging C=O, and characteristic C=C vibrations in BzCl-rTTI-COF. For TDI-rTTI-COF, vibrations of dangling -NCO and emerging C=O vibrations are highlighted (gray).

Besides frameworks containing only amine or imine linkages, hybrid materials with varying imine/amine linkage content can also be obtained with our method by adjusting reaction time and the amount of formic acid (Figure 3a and Figure S1). As an example, partially reduced Py1P-COF (prPy1P-COF) was synthesized, showing distinct signals at 149.0 (imine) and 146.4 ppm (amine) in the ^13^C ssNMR spectrum for the aromatic carbon next to the nitrogen (approximately 42% amine sites, Figure S40). Another example, already introduced, is partially formylated reduced PI-3-COF (pfrPI-3-COF) obtained from PI-3-COF via salt-melt reduction with ammonium formate. The presence of *N*-formyl groups opens up further avenues for additional framework functionalization. For instance, partially functionalized frameworks may be generated by reacting the partially formylated framework with an electrophile, since formyl groups act as a protecting group for secondary amine sites. Partial functionalization can avoid reduced pore accessibility and diffusion limitations, which is critical, for example, in catalysis.^36^ In a more complex case, bifunctionalized frameworks may be synthesized in a subsequent step, after exposing previously protected amine sites. The deprotection of *N*-formyl groups in pfrPI-3-COF was achieved under acidic conditions (aqueous 1 M HCl, 120 °C, 20 min), affording rPI-3-COF as evident from a vanishing formyl signal at 162.8 ppm in the ^13^C ssNMR spectrum (Figure S46), while acid chlorides or isocyanates have proven as strong and effective electrophiles to derivatize secondary amines in rTTI-COF (Figure 3b,c).

## Discussion

**Figure 4:**
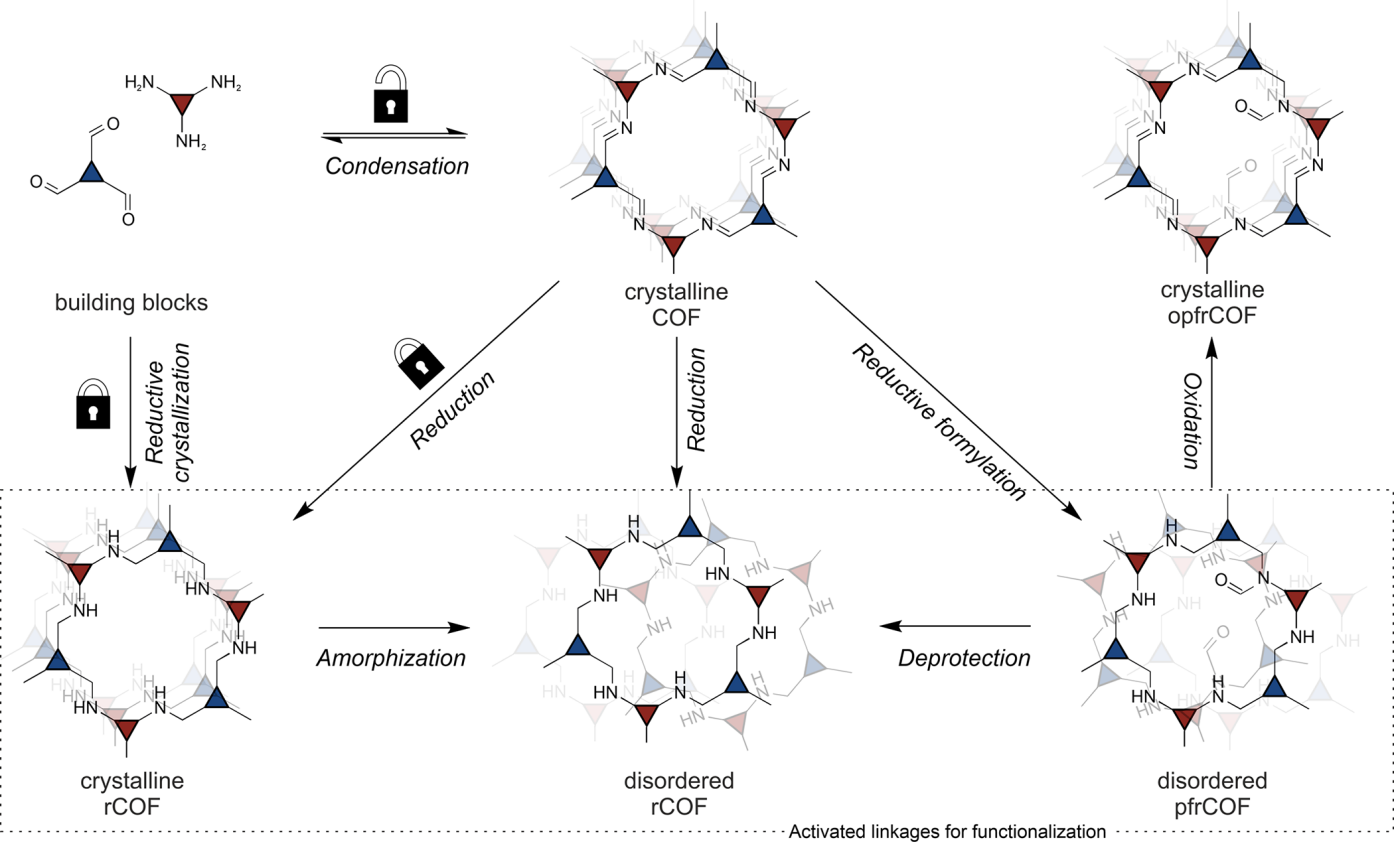
Pathways leading to a set of amine-linked covalent organic frameworks as demonstrated with the PI-3 COF system.

In summary, amine-linked frameworks were introduced as a hydrolytically stable and tailorable system for further postsynthetic modification, which can be accessed from imine-linked frameworks or directly from their corresponding amine and aldehyde building blocks (Figure 4). In contrast to many earlier locking strategies, generating amide-, benzoxazole-, or benzothiazole-linked frameworks, our approach locks and simultaneously activates the connectivity of the framework for further functionalization.^5,7,11,37^ The introduced reduction methods using either formic acid or ammonium formate give access to a range of fully amine-linked or intermediate amine-/imine-linked crystalline frameworks with large surface areas or topologically identical, disordered analogues with reduced pore-accessibility. Importantly, the degree of amine functionalization can be rationally controlled by adjusting the amount of acid and the reaction time. For the first time, we demonstrate amine-linked frameworks as a modular platform enabling the facile interconversion of chemically and structurally distinct frameworks, including reduction–reoxidation cycles and crystalline-to-disordered and disordered-to-crystalline conversions. Finally, we show that the obtained amine linkages readily react with electrophiles such as acid chlorides and isocyanates, opening new avenues to the facile postsynthetic functionalization of COFs at the linkage site with a built-in protection–deprotection strategy and without the need for additional building block engineering. In essence, the demonstrated methods enable hitherto undiscovered functionalization strategies that are widely applicable to all imine-linked covalent organic frameworks, the largest family of COFs to date.

## Supporting Information Available

Discussions of methods and equipment used, detailed synthetic procedures, FT-IR spectra, calculated IR modes, XRPD patterns, ^1^H, ^13^C, ^15^N and ^1^H/^13^C-HETCOR ssNMR spectra, N_2_ sorption isotherms, pore-size distributions, BET plots, SEM and TEM images, SEM and TEM-EDX spectra, quantum-chemically optimized structures, calculated NMR chemical shifts, reduced total scattering patterns, PDF analyis, structure refinements and tables of cell parameters of Rietveld refined imine- and amine-linked COFs (PDF)
