# Supplementary material for: Amine-Linked Covalent Organic Frameworks as a Platform for Postsynthetic Structure Interconversion and Pore-Wall Modification
Source: J Am Chem Soc. 2021 Feb 24;143(9):3430–8. doi: 10.1021/jacs.0c12249 (PMC7953377; doi:10.1021/jacs.0c12249)
Supplement: Supplementary file 1 — ja0c12249_si_001.pdf [file ja0c12249_si_001.pdf]

Supporting Information:

Amine-Linked Covalent Organic Frameworks as a Platform for Postsynthetic Structure Interconversion and Pore-Wall Modification

Lars Grunenberg,[†,‡] Gökcen Savasci,[†,‡,ǁ] Maxwell W. Terban,[†] Viola Duppel,[†] Igor Moudrakovski,[†] Martin Etter,[§] Robert E. Dinnebier,[†] Christian Ochsenfeld,[‡,†,ǁ] and Bettina V. Lotsch.[*,†,‡,ǁ]

[†] Max Planck Institute for Solid State Research, Heisenbergstrasse 1, 70569 Stuttgart, Germany

[‡] Department of Chemistry, Ludwig-Maximilians-Universität (LMU), Butenandtstrasse 5-13, 81377 Munich, Germany

[§] Deutsches Elektronen-Synchrotron (DESY), Noltkestrasse 85, Hamburg 22607, Germany

[ǁ] E-conversion, Lichtenbergstrasse 4a, 85748 Garching, Germany and Center for NanoScience, Schellingstrasse 4, 80799 Munich, Germany

*Email: b.lotsch@fkf.mpg.de

Table of Contents

Table of Contents 2

Methods and Equipment 4

1 Synthetic Procedures 7

1.1 Starting Material Syntheses 7

1.2 Syntheses of Imine-linked COFs 13

1.3 Syntheses of Amine-linked COFs 16

1.4 Syntheses of Hybrid Materials 20

2 Analytical Data 23

2.1 FTIR Spectroscopy 23

2.2 X-Ray Powder Diffraction 28

2.2.1 Rietveld Refinements for Imine-linked COFs 28

2.2.2 Rietveld Refinements for Amine-linked COFs 30

2.2.3 Coherence Length Analysis 32

2.2.4 Additional XRPD Patterns 34

2.3 ssNMR Spectroscopy 36

2.3.1 ssNMR Spectra of Imine-linked COFs 36

2.3.2 ssNMR Spectra of Amine-linked COFs 41

2.3.3 ssNMR Spectra of Hybrid Materials 47

2.3.4 Additional ssNMR Spectra 50

2.4 N_2 Adsorption Data 53

2.4.1 Sorption Isotherms of Imine-linked COFs 53

2.4.2 Sorption Isotherms of Amine-linked COFs 55

2.4.3 Pore Size Distribution in Imine-Linked COFs 58

2.4.4 Pore Size Distribution in Amine-linked COFs 60

2.4.5 BET Plots 63

2.5 Scanning Electron Microscopy 69

2.6 Transmission Electron Microscopy 72

2.7 Stability Tests 75

3 Quantum-Chemical Calculations 77

3.1 Geometry Optimization 77

3.2 NMR Chemical Shifts 81

4 Pair Distribution Function (PDF) Analysis 90

4.1 Methods 90

4.1.1 Data processing 90

4.1.2 Real-space structure refinement 91

4.2 Results 92

References 103

Methods and Equipment

General methods: All reactions, unless otherwise noted, were performed with magnetic stirring under Ar atmosphere using standard Schlenk techniques. Reaction temperatures were electronically monitored as external heating block temperatures. Reagents were purchased from different commercial sources and used without further purification. The removal of solvent under reduced pressure was carried out on a standard rotary evaporator.

Solvents: THF was distilled with sodium and benzophenone under inert gas prior to use. Degassed solvents were degassed by purging with Ar for at least 10 min. Solvents for flash column chromatography were purified by distillation under reduced pressure.

Chromatography: Analytical thin-layer chromatography was carried out on pre-coated aluminum plates (silica gel 60 F254) from Merck. Compound spots were visualized under ultra violet light (UV, 254 nm) for fluorescent compounds or $KMnO_4$ stain solutions. For flash column chromatography, silica gel 60 from Merck, with a particle size between 40 and 63 μm, was used.

NMR spectroscopy: Liquid state ^{1}H-NMR spectra were recorded on a JEOL ECZ 400S 400 MHz spectrometer or a Bruker Avance III HD 400 at 400 MHz, respectively, and are reported as follows: chemical shift δ in ppm (multiplicity, coupling constants *J* in Hz, number of protons, assignment). Couplings are expressed as: s (singlet), d (doublet), t (triplet), q (quartet), m (multiplet) or combinations. All chemical shifts δ are reported to the nearest 0.01 ppm with the residual solvent peak as the internal reference (chloroform-d = 7.26 ppm, dimethylsulfoxide-d^6 = 2.50 ppm). ^{13}C-NMR spectra were recorded on the same spectrometers at 101 MHz with ^{1}H decoupling, respectively. All ^{13}C resonances are reported to the nearest 0.1 ppm with the central resonance of the solvent peak as the internal reference (chloroform-d = 77.16 ppm, dimethylsulfoxide-d^6 = 39.52 ppm). In order to allocate proton and carbon spectra, several 2D NMR spectra (COSY, HSQC, and HMQC) were used. The numbering of the proton and carbon atoms does not match the IUPAC nomenclature. Diastereotopic protons in the ^{1}H NMR spectra are referenced with a and b: this nomenclature is arbitrary and does not correspond to the spin system.

Solid state NMR spectra were recorded at room temperature in 4 mm or 2.5 mm OD ZrO_2 rotors on a Bruker Avance III 400 MHz spectrometer using Bruker BL4 or BL2.5 double resonance MAS probes. Standard instrument library pulse sequences were used. Chemical shifts were referenced relative to TMS (^{1}H, 0.0 ppm), adamantane (^{13}C, 38.52 ppm and 29.47 ppm), and CH_3NO_2 (^{15}N, 0.0 ppm).

Infrared spectroscopy: IR spectra were recorded on a Perkin Elmer UATR Two FT-IR spectrometer equipped with an attenuated total reflection (ATR) measuring unit. IR data are reported in wavenumbers (cm^{-1}) of normalized absorption. The IR bands are characterized as w (weak), m (medium), s (strong), or br (broad).

High-resolution mass spectrometry (HRMS): High-resolution electron spray ionization (ESI) mass spectra were recorded on a Varian MAT 711 MS spectrometer.

SEM/EDX: SEM SE (secondary electron) detector images were obtained on either a Zeiss Merlin or a VEGA TS 5130MM (TESCAN) with a SEM-EDX using a Si/Li detector (Oxford).

TEM/EDX was performed with a Philips CM30 ST (300kV, LaB6 cathode). The samples were prepared dry onto a copper lacey carbon grid (Plano). EDX was obtained on a Noran System Seven (NSS) Si(Li) detector.

Sorption: Sorption measurements were performed on a Quantachrome Instruments Autosorb iQ MP with Nitrogen at 77 K. The samples were degassed for 12 h at 120 °C under vacuum prior to the gas adsorption studies. Pore size distribution was determined from Nitrogen adsorption isotherms using the QSDFT cylindrical pores in carbon model for nitrogen at 77 K. For multipoint BET surface area calculations, pressure ranges were chosen with the help of the BET assistant in the ASiQwin software, which chooses BET tags in accordance with the ISO recommendations equal or below the maximum in grams per square meter.

X-ray powder diffraction (XRPD): X-ray powder diffraction experiments were performed on a Stoe Stadi P diffractometer (Cu-Kα1, Ge(111) in Debye-Scherrer geometry. The samples were measured in sealed glass capillaries (OD = 0.7 mm) and spun for improved particle statistics.

Rietveld refinements: Rietveld refinements were performed using TOPAS v6. The background was corrected with Chebychev polynomials (Order 5). Simple axial and zero-error corrections were used together with additional corrections for Gaussian and Lorentzian crystallite size and strain broadening.

Synchrotron x-ray total scattering measurements: Total scattering measurements were carried out using the high energy Powder Diffraction and Total Scattering Beamline P02.1 of PETRA III at the Deutsches Elektronen-Synchrotron (DESY). X-ray total scattering data were collected in rapid acquisition mode (RAPDF) [1]. A large-area 2D PerkinElmer XRD1621 detector (2048×2048 pixels, 200×200 μm^2 each) was used at a sample-to-detector distance of approximately 304 mm. Samples were loaded into 1.8 mm ID/1.9 mm OD polyimide capillaries (Cole-Parmer) and measured at room temperature. The incident energy of the x-rays was 59.858 keV (λ =0.20713 Å). A measurement of Si was collected at room temperature as a standard for calibration of the setup. Calibration was performed, and the raw 2D intensity was corrected for polarization and azimuthally integrated and converted to 1D intensity versus Q ($Q = 4\pi \sin \theta/\lambda$ is the magnitude of the scattering momentum transfer, with 2θ scattering angle) using the software Fit2D[2].

Further correction and normalization of the integrated 1D diffraction intensities were carried out to obtain the total scattering structure function, F(Q), which was Fourier transformed to

obtain the pair distribution function (PDF), G(r) using PDFgetX3[3] within xPDFsuite[4]. The maximum value used in the Fourier transform of the total scattering data (Qmax) was 22.0 $Å^{-1}$. Real-space structure model refinements to the PDF data were carried out using PDFgui[5]. The resolution parameters due to Q-space resolution, Qdamp = 0.0340 $Å^{-1}$, and due to variable Q-resolution and high-Q noise, Qbroad = 0.0064 $Å^{-1}$, were determined from Si and used for further fitting.

Supercritical CO_2 activation: Activation of the methanol-soaked COF samples with supercritical CO_2 was performed on a Leica EM CPD300 critical point dryer.

Quantum-Chemical Calculations: Atom positions and lattices of all periodic structures were optimized on RI-PBE-D3/def2-TZVP[6, 7, 8, 9] level of theory using an acceleration scheme based on the resolution of the identity (RI) technique and the continuous fast multipole method (CFMM)[10, 11, 12] implemented[13, 14] in Turbomole[15] version V7.3.

The CFMM uses multipole moments of maximum order 20, together with a well-separateness value of 3 and a basis function extent threshold of 10^{-9} a.u. Grid 7 was used for the numerical integration of the exchange-correlation term. The norm of the gradient was converged to 10^{-4} a.u. and the total energy to 10^{-8} Hartree within the structure optimization using the gamma point approximation.

Structures for all investigated molecular compounds were optimized on PBE0-D3/def2-TZVP[7, 8, 16, 17] level of theory. Subsequent frequency calculations were performed on the same level of theory to ensure all minima to be true minima on the potential energy hypersurface.

NMR chemical shifts were obtained on B97-2/pcS-2[18, 19] level of theory using the FermiONs++[18, 20, 21] program package.

1 Synthetic Procedures

1.1 Starting Material Syntheses

N-(4-cyanophenyl)acetamide

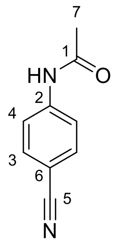

4-aminobenzonitrile (2.00 g, 16.8 mmol, 1.0 eq.) was dissolved in 1.2 M aqueous HCl (20 mL) and cooled to 0°C in an ice bath. After addition of 4.2 M aqueous NaOAc (7 mL) and acetic anhydride (7.4 mL, 78.3 mmol, 4.1 eq.), the solution was stirred at 0°C for 20 min. The reaction mixture was diluted with water (50 mL). Suction filtration of the precipitate and washing with water (2 x 50 mL) afforded *N*-(4-cyanophenyl)acetamide (2.53 g, 15.8 mmol, 93%) as a white solid.

^{1}H NMR (400 MHz, $CDCl_3$): δ = 7.68 – 7.57 (m, 4H, H-3, H-4), 7.39 (s, 1H, NH), 2.22 (s, 3H, H-7) ppm.

^{13}C NMR (101 MHz, $CDCl_3$): δ = 168.6 (C-1), 142.0 (C-2), 133.5 (C-3), 119.6 (C-4), 118.9 (C-5), 107.3 (C-6), 24.9 (C-7) ppm.

IR (ATR): $\tilde{\nu}$ = 3300 (w), 3258 (w), 3184 (w), 3114 (w), 3054 (w), 2222 (m), 1667 (s), 1598 (s), 1541 (s), 1506 (m), 1404 (m), 1362 (m), 1322 (s), 1265 (m), 833 (s) cm^{-1}.

HRMS (ESI): calculated for $C_9H_7N_2O^-$ ($[M-H]^-$): 159.05639.

found: 159.05635.

The analytical data matched those previously described in the literature.[22]

4,4',4''-(1,3,5-triazine-2,4,6-triyl)trianiline

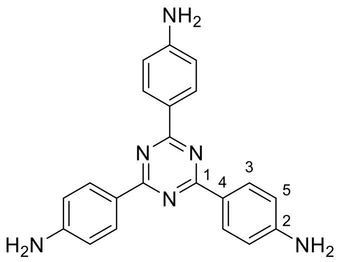

Procedure A from *N*-(4-cyanophenyl)acetamide:

A heat-dried round-bottom flask was equipped with chlorosulfonic acid (5.4 mL, 80.1 mmol, 5.0 eq.) and cooled to –15°C. *N*-(4-cyanophenyl)acetamide (2.50 g, 15.6 mmol, 1.0 eq.) was added to the stirring acid in small portions. The resulting slurry was allowed to warm to room temperature under stirring for 16 h. The reaction mixture was poured on ice and neutralized to pH 7 with 4 M aqueous NaOH. Suction filtration of the precipitate and washing with water (2 x 50 mL) afforded crude N,N',N''-((1,3,5-triazine-2,4,6-triyl)tris(benzene-4,1-diyl))triacetamide (2.50 g) as an orange solid, which was used without further purification in the next step.

To a solution of the crude triacetamide (2.50 g) in EtOH (32 mL) conc. aqueous HCl (12 mL) was added and the reaction mixture was heated to 110°C for 16 h. The reaction mixture was neutralized to pH 7 with 4 M aqueous NaOH. Suction filtration of the precipitate and washing with water (2 x 30 mL) afforded 4,4',4''-(1,3,5-triazine-2,4,6-triyl)trianiline (665 mg, 1.88 mmol, 36%) as a yellow solid.

Procedure B from 4-aminobenzonitrile:

A heat-dried round-bottom flask was equipped with 4-aminobenzonitrile (772 mg, 6.54 mmol, 1.0 eq.) and cooled to 0°C. Trifluoromethanesulfonic acid (2.0 mL, 22.6 mmol, 3.5 eq.) was added slowly through a Teflon cannula. The resulting slurry was allowed to warm to room temperature for 24 h. The reaction mixture was diluted with water (20 mL) and neutralized to pH 7 with 2 M aqueous NaOH. Suction filtration of the precipitate and washing with water (2 x 30 mL) afforded 4,4',4''-(1,3,5-triazine-2,4,6-triyl)trianiline (494 mg, 1.39 mmol, 64%) as a yellow solid.

^{1}H NMR (400 MHz, DMSO-d$_6$): δ = 8.35 (d, J = 8.8 Hz, 6H, H-3), 6.69 (d, J = 8.8 Hz, 6H, H-5), 5.89 (s, 6H, NH_2) ppm.

^{13}C NMR (101 MHz, DMSO-d$_6$): δ = 170.0 (C-1), 153.4 (C-2), 130.6 (C-3), 123.4 (C-4), 113.6 (C-5) ppm.

IR (ATR): $\tilde{\nu}$ =3462 (w), 3325 (w), 3210 (w), 1632 (m), 1605 (m), 1578 (m), 1495 (s), 1431 (m), 1309 (m), 1295 (m), 1179 (m), 1148 (m), 812 (s) cm^{-1}.

HRMS (ESI): calculated for $C_{21}H_{19}N_6^+$ ([M+H]$^+$): 355.16657

found: 355.16649.

The analytical data matched those previously described in the literature.[22, 23]

2,4,6-tris(4-bromophenyl)-1,3,5-triazine

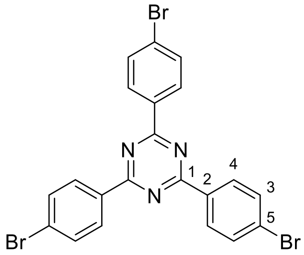

A heat-dried round-bottom flask was equipped with 4-Bromobenzonitrile (1.0 g, 5.5 mmol, 0.3 eq.) and cooled to 0°C. Trifluoromethanesulfonic acid (2.0 mL, 22 mmol, 4.0 eq.) was added slowly through a Teflon cannula. The resulting slurry was allowed to warm to room temperature for 24 h. The reaction mixture was diluted with water (20 mL) and neutralized to pH 7 with 2 M aqueous NaOH. Suction filtration of the precipitate and washing with water (2 x 10 mL) afforded 2,4,6-tris(4-bromophenyl)-1,3,5-triazine (0.93 g, 1.8 mmol, 93%) as a white solid.

^{1}H NMR (400 MHz, CDCl$_3$): δ = 8.59 (d, J = 8.5 Hz, 6H, H-4), 7.70 (d, J = 8.5 Hz, 6H, H-3) ppm.

^{13}C NMR (101 MHz, CDCl$_3$): δ = 171.3 (C-1), 135.0 (C-2), 132.2 (C-3), 130.7 (C-4), 123.7 (C-5) ppm.

IR (ATR): $\tilde{\nu}$ = 1579 (m), 1511 (s), 1401 (m), 1354 (m), 1172 (m), 1148 (w), 1101 (w), 1067 (m), 1009 (s), 842 (m), 802 (s), 629 (w), 495 (s) cm^{-1}.

HRMS (EI): calculated for $C_{21}H_{12}Br_3N_3^{\cdot-}$ ([M]$^{\cdot-}$): 542.8587

found: 542.8578.

The analytical data matched those previously described in the literature.[24]

4,4',4''-(1,3,5-triazine-2,4,6-triyl)tribenzaldehyde

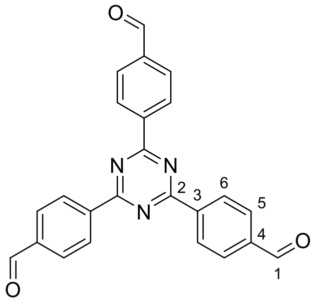

To a suspension of 2,4,6-tris(4-bromophenyl)-1,3,5-triazine (0.50 g, 0.92 mmol, 1.0 eq.) in THF (50 mL), *n*-BuLi (2.5 M in hexane, 1.2 mL, 3.0 mmol, 3.3 eq.) was added dropwise at -78°C. The mixture was stirred at -78°C for 90 min, then 1-formylpiperidine (0.34 mL, 3.0 mmol, 3.3 eq.) was added dropwise at -78°C. The mixture was stirred at -78°C for 30 min and allowed to warm to room temperature. Aqueous concentrated NH_4Cl solution (1 mL) was added and the solvent was removed under reduced pressure. The residue was suspended in a mixture of EtOH (3 mL) and water (3 mL). Suction filtration of the suspension and washing with water (10 mL) and EtOH (10 mL) afforded 4,4',4''-(1,3,5-triazine-2,4,6-triyl)tribenzaldehyde (0.35 g, 0.91 mmol, 97 %) as an off-white solid.

^{1}H NMR (400 MHz, $CDCl_3$): δ = 10.18 (s, 3H, H-1), 8.92 (d, J = 8.2 Hz, 6H, H-6), 8.37 – 7.82 (m, 6H, H-5) ppm.

^{13}C NMR (101 MHz, $CDCl_3$): δ = 192.0 (C-1), 140.9 (C-2), 139.5 (C-3), 130.1 (C-4), 129.8 (C-5) ppm.

IR (ATR): $\tilde{\nu}$ = 1699 (m), 1582 (w), 1515 (s), 1359 (m), 1298 (w), 1201 (m), 1105 (w), 1013 (w), 805 (s), 692 (w), 498 cm^{-1}.

**HRMS** (EI):	calculated for $C_{24}H_{15}N_3O_3^{\cdot+}$ ($[M]^{\cdot+}$):	393.1108
	found:	393.1107.

The analytical data matched those previously described in the literature.[25]

4,4',4'',4'''-(pyrene-1,3,6,8-tetrayl)tetraaniline

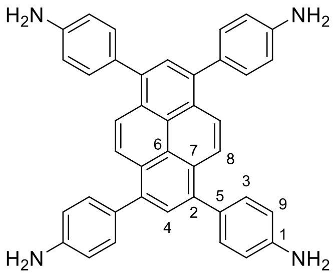

Tetrakis(triphenylphosphine)palladium(0) (0.40 g, 0.35 mmol, 1.0 meq.) was added to a mixture of (1,3,6,8-tetrabromopyrene (3.00 g, 5.8 mmol, 1.0 eq.), 4-aminophenylboronic acid pinacol ester (5.98 g, 27.3 mmol, 4.0 eq.) and K_2CO_3 (4.40 g, 31.8 mmol, 5.5 eq.) in 1,4-dioxane (135 mL) and water (45 mL). After refluxing at 110°C for 72 h, the mixture was allowed to cool to room temperature. Suction filtration of the suspension and washing with water (50 mL) and MeOH (50 mL) afforded 4,4',4'',4'''-(pyrene-1,3,6,8-tetrayl)tetraaniline (2.83 g, 5.78 mmol, 86 %) as a yellow solid.

^{1}H NMR (400 MHz, DMSO-d$_6$): δ = 8.12 (s, 4H, H-8), 7.78 (s, 2H, H-4), 7.34 (d, *J* = 7.9 Hz, 8H, H-3), 6.77 (d, *J* = 7.9 Hz, 8H, H-9), 5.30 (s, 8H, NH_2).ppm.

^{13}C NMR (101 MHz, DMSO-d$_6$): δ = 148.2 (C-1), 137.1 (C-2), 131.0(C-3), 129.0(C-4), 127.6(C-5), 126.7(C-6), 126.1(C-7), 124.4(C-8), 113.9 (C-9) ppm.

IR (ATR): $\tilde{\nu}$ = 3438 (w), 3417 (w), 3338 (w), 3210 (w), 2858 (w), 1605 (m), 1517 (m), 1493 (m), 1459 (w), 1275 (m), 1176 (m), 1061 (m), 904 (w), 834 (vs), 738 (w), 638 (m), 606 (m), 577 (m), 530 (s), 497 (s) cm^{-1}.

HRMS (ESI): calculated for $C_{40}H_{31}N_4^+$ ($[M+H]^+$): 567.2543

found: 567.2591.

The analytical data matched those previously described in the literature.[26]

1.2 Syntheses of Imine-linked COFs

PI-3-COF

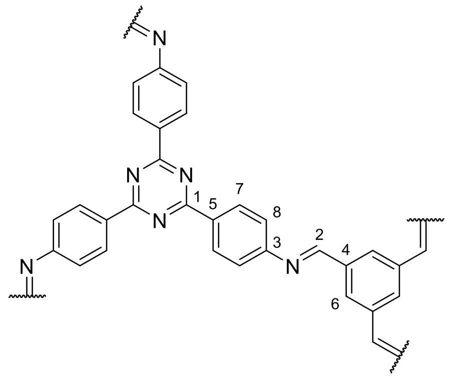

To a mixture of benzene-1,3,5-tricarbaldehyde (22.1 mg, 0.13 mmol, 1.0 eq.) and 4,4',4"-(1,3,5-triazine-2,4,6-triyl)trianiline (46.8 mg, 0.13 mmol, 1.0 eq.) in mesitylene (2.7 mL) and 1,4-dioxane (1.3 mL), aqueous 6 M AcOH (0.5 mL) was added. The suspension was heated at 120°C for 72 h. Suction filtration of the precipitate and washing with DMF (20 mL), THF (20 mL) and DCM (20 mL) afforded PI-3-COF (55.5 mg, 91%) as a yellow solid.

^{1}H ssNMR (MAS, 400 MHz): δ = 7.1 (H-2, H-6, H-7, H-8) ppm.

^{13}C ssNMR (CP-MAS, 101 MHz): δ = 169.1 (C-1), 155.3 (C-2), 151.5 (C-3), 136.6 (C-4), 134.0 (C-5), 129.6 (C-6), 127.7 (C-7), 116.3 (C-8) ppm.

^{15}N ssNMR (CP-MAS, 41 MHz): δ = -59.0 (N-2), -131.8 (N-1) ppm.

IR (ATR): $\tilde{\nu}$ = 1579 (m), 1511 (s), 1413 (m), 1370 (s), 1174 (w), 1141 (w), 1013 (w), 968 (w), 864 (w), 813 (s), 678 (w), 531 (w), 415 (w) cm^{-1}.

The analytical data matched those previously described in the literature.[27]

TTI-COF

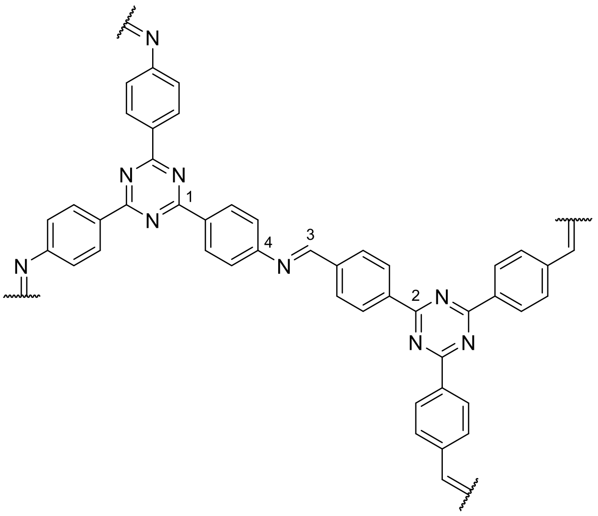

To a mixture of 4,4',4''-(1,3,5-triazine-2,4,6-triyl)tribenzaldehyde (25.0 mg, 63.5 µmol, 1.0 eq.) and 4,4',4''-(1,3,5-triazine-2,4,6-triyl)trianiline (22.5 mg, 63.5 µmol, 1.0 eq.) in mesitylene (2.5 mL) and 1,4-dioxane (2.5 mL), aqueous 6 M AcOH (0.13 mL) was added. The suspension was heated at 120°C for 72 h. Suction filtration of the precipitate and washing with DMF (5 mL), THF (5 mL) and DCM (5 mL) afforded TTI-COF (26.8 mg, 61%) as a yellow solid.

^{1}H ssNMR (MAS, 400 MHz): δ = 7.1 (H-2, H-Ar) ppm.

^{13}C ssNMR (CP-MAS, 101 MHz): δ = 169.1 (C-1, C-2), 157.4 (C-3), 151.7 (C-4), 138.5 (C-Ar), 133.9 (C-Ar), 128.2 (C-Ar), 114.8 (C-Ar) ppm.

^{15}N ssNMR (CP-MAS, 41 MHz): δ = -55.7 (N-3), -131.3 (N-1, N-2) ppm.

IR (ATR): $\tilde{\nu}$ = 1576 (w), 1505 (vs), 1414 (m), 1362 (s), 1173 (w), 1145 (w), 1014 (w), 857 (w), 814 (m), 595 (w), 544 (w) cm^{-1}.

The analytical data matched those previously described in the literature.[28]

Py1P-COF

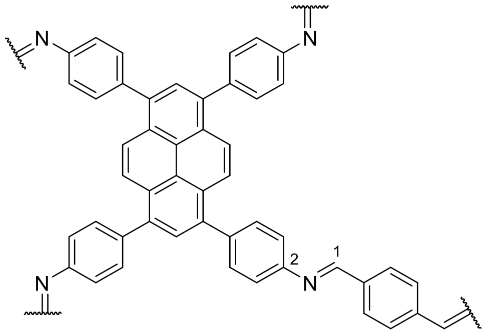

To a mixture of 4,4',4'',4'''-(pyrene-1,3,6,8-tetrayl)tetraaniline (34.0 mg, 60.0 μmol, 1.0 eq.) and terephthalaldehyde (16.1 mg, 0.12 mmol, 2.0 eq.) in mesitylene (2 mL) and 1,4-dioxane (1 mL), aqueous 6 M AcOH (0.3 mL) was added. The suspension was heated at 120°C for 7 days. Suction filtration of the precipitate and washing with MeOH (5 mL), DMF (5 mL), THF (5 mL) and DCM (5 mL) afforded Py1P-COF (37.9 mg, 83%) as an orange solid.

^{1}H ssNMR (MAS, 400 MHz): δ = 6.9 (H- 1, H-Ar) ppm.

^{13}C ssNMR (CP-MAS, 101 MHz): δ = 156.5 (C-1), 149.8 (C-2), 138.7 (C-Ar), 137.3 (C-Ar), 132.8 (C-Ar), 131.5 (C-Ar), 129.4 (C-Ar), 127.8 (C-Ar), 124.9 (C-Ar), 121.7 (C-Ar), 119.4 (C-Ar) ppm.

^{15}N ssNMR (CP-MAS, 41 MHz): δ = -48.8 (N-1) ppm.

IR (ATR): $\tilde{\nu}$ = 3031 (w), 1699 (w), 1625 (m), 1597 (w), 1489 (m), 1460 (w), 1303 (w), 1198 (w), 1169 (w), 1104 (w), 1005 (w), 967 (w), 882 (w), 831 (s), 737 (w), 613 (w), 585 (w), 538 (w) cm^{-1}.

The analytical data matched those previously described in the literature.[26]

1.3 Syntheses of Amine-linked COFs

rPI-3-COF

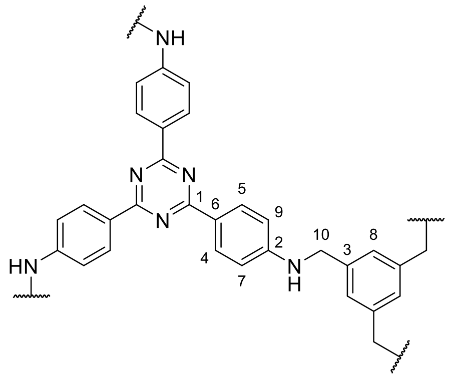

Procedure A: Reduction of PI-3-COF

To a suspension of PI-3-COF (30.0 mg) in mesitylene (2 mL) and 1,4-dioxane (1 mL), formic acid (97%, 53.0 µL) was added. The suspension was heated at 120°C for 48 h. The precipitate was collected via suction filtration and extracted with MeOH in a Soxhlet extractor for 12 h. Extraction with supercritical CO_2 afforded rPI-3-COF (28.0 mg, 92%) as a yellow solid.

Procedure B: One-pot strategy

To a mixture of benzene-1,3,5-tricarbaldehyde (9.7 mg, 60 µmol, 1.0 eq.) and 4,4',4''-(1,3,5-triazine-2,4,6-triyl)trianiline (21 mg, 60 µmol, 1.0 eq.) in mesitylene (2 mL) and 1,4-dioxane (1 mL), formic acid (97%, 49 µL) was added. The suspension was heated at 60°C for 48 h and at 120°C for 24 h. Suction filtration of the precipitate and washing with DMF, THF and DCM (3 x 5 mL) afforded rPI-3-COF (26 mg, 92%) as a yellow solid.

^{1}H ssNMR (MAS, 400 MHz): δ = 7.8 (H-4, H-5), 5.9 (H-7, H-8, H-9), 2.6 (H-10) ppm.

^{13}C ssNMR (CP-MAS, 101 MHz): δ = 168.8 (C-1), 151.0 (C-2), 137.3 (C-3), 128.7 (C-4, C-5), 126.1 (C-6), 119.0 (C-7), 114.4 (C-8), 111.2 (C-9), 45.4 (C-10) ppm.

^{15}N ssNMR (CP-MAS, 41 MHz): δ = -141.4 (N-1), -313.3 (N-10) ppm.

IR (ATR): $\tilde{\nu}$ = 3412 (w), 1607 (w), 1581 (w), 1509 (s), 1412 (m), 1369 (s), 1326 (w), 1261 (w), 1182 (m), 1145 (m), 1012 (w), 863 (w), 809 (m), 682 (w), 651 (w), 512 (w)cm^{-1}.

disordered rPI-3-COF

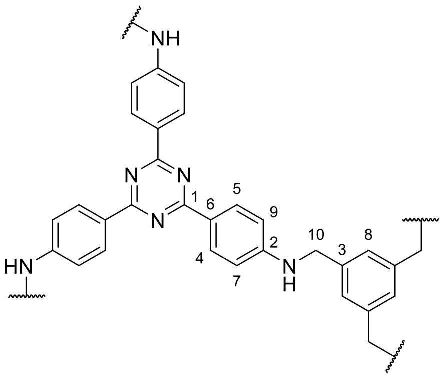

Procedure A: To a suspension of PI-3-COF (10.0 mg) in mesitylene (666 μL) and 1,4-dioxane (333 μL), formic acid (97%, 50.0 μL) was added. The suspension was stirred at 120°C for 48 h. The precipitate was collected via suction filtration, washed with DMF, THF, and DCM (3 x 2 mL) and dried under high vacuum. Disordered rPI-3-COF (9.9 mg, 98%) was obtained as a yellow solid.

Procedure B: A suspension of pfrPI-3-COF (10.0 mg) in aqueous 1M HCl (2 mL) was stirred at 120°C for 20 min. Suction filtration, washing with water, THF, DCM (3 x 2 mL) and drying under high vacuum afforded disordered rPI-3-COF (9.2 mg) as a yellow solid.

^{1}H ssNMR (MAS, 400 MHz): δ = 7.3 (H-4, H-5, H-7, H-9, H-8), 3.4 (H-10) ppm.

^{13}C ssNMR (CP-MAS, 101 MHz): δ = 170.3 (C-1), 151.5 (C-2), 139.9 (C-3), 130.0 (C-4, C-5, C-6), 114.5 (C-7, C-8, C-9), 46.5 (C-10) ppm.

IR (ATR): $\tilde{\nu}$ = 3371 (w), 1606 (m), 1509 (vs), 1472 (s), 1367 (s), 1321 (m), 1263 (m), 1181 (m), 1145 (m), 807 (s), 573 (w), 521 (w) cm^{-1}.

rTTI-COF

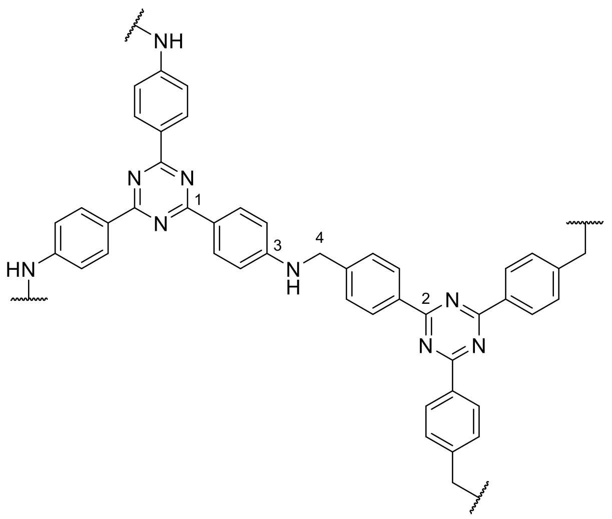

To a suspension of TTI-COF (30.0 mg) in mesitylene (2 mL) and 1,4-dioxane (1 mL), formic acid (97%, 19.3 µL) was added. The suspension was heated at 120°C for 48 h. The precipitate was collected via suction filtration and extracted with MeOH in a Soxhlet extractor for 12 h. Extraction with supercritical CO_2 afforded rTTI-COF (27.1 mg, 90%) as a yellow solid.

^{1}H ssNMR (MAS, 400 MHz): δ = 7.4 (H-Ar), 3.0 (H-4) ppm.

^{13}C ssNMR (CP-MAS, 101 MHz): δ = 170.5 (C-1, C-2), 151.5 (C-3), 138.8 (C-Ar), 134.5 (C-Ar), 128.9 (C-Ar), 114.0 (C-Ar), 46.7 (C-4) ppm.

^{15}N ssNMR (CP-MAS, 41 MHz): δ = -134.0 (N-1, N-2), -314.8 (N-4) ppm.

IR (ATR): $\tilde{\nu}$ = 3403 (w), 1607 (w), 1581 (w), 1505 (s), 1412 (m), 1359 (s), 1258 (w), 1180 (w), 1145 (w), 1016 (w), 855 (w), 809 (m), 592 (w), 541 (w) cm^{-1}.

rPy1P-COF

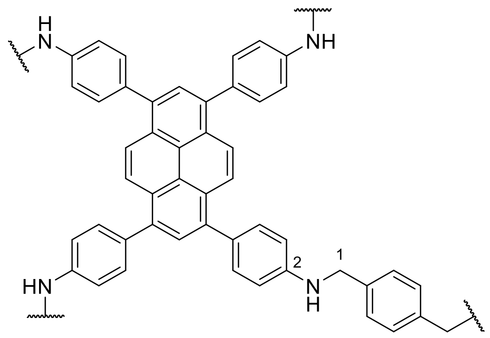

To a suspension of Py1P-COF (15.0 mg) in mesitylene (2 mL) and 1,4-dioxane (1 mL), formic acid (97%, 13.0 μL) was added. The suspension was heated at 120°C for 24 h. The precipitate was collected via suction filtration and extracted with MeOH in a Soxhlet extractor for 12 h. Extraction with supercritical CO_2 afforded rPy1P-COF (14.2 mg, 94%) as an orange solid.

^{1}H ssNMR (MAS, 400 MHz): δ = 6.9 (C-Ar), 2.9 (C-1) ppm.

^{13}C ssNMR (CP-MAS, 101 MHz): δ = 147.6 (C-2), 137.5 (C-Ar), 129.6 (C-Ar), 127.8 (C-Ar), 114.5 (C-Ar), 112.2, 48.2 (C-1) ppm.

^{15}N ssNMR (CP-MAS, 41 MHz): δ = -317.6 (N-1) ppm.

IR (ATR): $\tilde{\nu}$ = 3397 (w), 3028 (w), 1609 (s), 1520 (m), 1491 (s), 1461 (w), 1319 (w), 1291 (w), 1250 (w), 1180 (w), 1016 (w), 980 (w), 821 (vs), 732 (w), 611 (w), 579 (w), 545 (w), 524 (w) cm^{-1}.

1.4 Syntheses of Hybrid Materials

prPy1P-COF

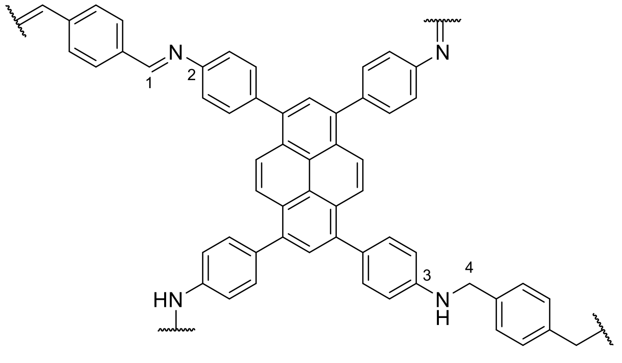

In an representative example, partial reduction of Py1P-COF (15.0 mg) was performed according to the procedure for rPy1P-COF with a reduced amount of formic acid (97%, 12.3 μL) at 120°C for 24h, affording prPy1P-COF (14.2 mg, 94%) as an orange solid. Estimated from the relative integrals of C-2 and C-3 in the ^{13}C-ssNMR spectrum (see Figure S40 for details), approximately 42% of the imine-bonds were reduced.

^{1}H ssNMR (MAS, 400 MHz): δ = 7.1 (C-1, C-Ar), 3.01 (C-4) ppm.

^{13}C ssNMR (CP-MAS, 101 MHz): δ = 156.7 (C-1), 149.0 (C-2), 146.4 (C-3), 142.1 (C-Ar), 138.4 (C-Ar), 135.9 (C-Ar), 132.1 (C-Ar), 130.1 (C-Ar), 127.7 (C-Ar), 125.1 (C-Ar), 121.3 (C-Ar), 112.7 (C-Ar), 48.1 (C-4) ppm.

^{15}N ssNMR (CP-MAS, 41 MHz): δ = -48.3 (N-1), -316.8 (N-4) ppm.

IR (ATR): $\tilde{\nu}$ = 3397 (w), 3029 (w), 1610 (s), 1521 (m), 1492 (s), 1461 (w), 1319 (w), 1292 (w), 1250 (w), 1180 (w), 1108 (w), 1017 (w), 982 (w), 821 (vs), 732 (w), 611 (w), 580 (w), 543 (w), 519 (w) cm^{-1}.

pfrPI-3-COF

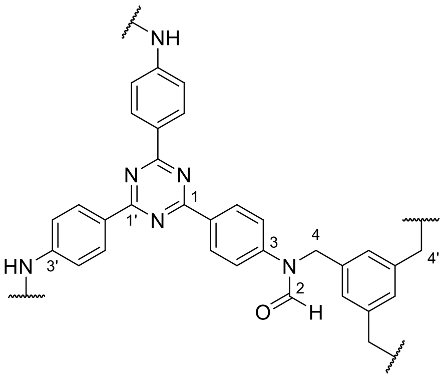

A mixture of PI-3-COF (30.1 mg) and ammonium formate (257 mg, 40.8 mmol) was heated under autogenous pressure at 170°C for 3 h. The solid residue was collected via suction filtration, washed with water, DMF, THF, and DCM (4 x 2 mL) and dried under high vacuum, affording pfrPI-3-COF (31.6 mg) as a yellow solid.

^{1}H ssNMR (MAS, 400 MHz): δ = 7.5 (H-2, H-Ar), 3.7 (H-4, H-4') ppm.

^{13}C ssNMR (CP-MAS, 101 MHz): δ = 170.4 (C-1, C-1'), 162.8 (C-2), 151.9 (C-3, C-3'), 138.0 (C-Ar), 130.2 (C-Ar), 114.9 (C-Ar), 46.5 (C-4, C-4') ppm.

IR (ATR): $\tilde{\nu}$ = 3366 (w), 1669 (w), 1606 (m), 1505 (vs), 1365 (vs), 1327 (m), 1263 (w), 1180 (m), 1145 (m), 811 (m), 640 (w), 587 (w), 520 (w) cm^{-1}.

opfrPI-3-COF

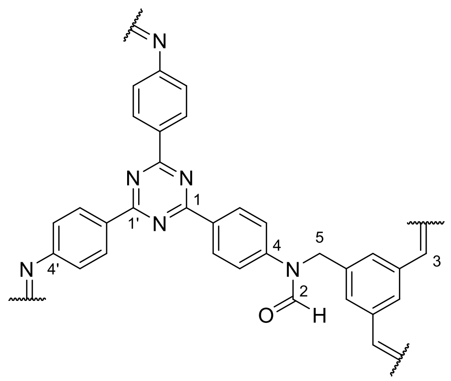

A suspension of pfrPI-3-COF (5.0 mg) and 2,3-Dichloro-5,6-dicyano-p-benzoquinone (15 mg, 64 mmol) in DCM (1 mL) was stirred at r.t. for 24 h. The precipitate was collected via suction filtration, washed with DMF, THF, and DCM (3 x 2 mL) and dried under high vacuum, affording opfrPI-3-COF (4.5 mg) as an orange solid.

^{1}H ssNMR (MAS, 400 MHz): δ = 7.8 (H-2, H-Ar), 5.1 (H-3, H-5) ppm.

^{13}C ssNMR (CP-MAS, 101 MHz): δ = 169.6 (C-1, C-1'), 159.0 (C-2, C-3), 151.8 (C-4, C-4'), 130.30 (C-Ar), 116.73 (C-Ar), 45.64 (C-5) ppm.

IR (ATR): $\tilde{\nu}$ = 1694 (w), 1628 (w), 1579 (w), 1509 (vs), 1413 (m), 1363 (s), 1178 (w), 1144 (w), 1012 (w), 865 (w), 814 (m), 675 (w), 528 (w) cm^{-1}.

BzCl-rTTI-COF

To a suspension of rTTI-COF (3.5 mg) in THF (1 mL), triethylamine (23 µL, 0.17 mmol) and benzoyl chloride (18 µL, 0.15 mmol) was added and the suspension was heated at 60°C for 18h. The precipitate was collected via suction filtration, washed with water, DMF, THF, and DCM (3 x 2 mL) and dried under high vacuum, affording BzCl-rTTI-COF (4.4 mg) as a yellow solid.

TDI-rTTI-COF

A suspension of rTTI-COF (5.0 mg) and toluene diisocyanate (15 µL, 0.11 mmol) in THF (1 mL) was heated at 60°C for 18h. The precipitate was collected via suction filtration, washed with DMF, THF, and DCM (3 x 2 mL) and dried under high vacuum, affording TDI-rTTI-COF (7.1 mg) as a yellow solid.

2 Analytical Data

2.1 FTIR Spectroscopy

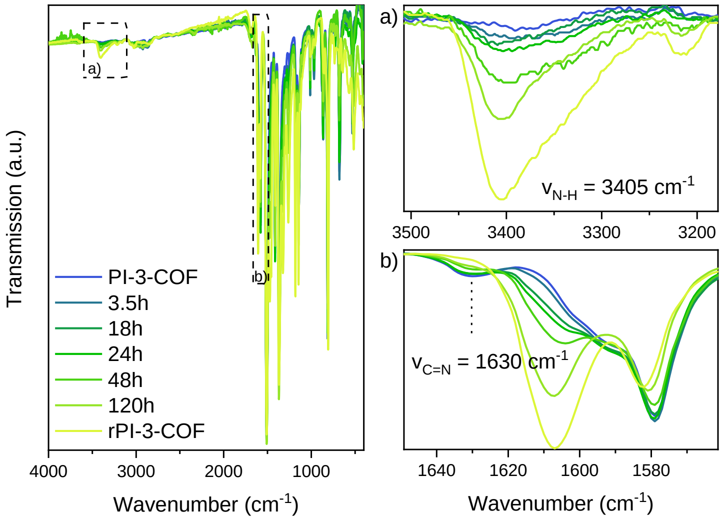

Figure S1: Ex-situ FT-IR analysis of PI-3-COF samples under reductive conditions (19 eq. formic acid, mesitylene:dioxane 2:1, 120°C).

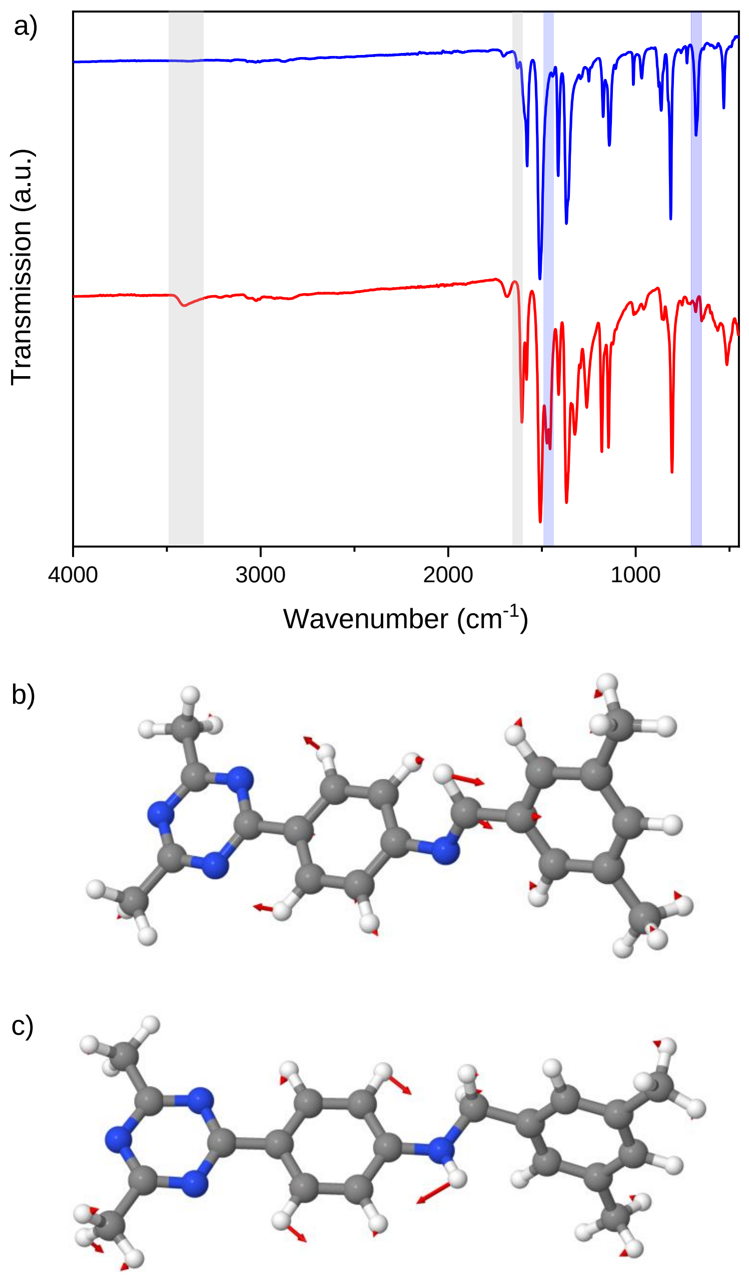

Figure S2: (a) Comparison of FT-IR spectra of PI-3-COF (blue) and rPI-3-COF (red). Grey areas highlight appearing secondary amine and disappearing imine vibrations upon reduction. Blue areas highlight complex vibrations in the fingerprint region, which cannot clearly be assigned to an isolated vibration mode. Calculated IR modes for the molecular models PI-3 M (b) and rPI-3 M (c) in these regions suggest a contribution of imine C-H (b), or amine N-H (c), bending vibrations, respectively.

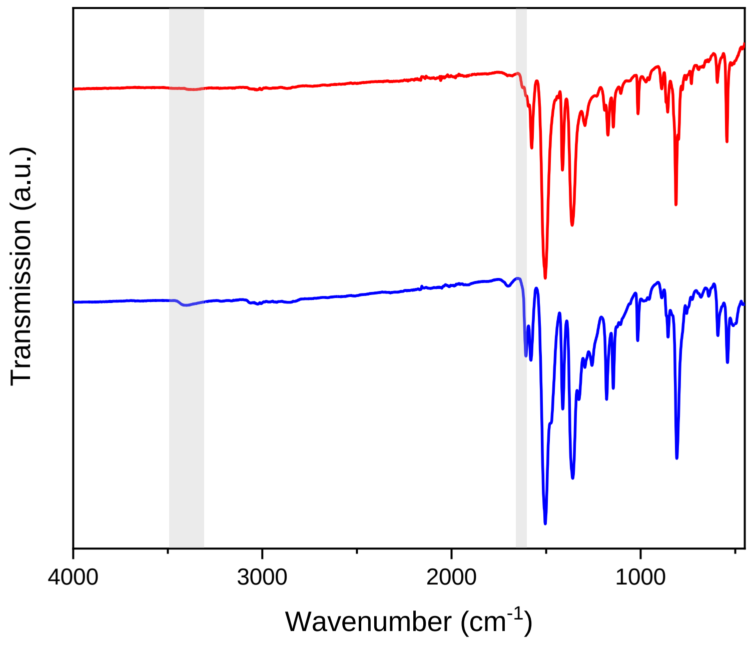

Figure S3: Comparison of FT-IR spectra of TTI-COF (red) and rTTI-COF (blue). Grey areas highlight appearing secondary amine and disappearing imine vibrations upon reduction.

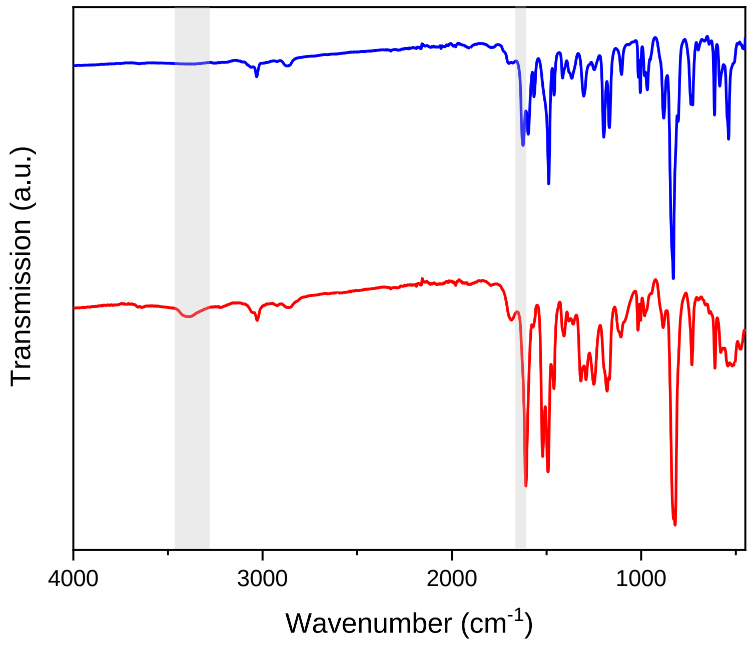

Figure S4: Comparison of FT-IR spectra of Py1P-COF (blue) and rPy1P-COF (red). Grey areas highlight appearing secondary amine and disappearing imine vibrations upon reduction.

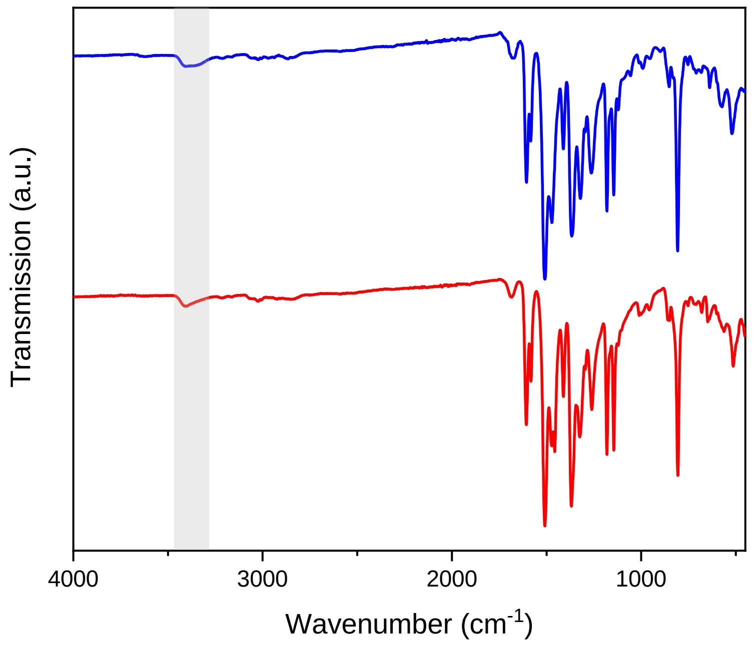

Figure S5: Comparison of FT-IR spectra of disordered rPI-3-COF (blue) and rPI-3-COF (red). Grey area highlights broadened secondary amine vibrations in disordered rPI-3-COF.

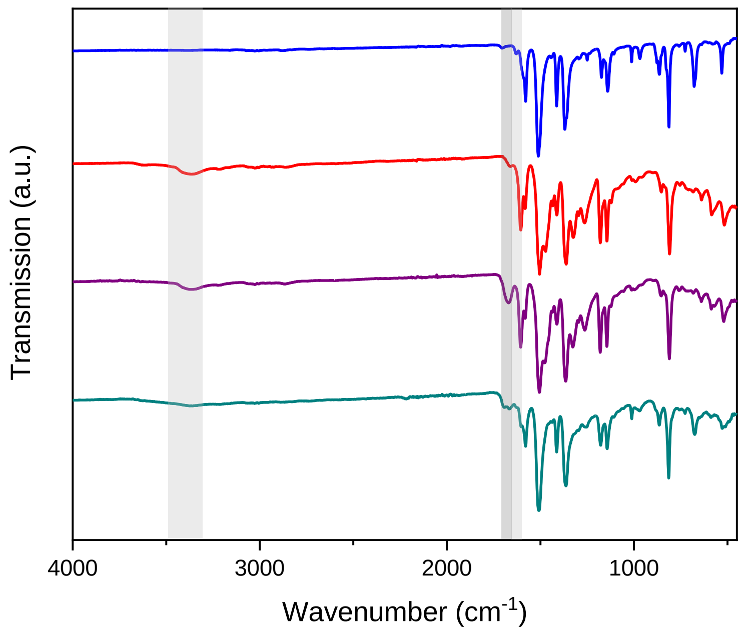

Figure S6: Comparison of FT-IR spectra of PI-3-COF (blue), disordered rPI-3-COF (red), pfrPI-3-COF (purple) and opfrPI-3-COF (cyan). Grey areas highlight secondary amine as well as C=N (imine) and C=O vibrations (dark grey).

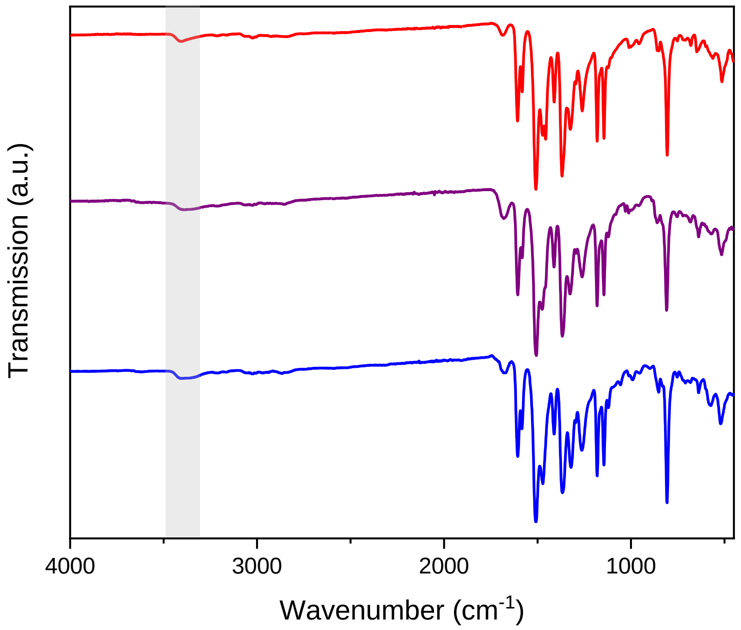

Figure S7: Comparison of FT-IR spectra of rPI-3-COF (red), one-pot synthesized rPI-3-COF (purple) and disordered rPI-3-COF. Grey area highlights broadened secondary amine vibrations in disordered and one-pot rPI-3-COF.

2.2 X-Ray Powder Diffraction

2.2.1 Rietveld Refinements for Imine-linked COFs

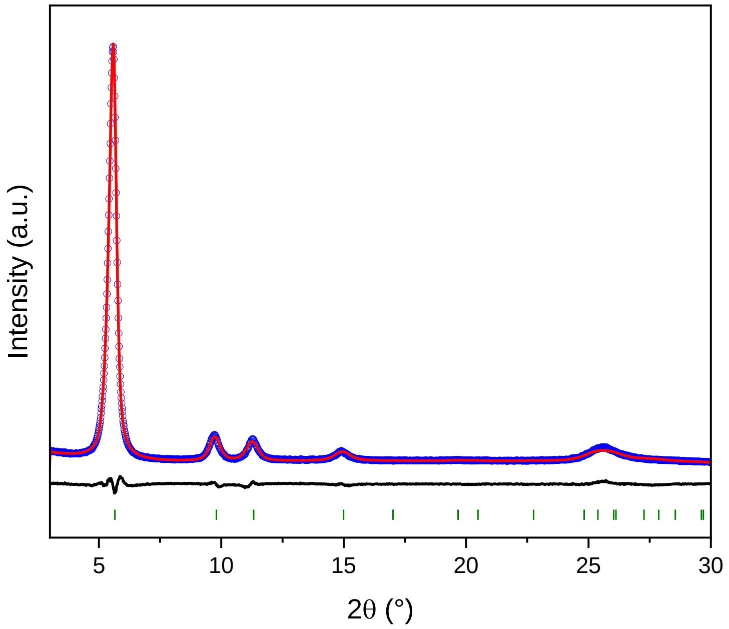

Figure S8: Experimental (blue) and simulated XRPD (Cu-Kα1) diffractogram (red) of PI-3-COF after Rietveld refinement.

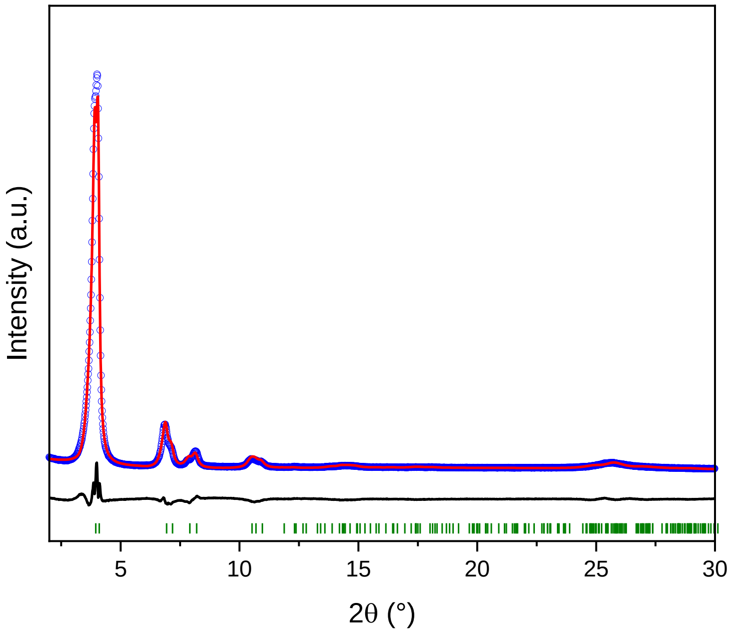

Figure S9: Experimental (blue) and simulated XRPD (Cu-Kα1) diffractogram (red) of TTI-COF after Rietveld refinement.

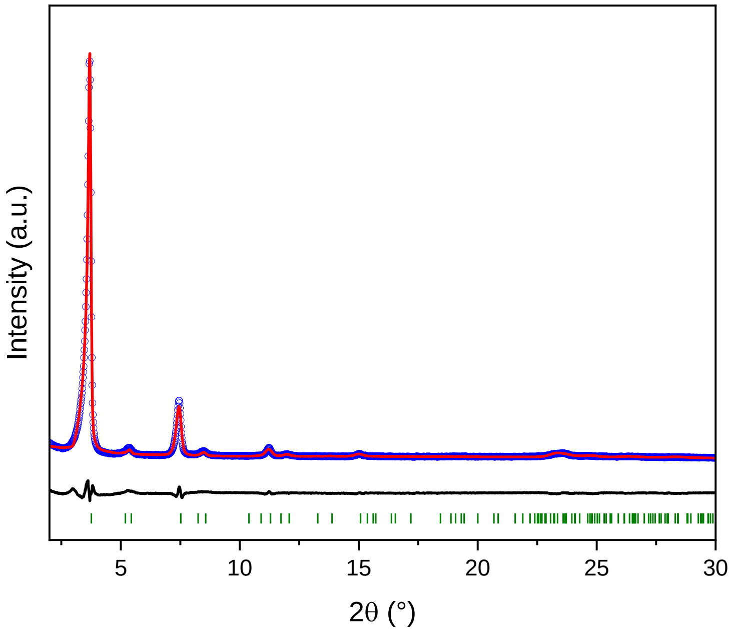

Figure S10: Experimental (blue) and simulated XRPD (Cu-Kα1) diffractogram (red) of Py1P-COF after Rietveld refinement.

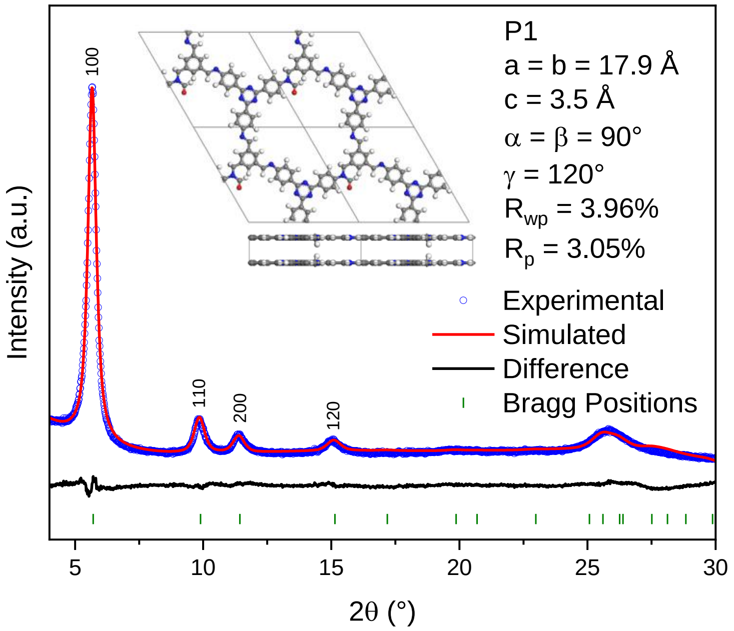

Figure S11: Experimental (blue) and simulated XRPD (Cu-Kα1) diffractogram (red) of opfrPI-3-COF after Rietveld refinement. The conformation and concentration of *N*-formyl groups is arbitrarily and might not represent the real structure influenced by steric effects. The rather small scattering contrast does not allow to refine the exact torsion angle and amount per pore.

Table S1: Cell parameters of Rietveld refined imine-linked COFs. OpfrPI-3-COF was fitted in a constrained $P1$ space group to account for a non-symmetric occurrence of formyl groups in the structure model despite the high apparent symmetry in XRPD diffractogram.

Fitted Pattern	PI-3-COF[27]	opfrPI-3-COF	TTI-COF[29]	Py1P-COF[26]
Space group	$P\bar{6}$	$P1$	$P1$	$C2/m$
Constraints	-	a = b; α = β = 90; γ = 120°	a = b; α = β; γ = 120°	-
R_{wp} (%)	5.11	3.96	8.60	7.08
R_p (%)	3.61	3.05	6.51	5.24
a (Å)	18.034(7)	17.859(7)	25.786(12)	38.06(7)
b (Å)	18.034(7)	17.859(7)	25.786(12)	32.47(3)
c (Å)	3.5058(12)	3.4764(14)	7.239(5)	3.818(2)
α (°)	90	90	81.38(3)	90
β (°)	90	90	81.38(3)	63.34(18)
γ (°)	120	120	120	90

2.2.2 Rietveld Refinements for Amine-linked COFs

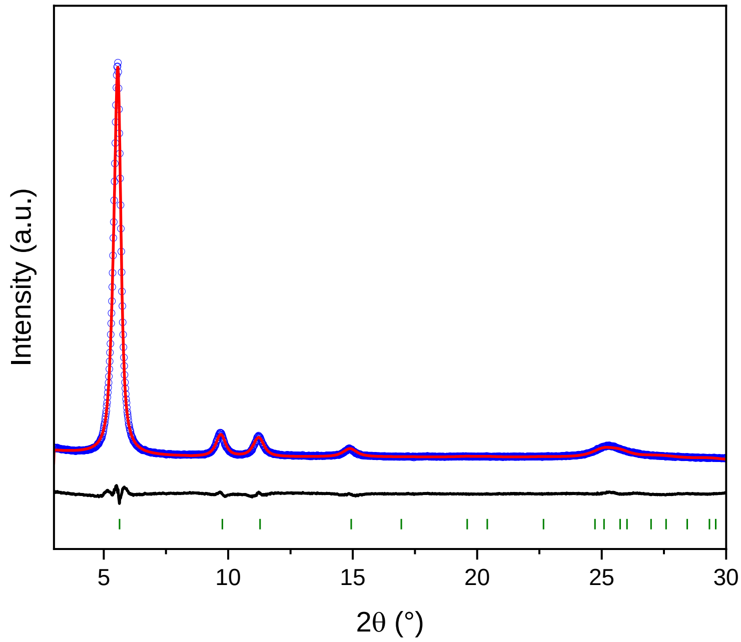

Figure S12: Experimental (blue) and simulated XRPD (Cu-Kα1) diffractogram (red) of rPI-3-COF after Rietveld refinement.

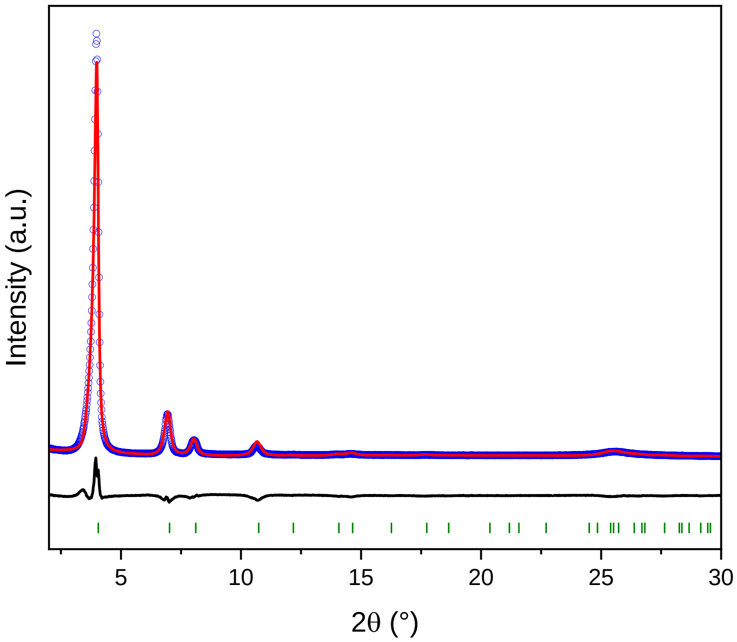

Figure S13: Experimental (blue) and simulated XRPD (Cu-Kα1) diffractogram (red) of rTTI-COF after Rietveld refinement.

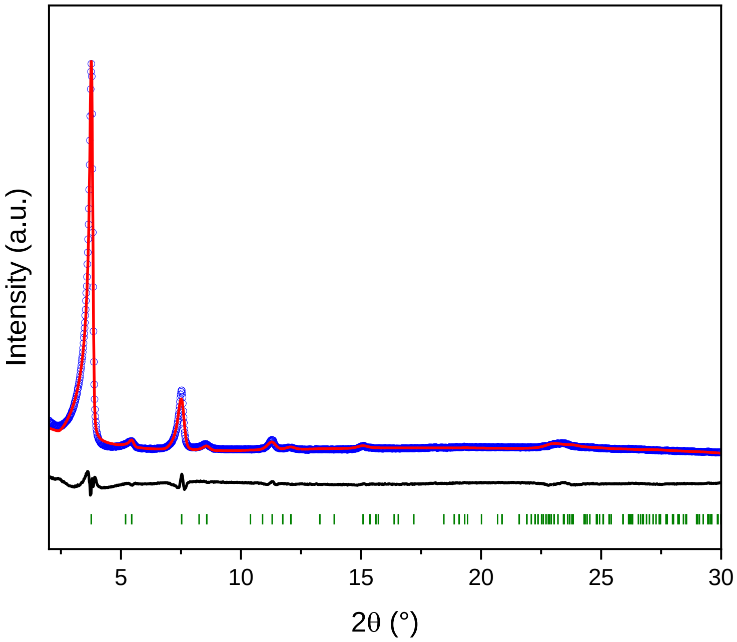

Figure S14: Experimental (blue) and simulated XRPD (Cu-Kα1) diffractogram (red) of rPy1P-COF after Rietveld refinement.

Table S2: Cell parameters of Rietveld refined amine-linked COFs. PrPy1P-COF was fitted in a constrained $P1$ space group to account for a non-symmetric occurrence of amine groups in the structure model despite the high the apparent symmetry in XRPD diffractogram.

**Fitted Pattern**	**rPI-3-COF**	**rPI-3-COF**	**rTTI-COF**	**rPy1P-COF**	**prPy1P-COF**
**Space group**	$P3$	$P\bar{6}$	$P6_3/m$	$C2/m$	$P1$
**Constraints**	-	-	-	-	α = γ = 90°
**R_{wp} (%)**	6.68	5.06	9.67	6.42	5.52
**R_p (%)**	4.83	3.75	7.53	4.88	4.11
**a (Å)**	18.087(9)	18.090(7)	25.147(9)	41.04(17)	38.34(8)
**b (Å)**	18.087(9)	18.090(7)	25.147(9)	32.43(4)	32.50(3)
**c (Å)**	3.550(2)	3.5425(12)	7.008(4)	4.069(9)	3.876(3)
**α (°)**	90	90	90	90	90
**β (°)**	90	90	90	56.0(3)	62.6(2)
**γ (°)**	120	120	120	90	90

2.2.3 Coherence Length Analysis

Table S3: Lateral and vertical crystallite coherence lengths for imine and amine linked COFs.

	**Lateral crystallite coherence length (nm)**	**Vertical crystallite coherence length (nm)**
	100/010 peak	Stacking reflection (~001)
PI-3-COF	26(2)	4(1)
rPI-3-COF	30(8)	4(2)
disordered rPI-3-COF	10(2)	2(1)
pfrPI-3-COF	8(2)	2(1)
opfrPI-3-COF	24(2)	4(1)

Coherence lengths were estimated by single line fits of the apparent 100/010 peak for the lateral direction and the apparent stacking peak observed around 25.5° 2θ for the vertical direction. For the second feature, this assumes that the peak is predominantly only from the periodicity of locally disordered layers in the stacking direction, and does not contain strong contributions from the other allowed reflections suggested by the apparent crystallographic

space group. The instrumental profile was accounted for by Rietveld refinement to a measurement of Silicon standard using constant Gaussian, Lorentzian, and exponential convolutions using TOPAS v6.[30] An additional exponential convolution was refined to the sharpest 100 peak of sample rPI-3-COF, to account for additional asymmetry in the much broader diffraction patterns observed for the COFs that could not be accounted for with the standard profile. This was not found to have a large impact on the crystallite sizes obtained from the refinement of this sample, and was fixed for the other samples for comparison. Then, peak broadening from the samples was accounted for by an additional Lorentzian broadening term, and the crystallite sizes in the respective directions were estimated from the resulting volume averaged column heights (LVol-IB). The errors were estimated as the refinement uncertainties multiplied by a factor of ten. It is important to note that these values roughly estimate the minimum crystallite coherence lengths of the respective directions, and can result from either discrete crystallites or the buildup of defects resulting in an uncorrelated relationship between atoms at longer distances.

The values are in excellent agreement with the measured PDF data, i.e. that the lateral coherence of the disordered samples is significantly smaller than the ordered samples. Also, the coherence of the stacked layers is much smaller in all samples, likely due to random local layer offsets. The stacking coherence of the samples appears slightly longer from the PDF data, about 4 nm for disordered samples and 6 nm for ordered samples. It is further worth noting that, by the relative peak intensities, disordered rPI-3-COF appears to have relatively more content with ordered lateral dimensions while pfrPI-3-COF appears to have relatively more content with ordered stacking.

2.2.4 Additional XRPD Patterns

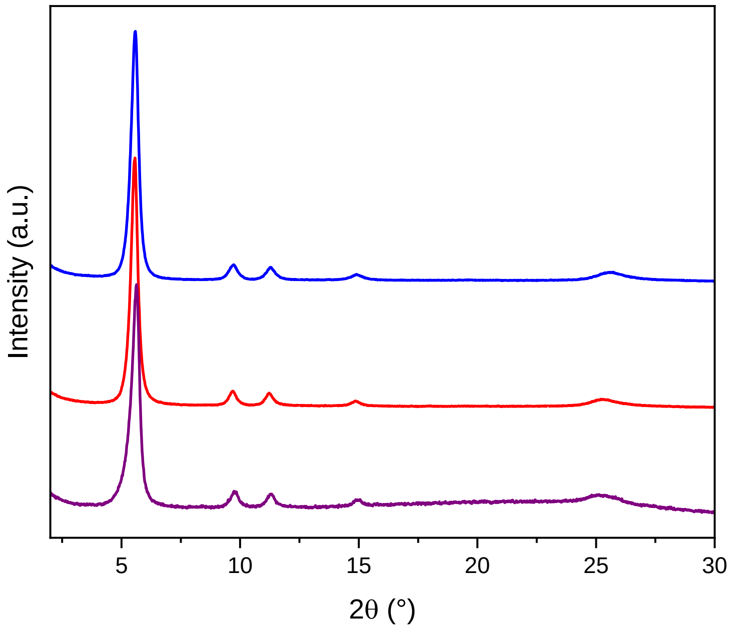

Figure S15: XRPD (Cu-Kα1) patterns of PI-3-COF (blue) and rPI-3-COF obtained by reduction of PI-3-COF (red) in comparison to rPI-3-COF obtained by one-pot crystallization/reduction approach.

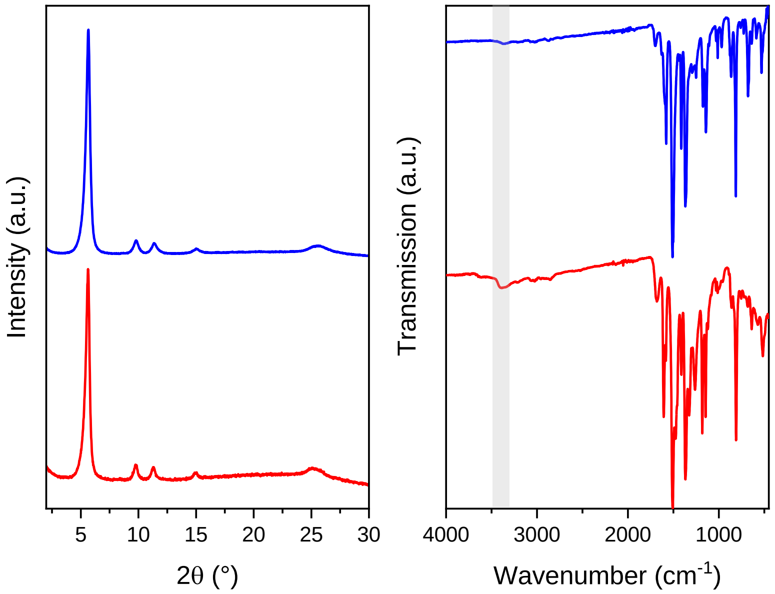

Figure S16: XRPD (Cu-Kα1) patterns and FT-IR spectra of one-pot synthesized rPI-3 COF at 60°C (after 2 days, blue line) and 120°C (after 3d, red line). Grey area highlights secondary amine vibrations caused by reduction of imine linkages. A comparison of both reaction temperatures shows that formic acid catalyzes the crystallization process already at 60°C, while higher temperatures are needed for fast reduction of the framework.

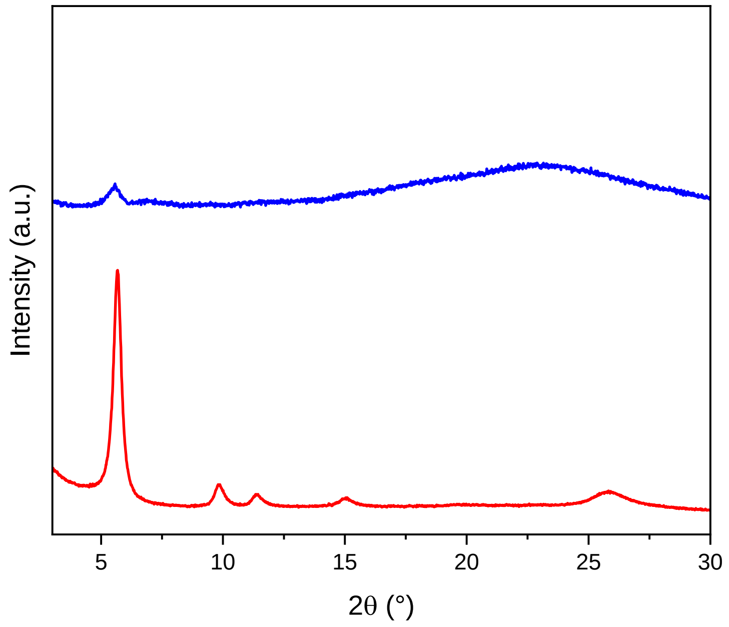

Figure S17: XRPD (Cu-Kα1) patterns of pfrPI-3-COF (blue) and opfrPI-3-COF (red) - same sample after oxidation with DDQ. Increased crystallinity after oxidation supports the hypothesis that disorder in pfrPI-3-COF is influenced by structural disorder, while the molecular structure of the framework is kept intact.

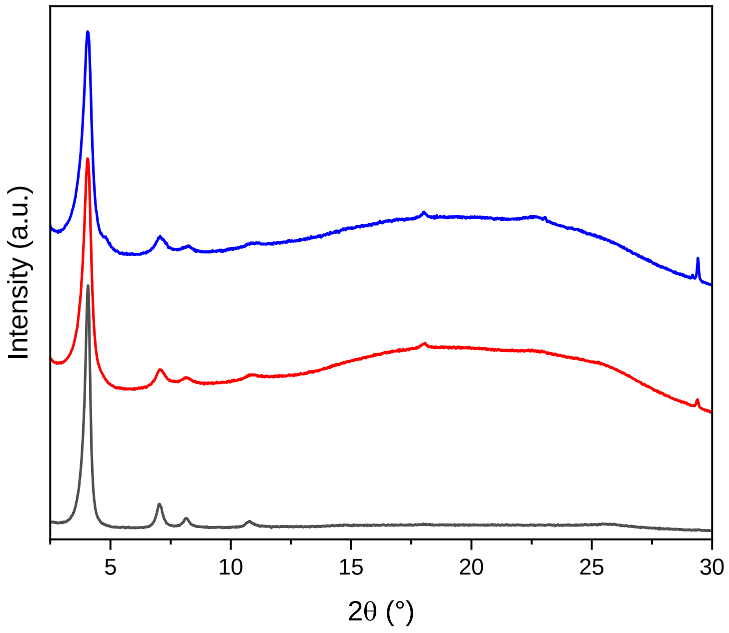

Figure S18: XRPD (Cu-Kα1) pattern comparison of BzCl-rTTI-COF (blue), TDI-rTTI-COF (red) and rTTI-COF (black). Increased background and broadened, but defined reflections indicate that the structure of the functionalized COFs is principally retained, but may also be influenced by steric interactions of the immobilized moieties.

2.3 ssNMR Spectroscopy

2.3.1 ssNMR Spectra of Imine-linked COFs

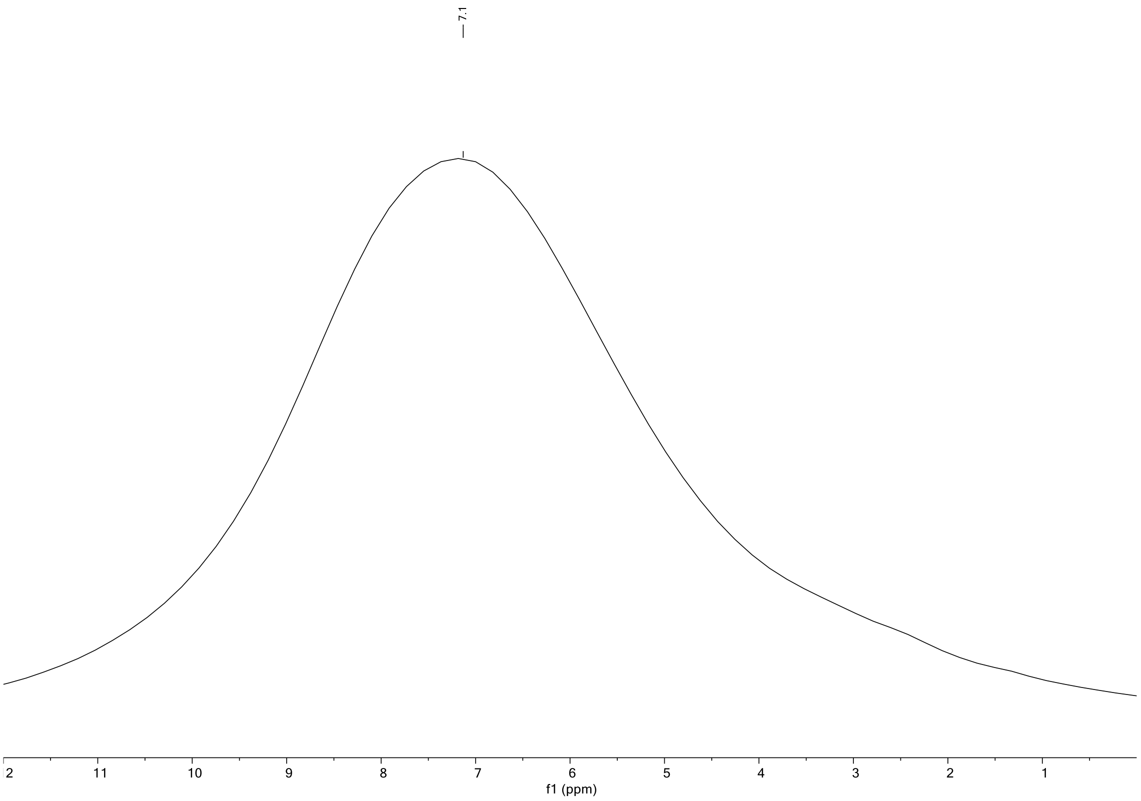

Figure S19: ^{1}H ssNMR MAS spectrum of PI-3-COF.

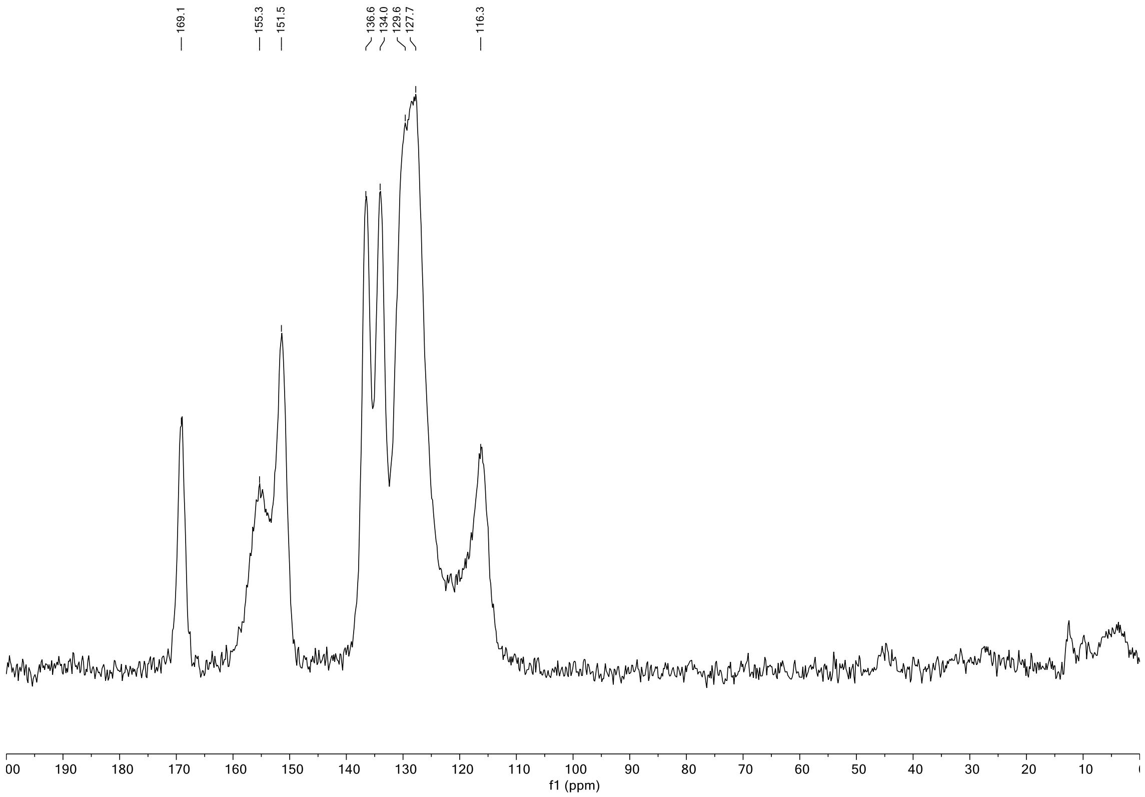

Figure S20: ^{13}C ssNMR CP-MAS spectrum of PI-3-COF.

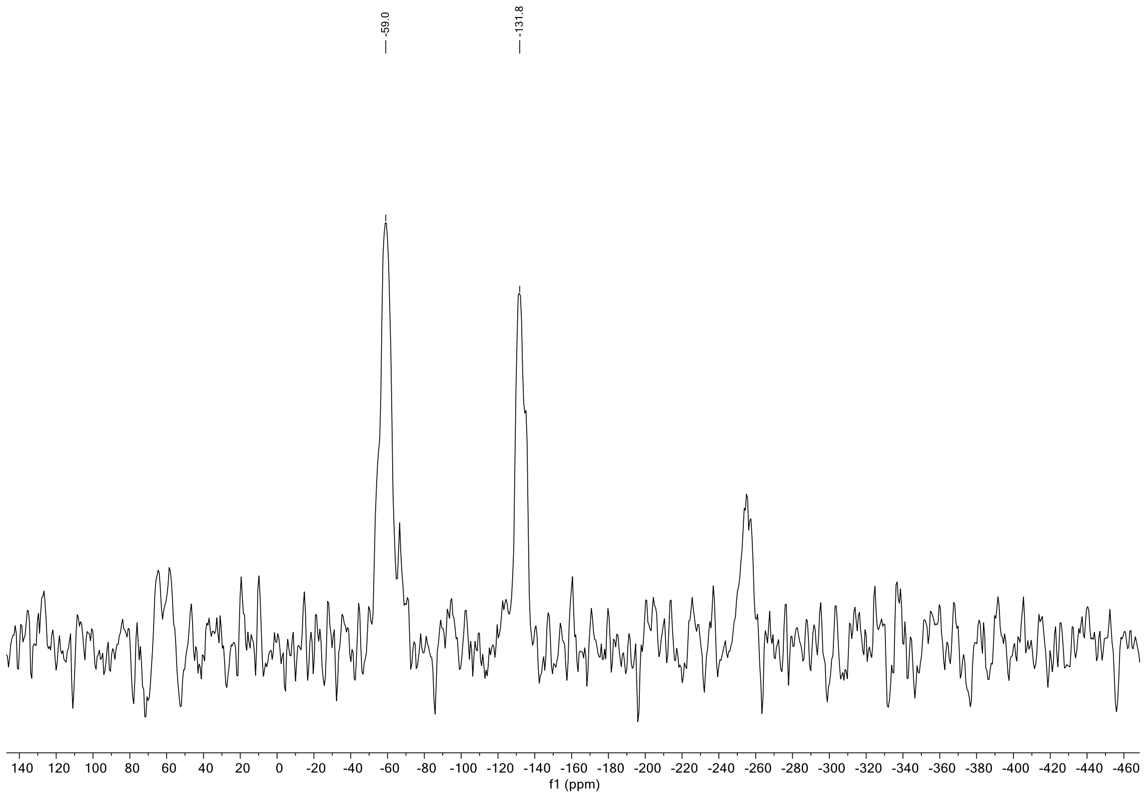

Figure S21: ^{15}N ssNMR CP-MAS spectrum of PI-3-COF.

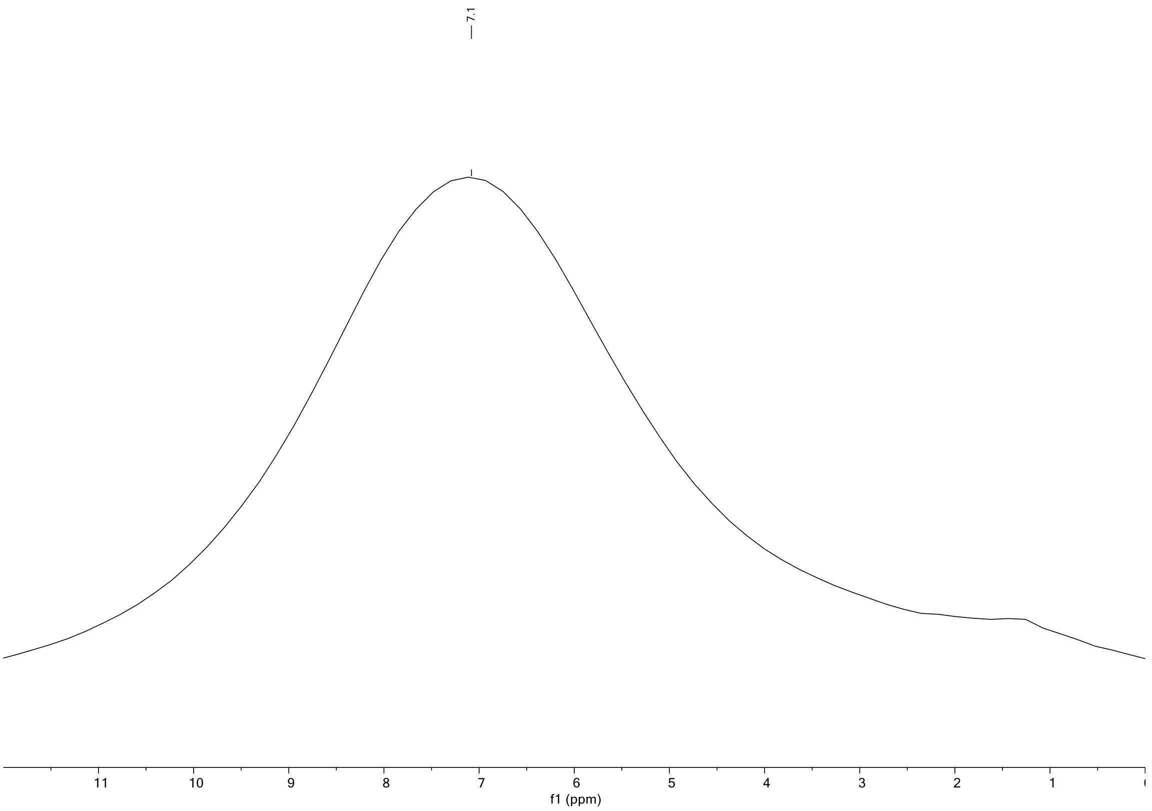

Figure S22: ^{1}H ssNMR MAS spectrum of TTI-COF.

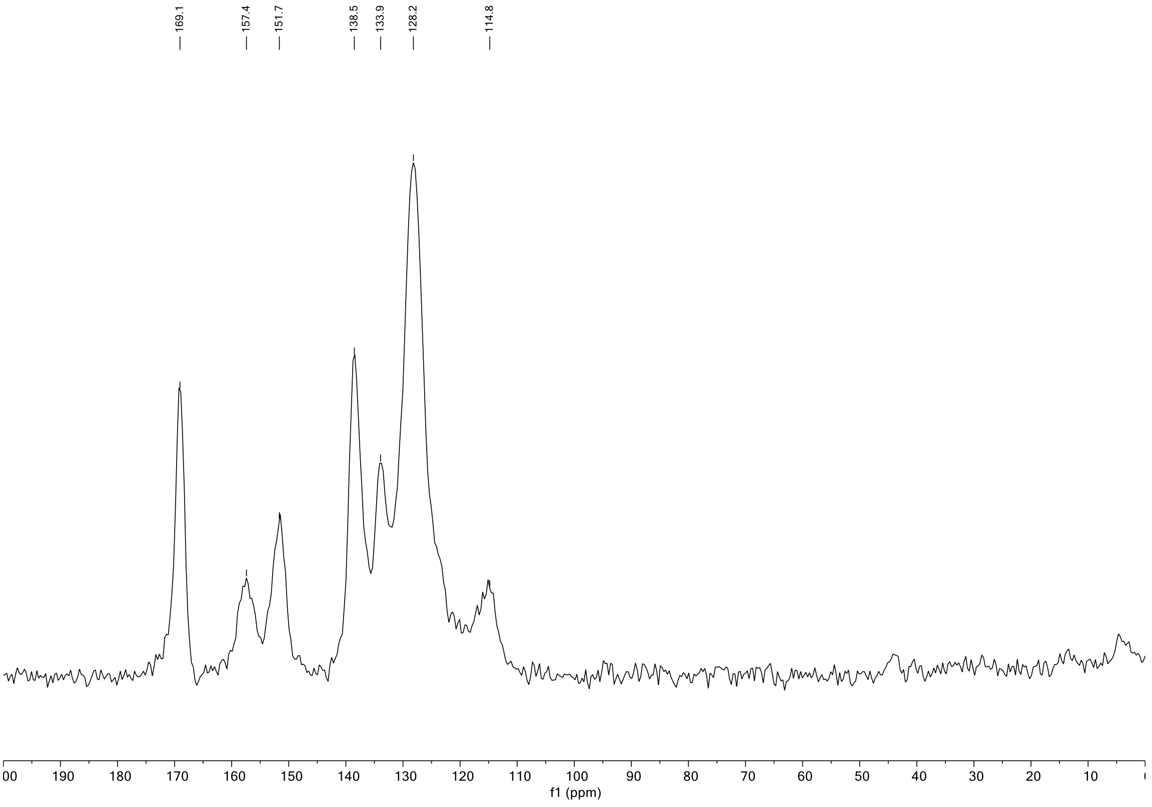

Figure S23: ^{13}C ssNMR CP-MAS spectrum of TTI-COF.

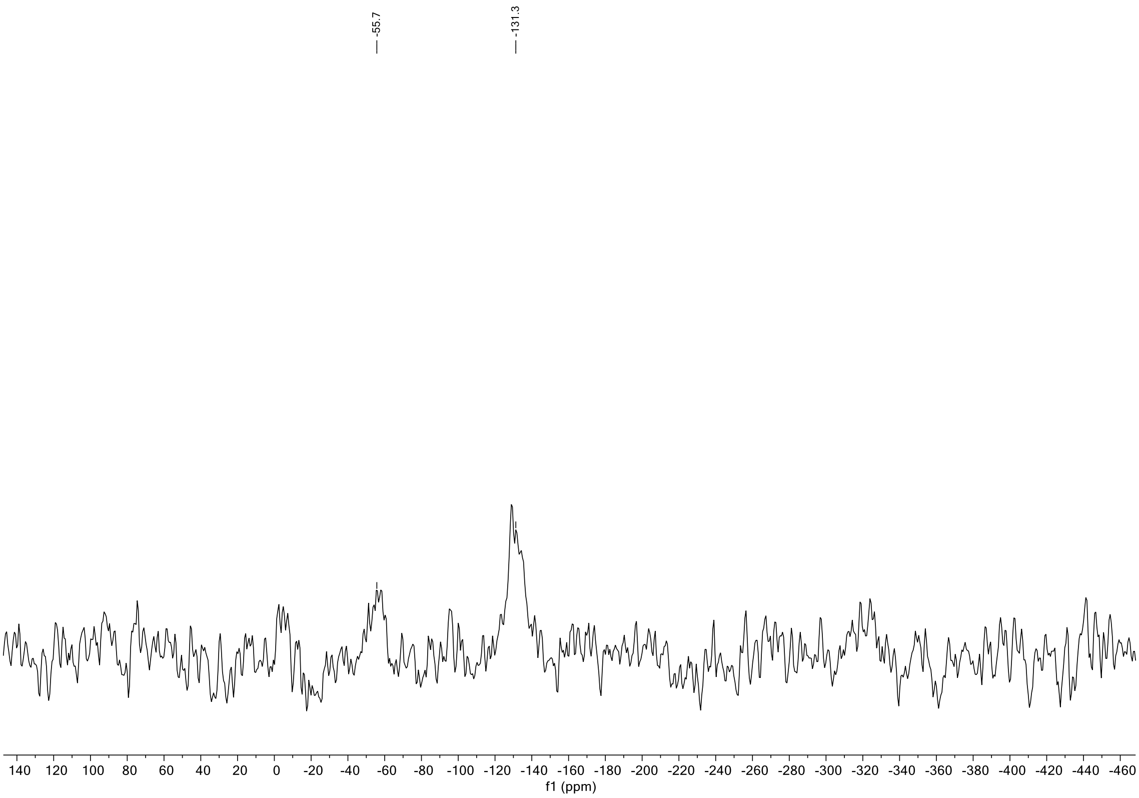

Figure S24: ^{15}N ssNMR CP-MAS spectrum of TTI-COF.

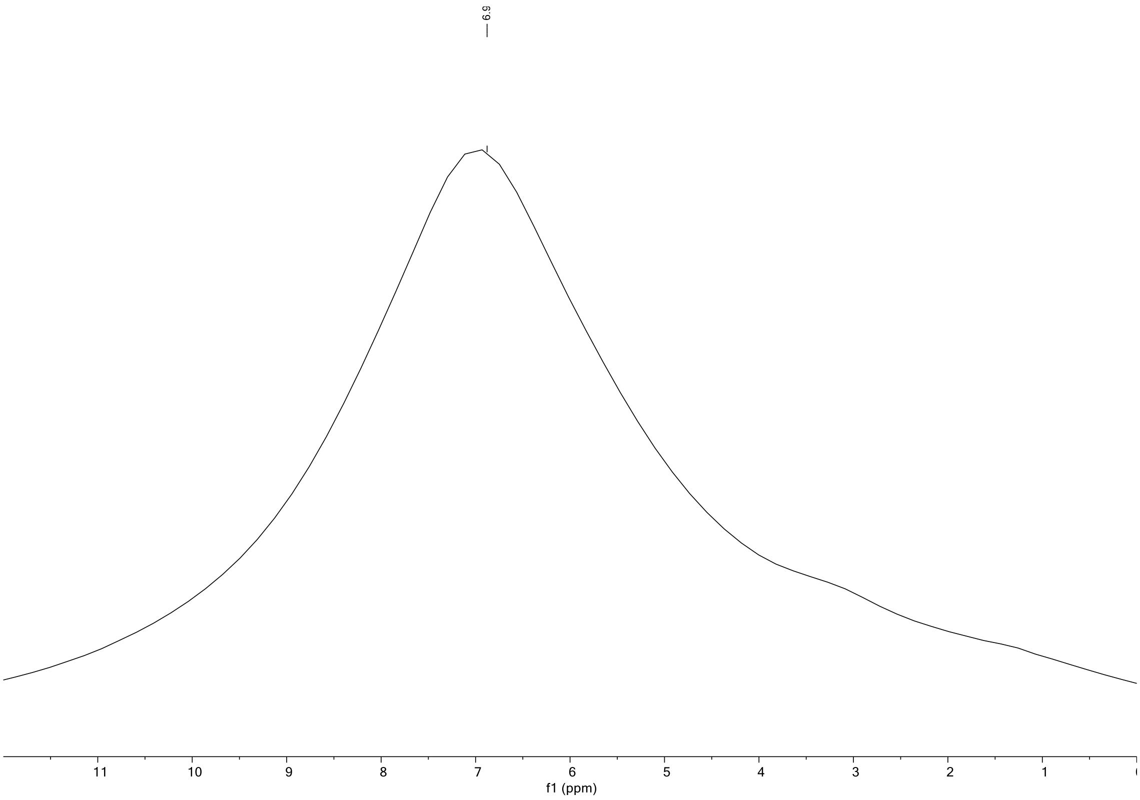

Figure S25: ^{1}H ssNMR MAS spectrum of Py1P-COF.

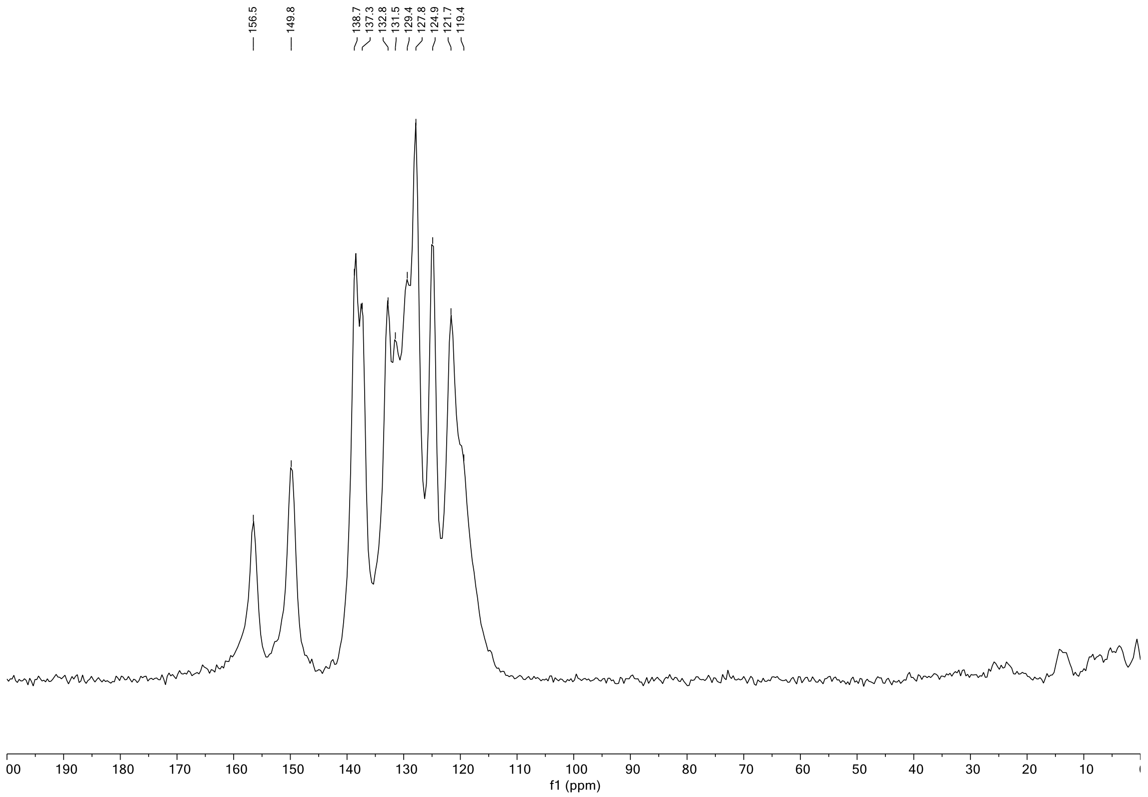

Figure S26: ^{13}C ssNMR CP-MAS spectrum of Py1P-COF.

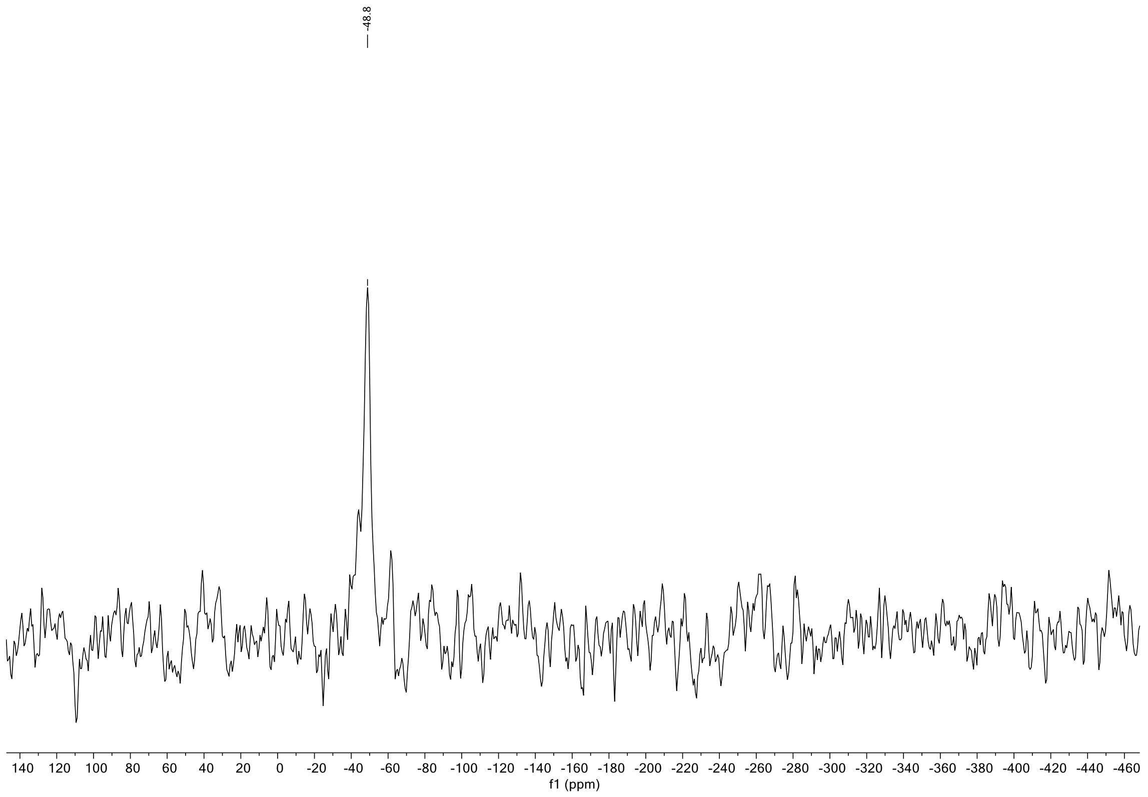

Figure S27: ^{15}N ssNMR CP-MAS spectrum of Py1P-COF.

2.3.2 ssNMR Spectra of Amine-linked COFs

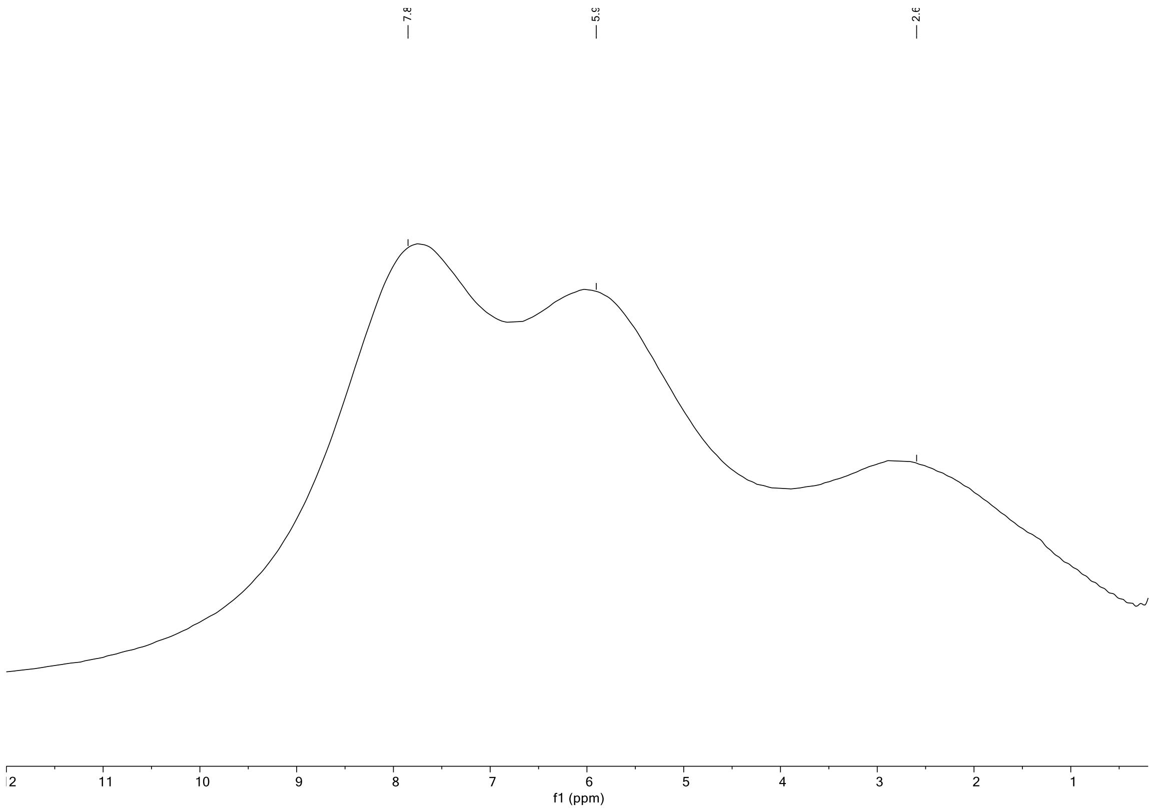

Figure S28: ^{1}H ssNMR MAS spectrum of rPI-3-COF.

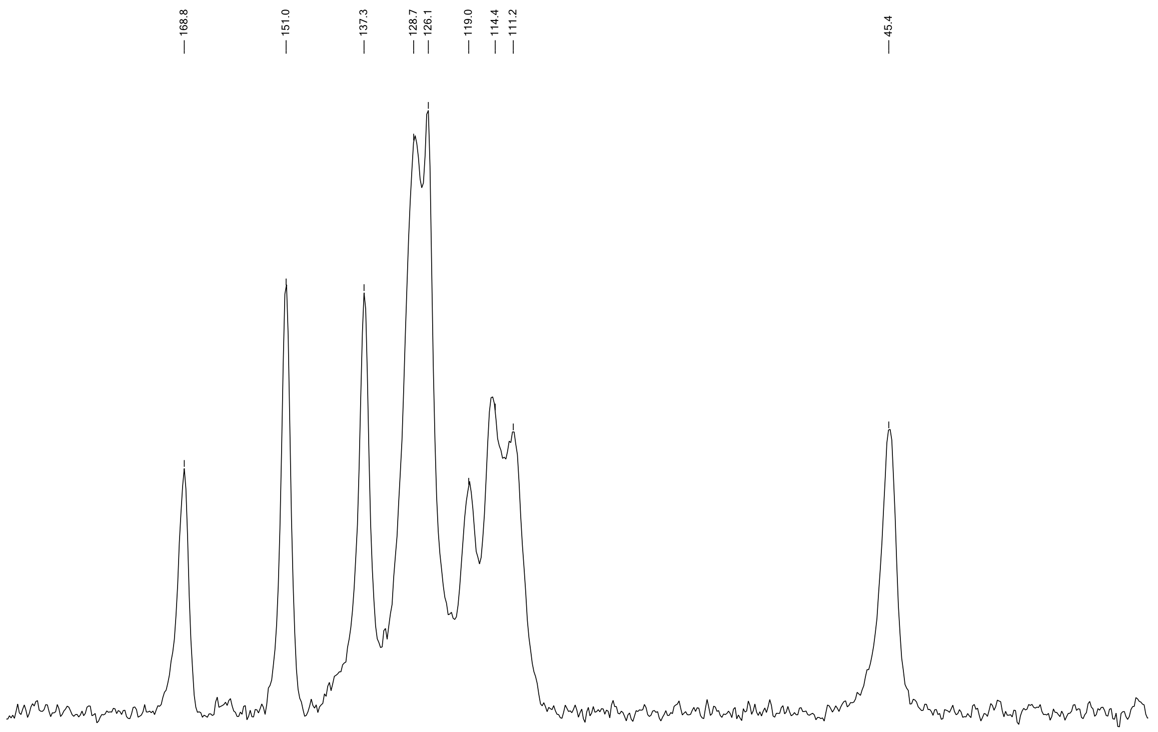

Figure S29: ^{13}C ssNMR CP-MAS spectrum of rPI-3-COF.

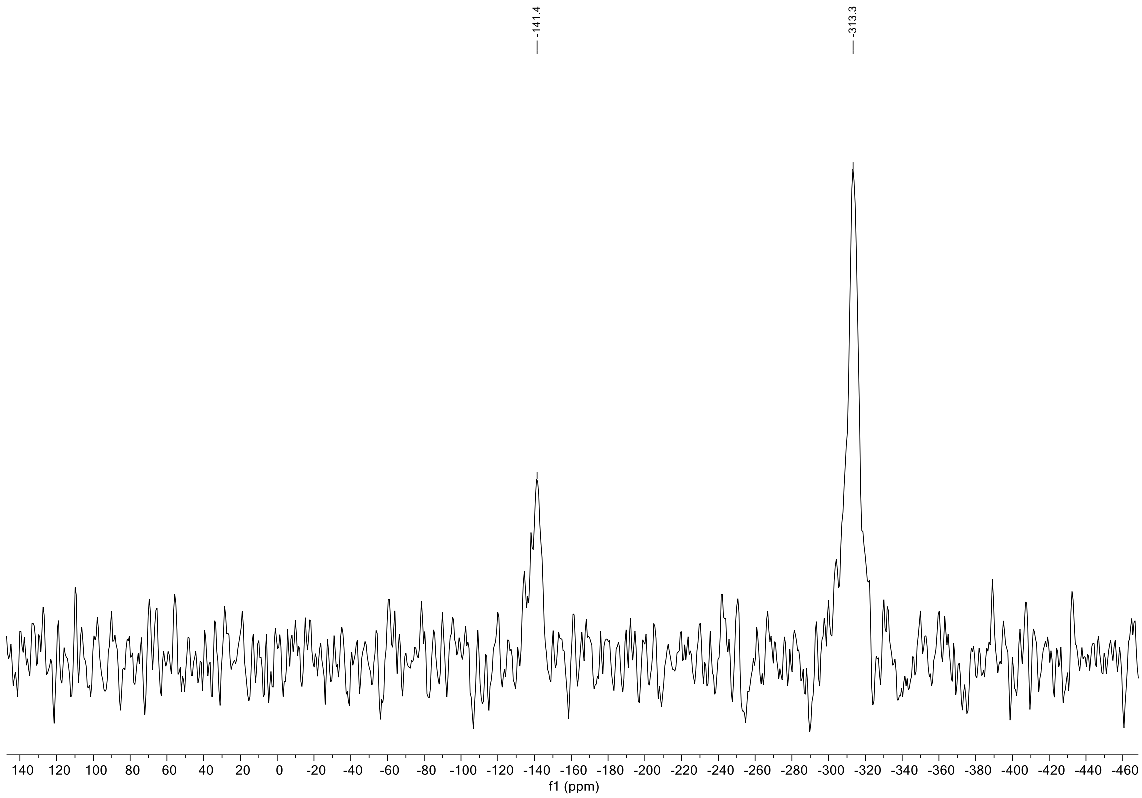

Figure S30: ^{15}N ssNMR CP-MAS spectrum of rPI-3-COF.

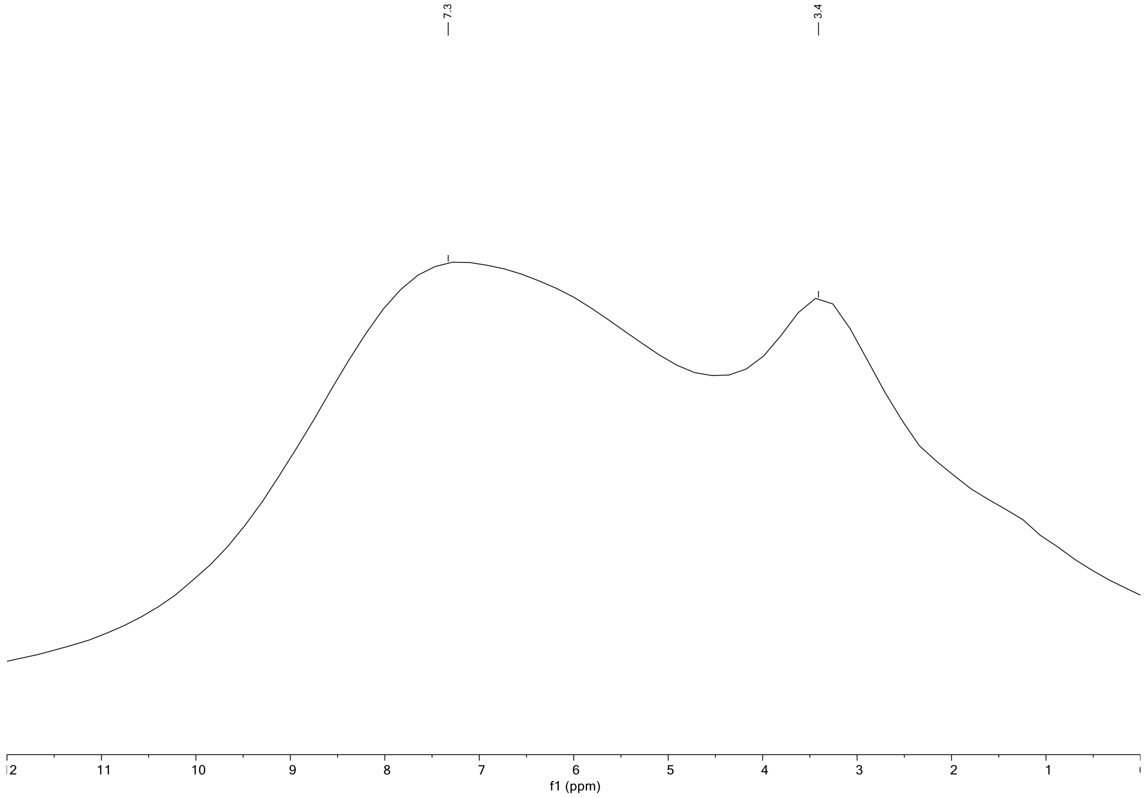

Figure S31: ^{1}H ssNMR MAS spectrum of disordered rPI-3-COF.

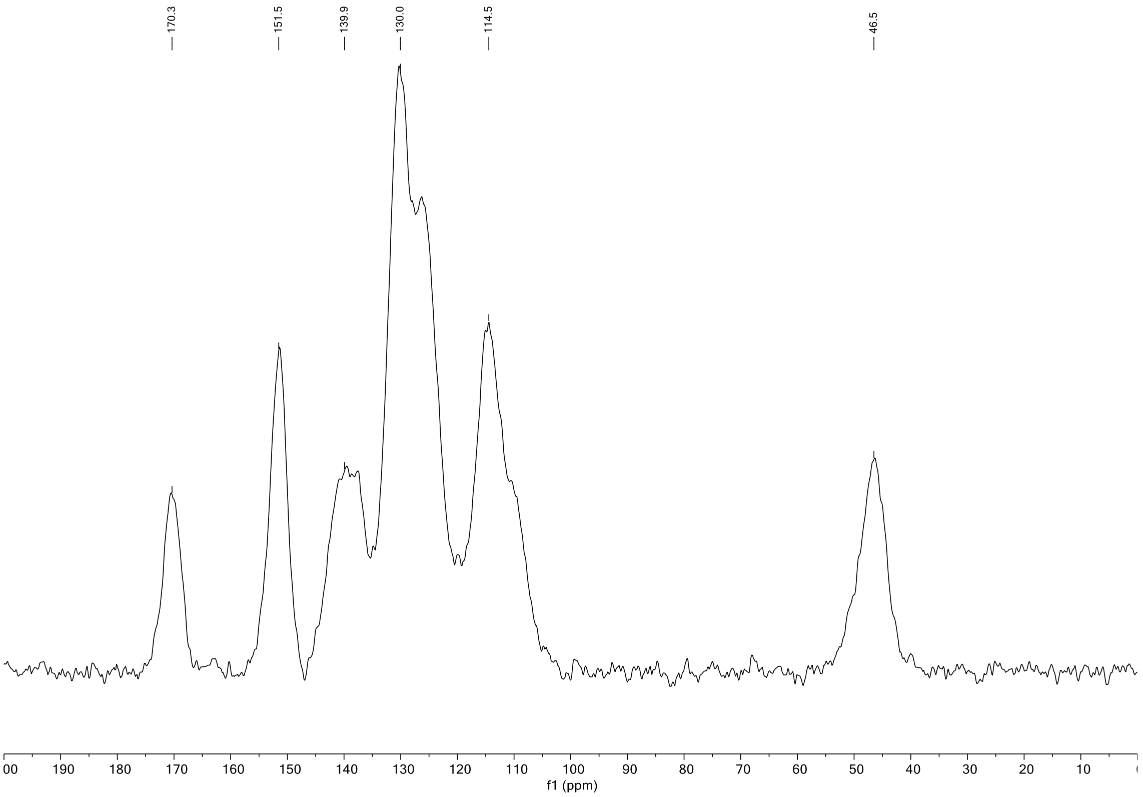

Figure S32: ^{13}C ssNMR CP-MAS spectrum of disordered rPI-3-COF.

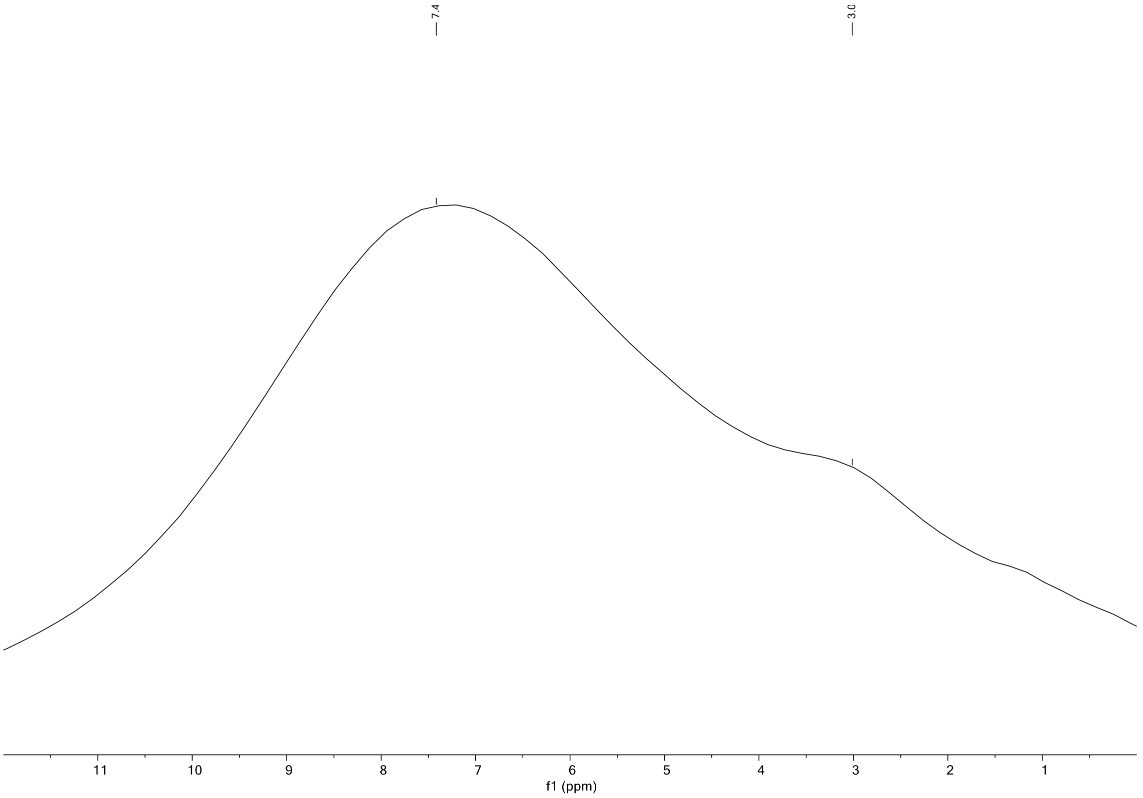

Figure S33: ^{1}H ssNMR MAS spectrum of rTTI-COF.

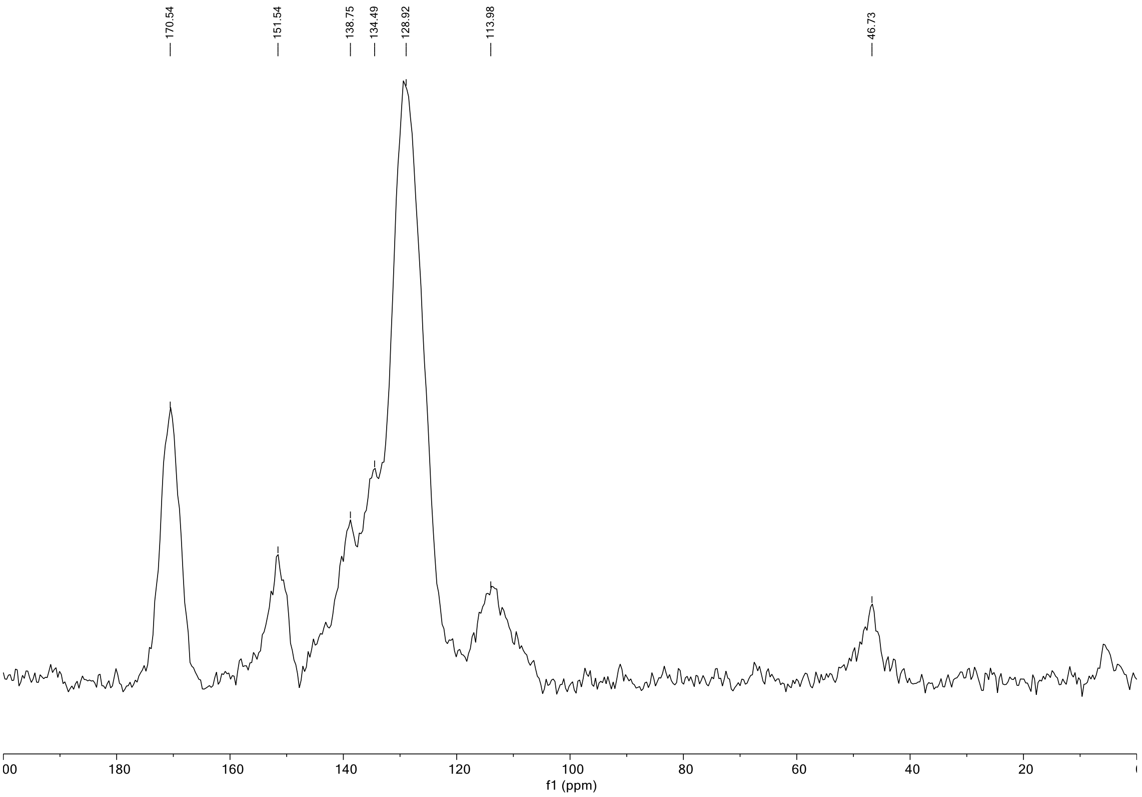

Figure S34: ^{13}C ssNMR CP-MAS spectrum of rTTI-COF.

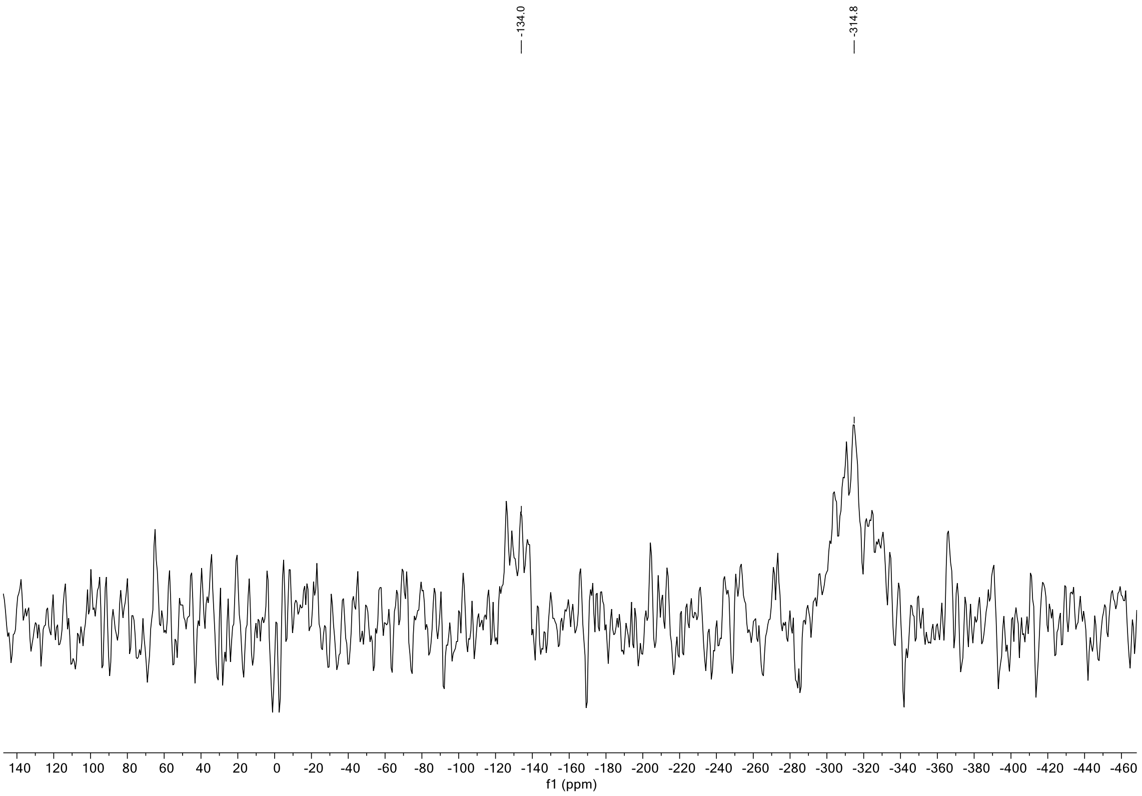

Figure S35: ^{15}N ssNMR CP-MAS spectrum of rTTI-COF.

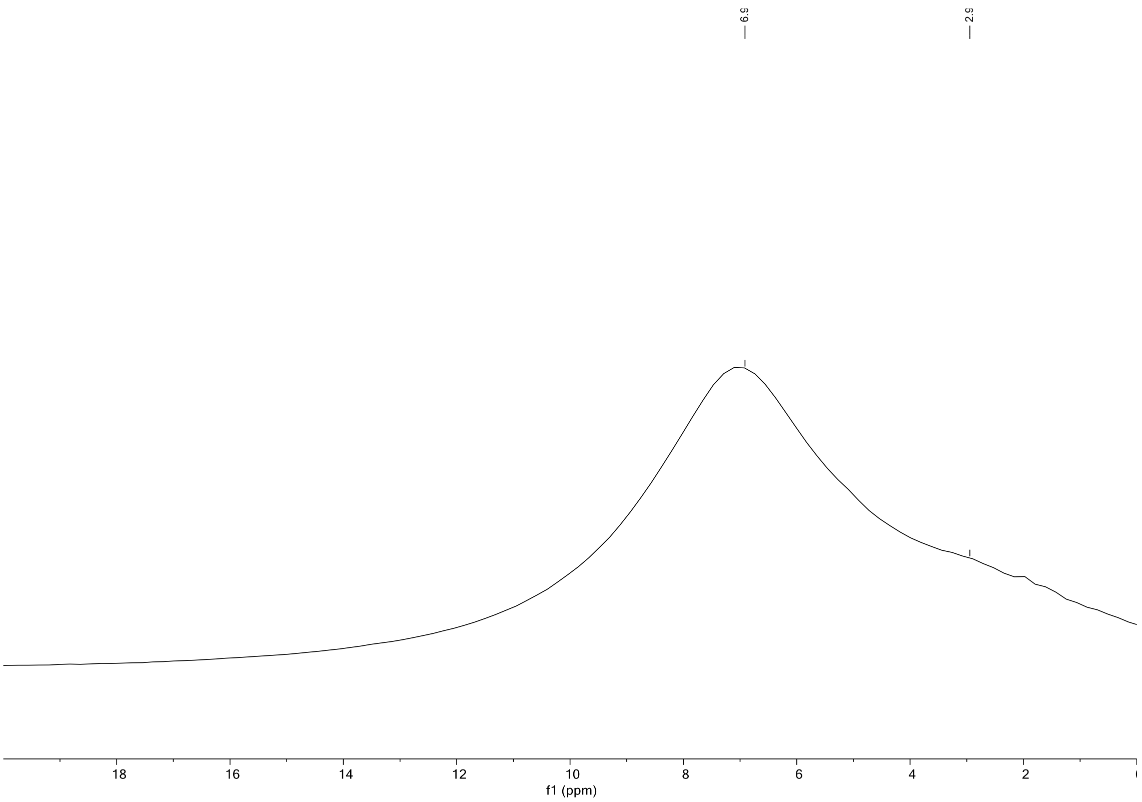

Figure S36: ^{1}H ssNMR MAS spectrum of rPy1P-COF.

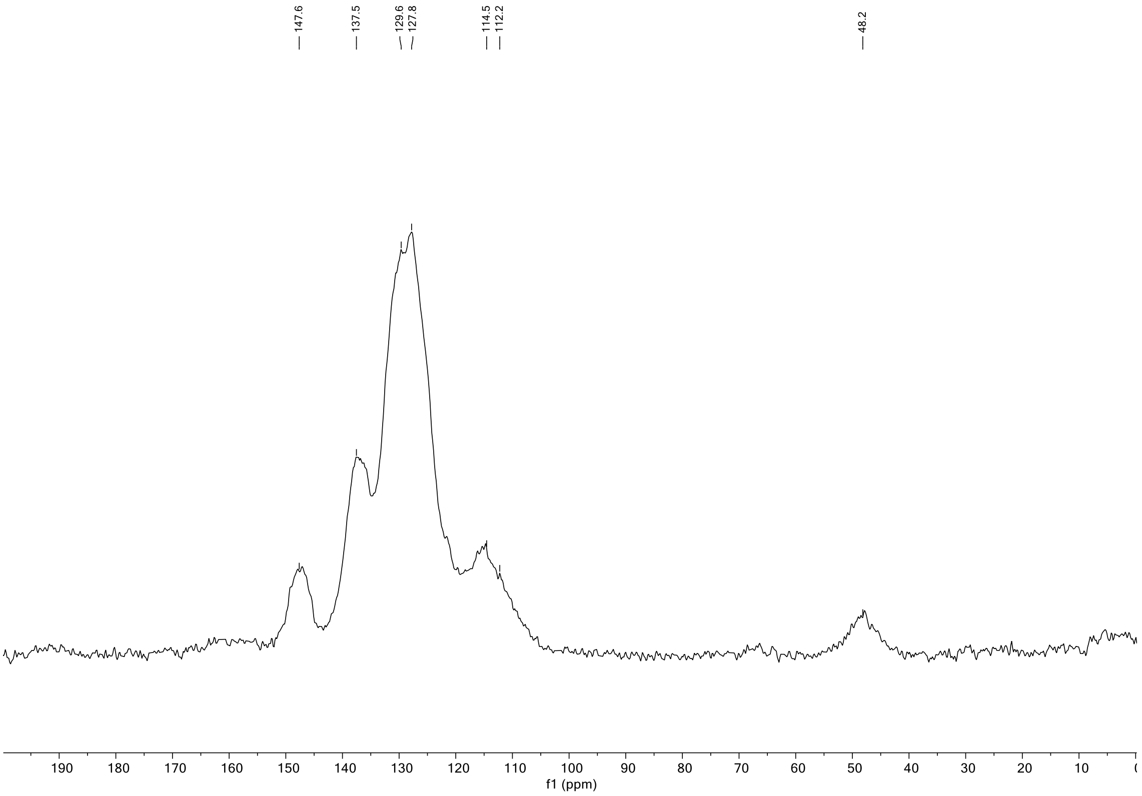

Figure S37: ^{13}C ssNMR CP-MAS spectrum of rPy1P-COF.

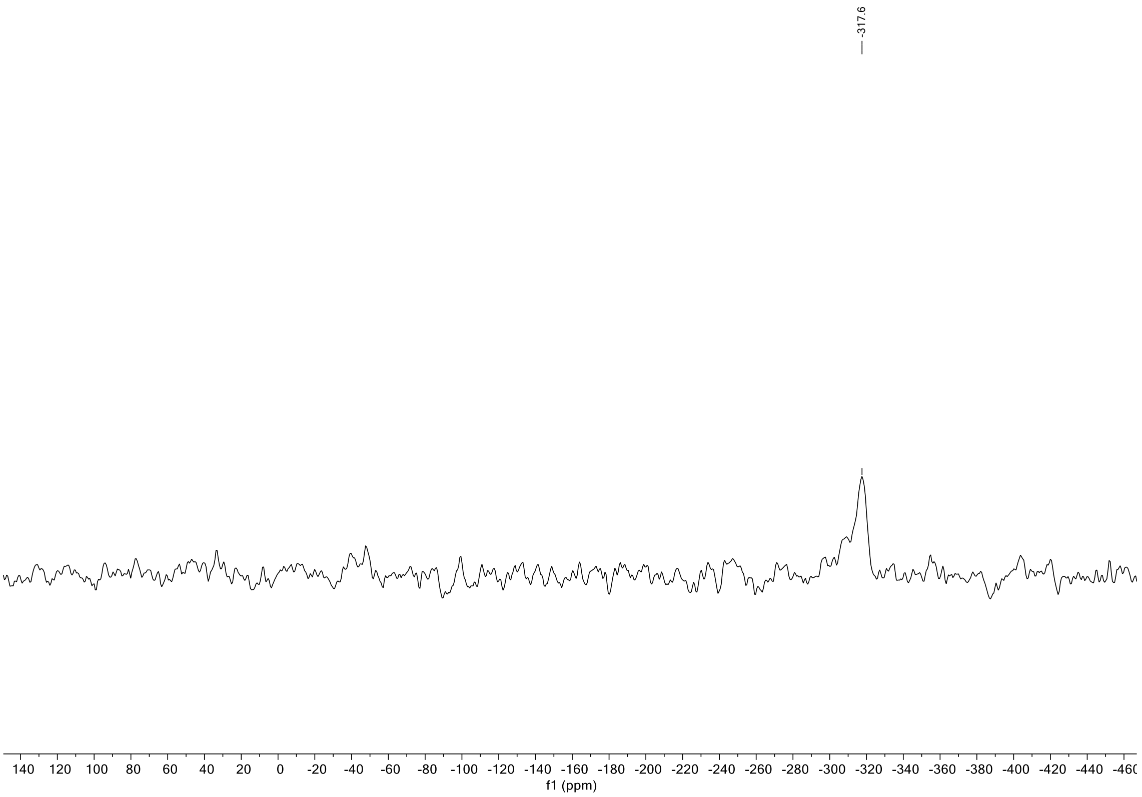

Figure S38: ^{15}N ssNMR CP-MAS spectrum of rPy1P-COF.

2.3.3 ssNMR Spectra of Hybrid Materials

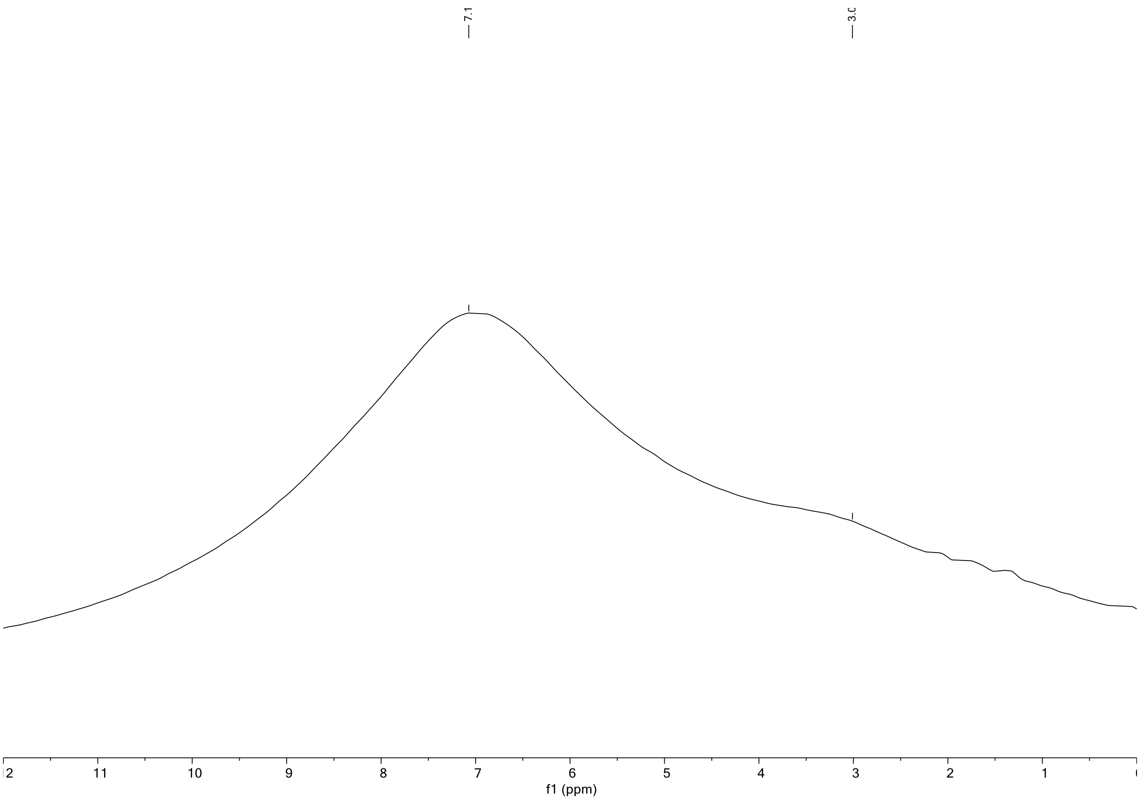

Figure S39: ^{1}H ssNMR MAS spectrum of prPy1P-COF.

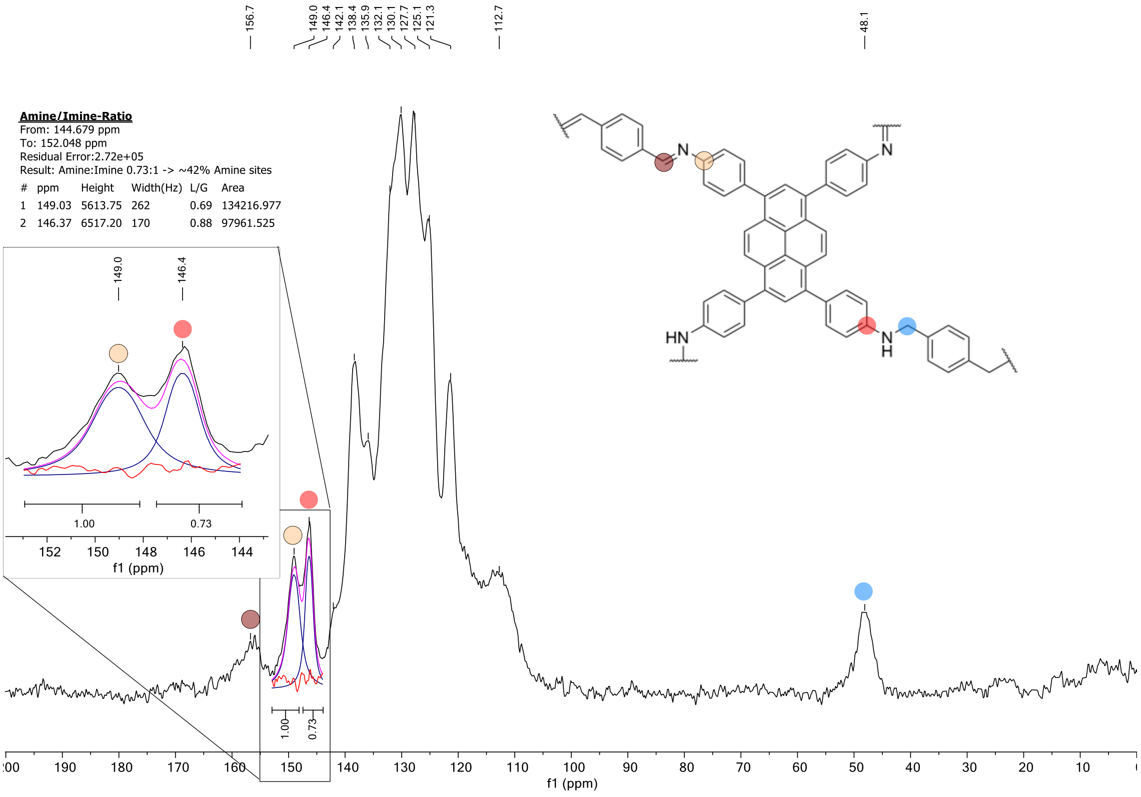

Figure S40: ^{13}C ssNMR CP-MAS spectrum of prPy1P-COF including line fitting to determine amine/imine ratio. Note that this approximation can only be done from this CP experiment, because relaxation times of the quaternary carbons (orange, red) are expected to be similar.

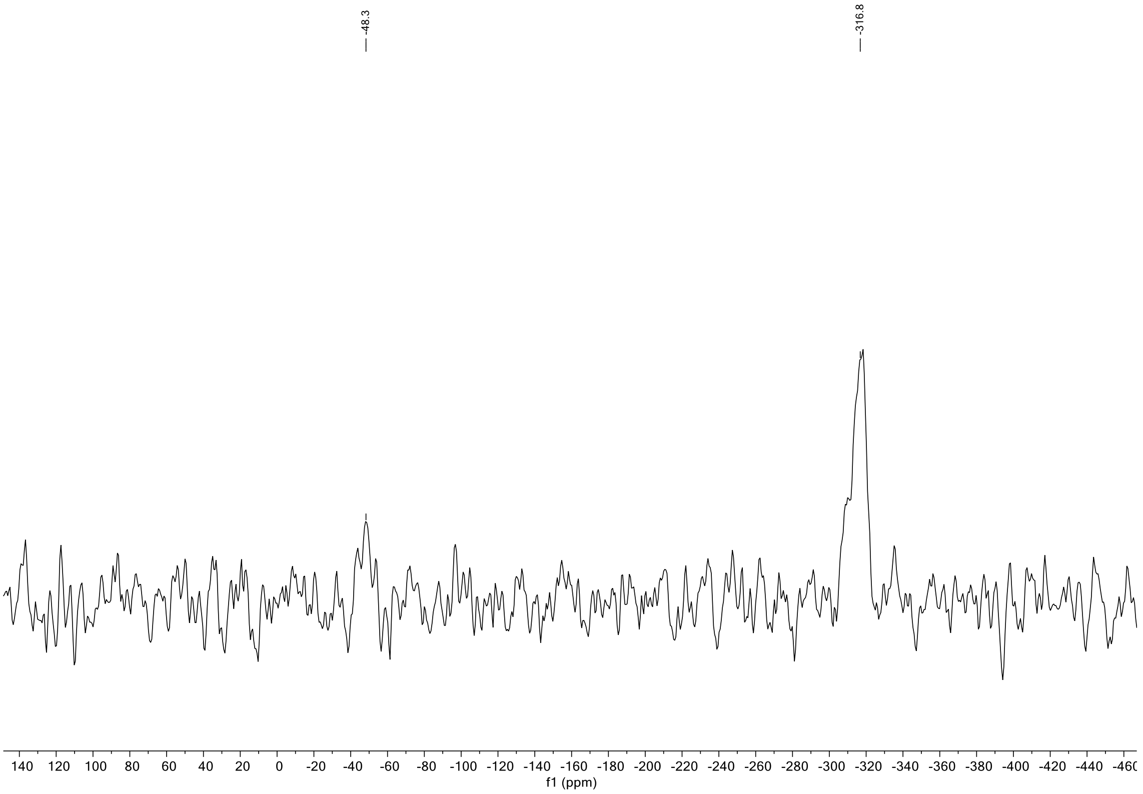

Figure S41: ^{15}N ssNMR CP-MAS spectrum of prPy1P-COF

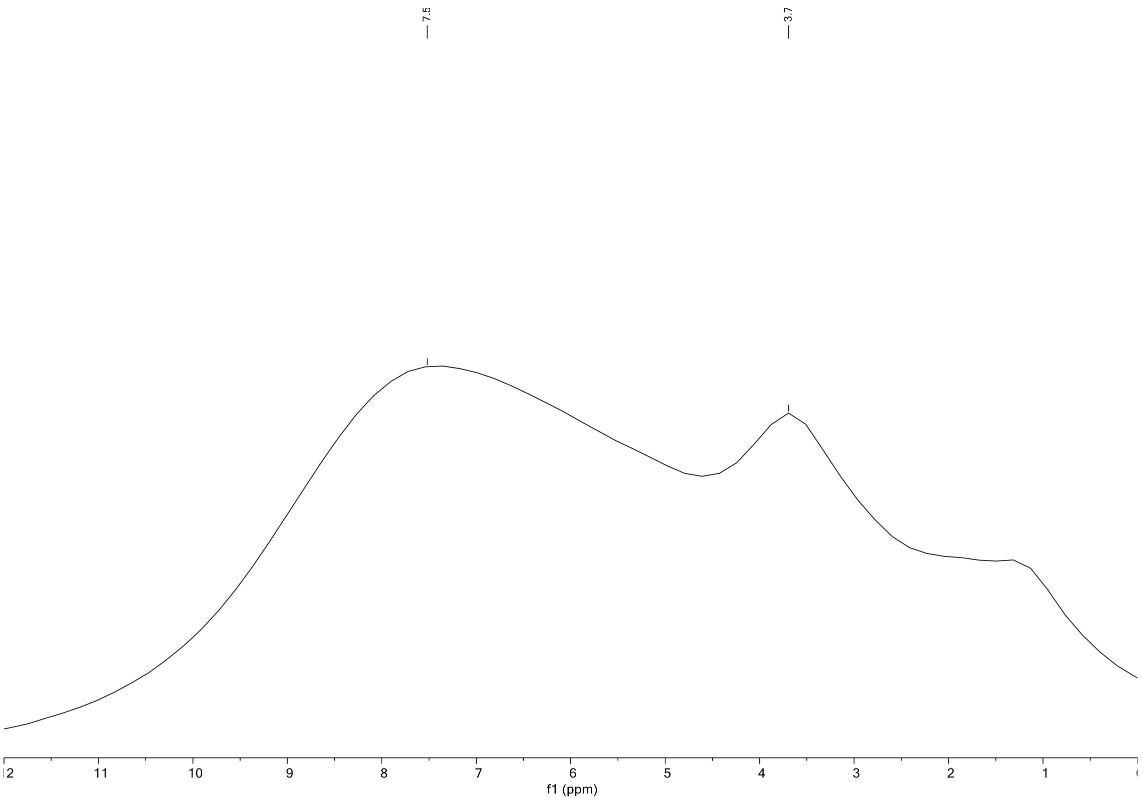

Figure S42: ^{1}H ssNMR MAS spectrum of pfrPI-3-COF.

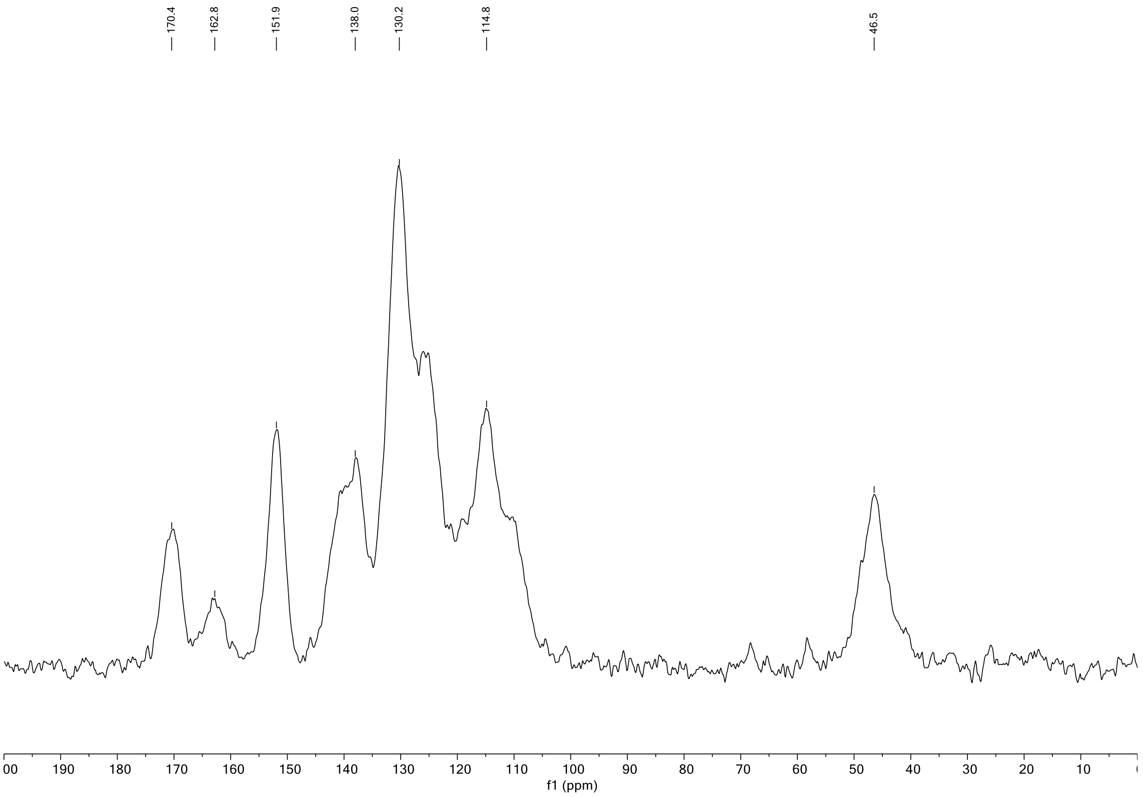

Figure S43: ^{13}C ssNMR MAS spectrum of pfrPI-3-COF.

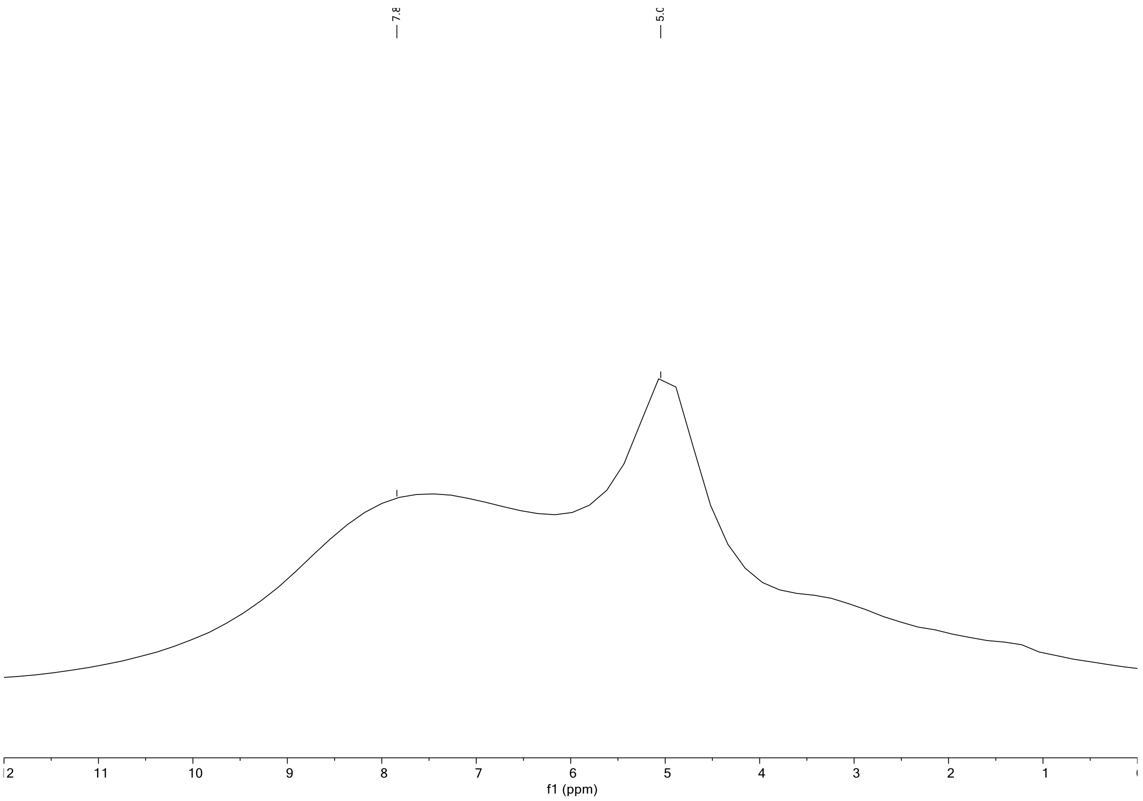

Figure S44: ^{1}H ssNMR MAS spectrum of opfrPI-3-COF.

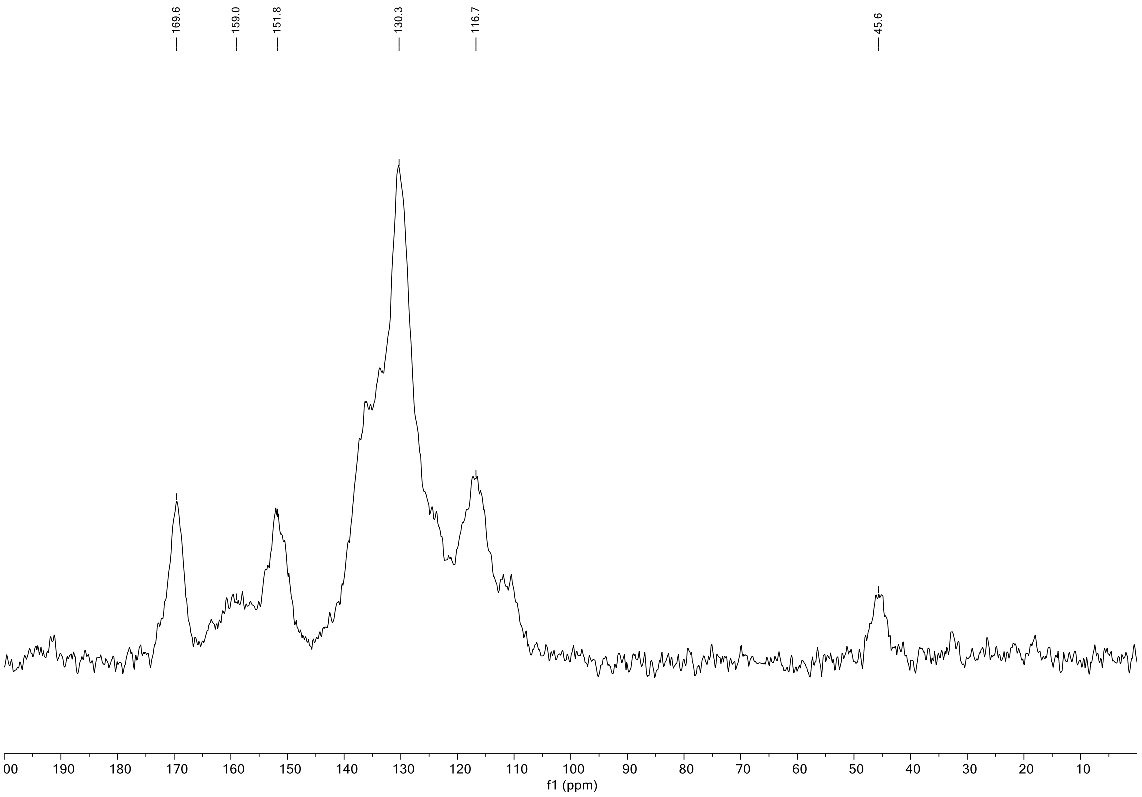

Figure S45: ^{13}C ssNMR MAS spectrum of opfrPI-3-COF.

2.3.4 Additional ssNMR Spectra

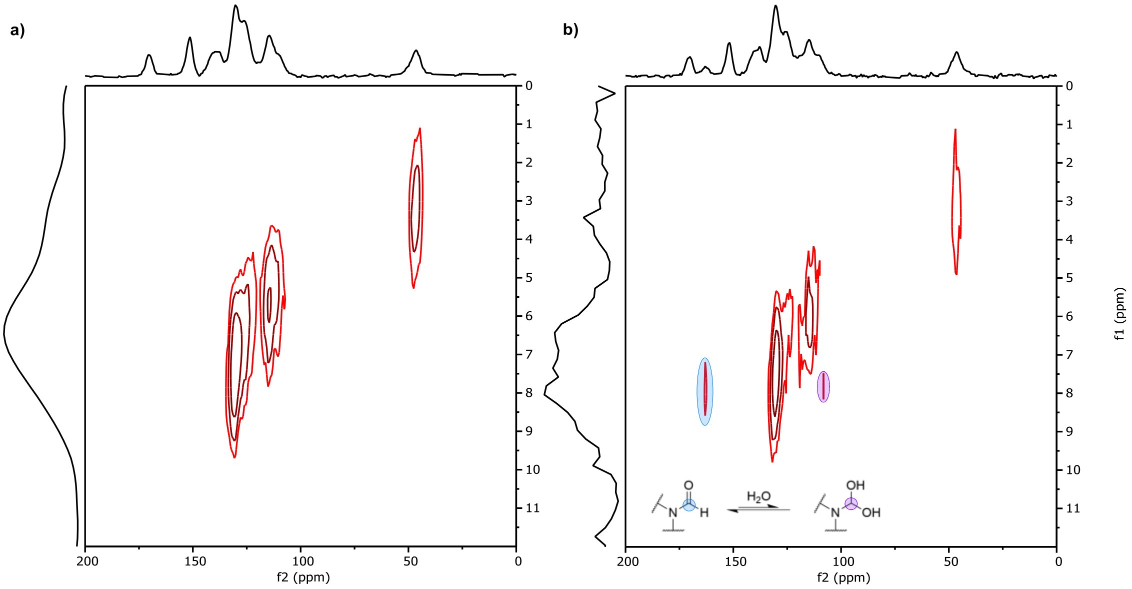

Figure S46: ^{1}H/^{13}C-HETCOR ssNMR spectra of disordered rPI-3-COF (a) and pfrPI-3-COF (b) in comparison. The spectra are plotted against ^{13}C-CP MAS spectra (horizontal trace) and internal projection (vertical trace). N-Formyl groups (blue) form hydrates (purple) with residual pore water, bound as hydrate in pfrPI-3-COF, resulting in an additional but weak signal at 108 ppm in the carbon spectrum. After treatment with aqueous acid, the signals disappear, suggesting a deprotection of the secondary amines under these conditions – affording disordered rPI-3-COF (a).

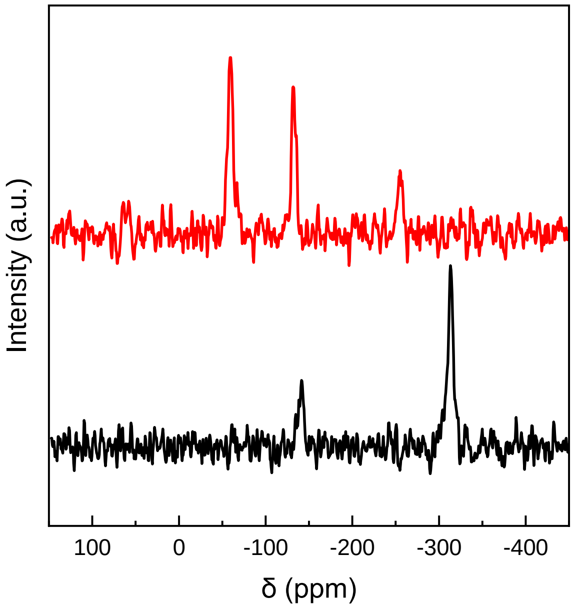

Figure S47: Comparison of ^{15}N-CPMAS ssNMR spectra of PI-3-COF (red) and rPI-3-COF (black).

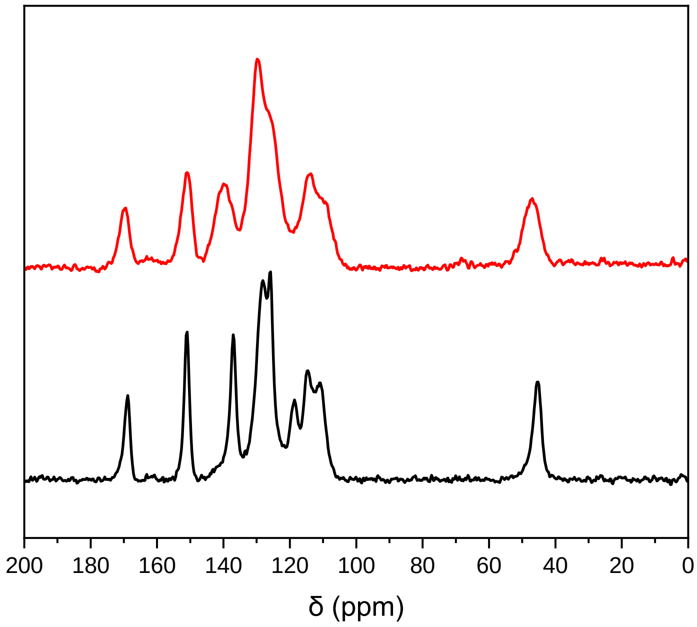

Figure S48: Comparison of ^{13}C-CPMAS ssNMR spectra of disordered rPI-3-COF (red) and rPI-3-COF (black). Broadened signals indicate disorder in the framework. Note that signal at 119 ppm refers to planar orientation of phenyl rings in the crystalline, well ordered material.

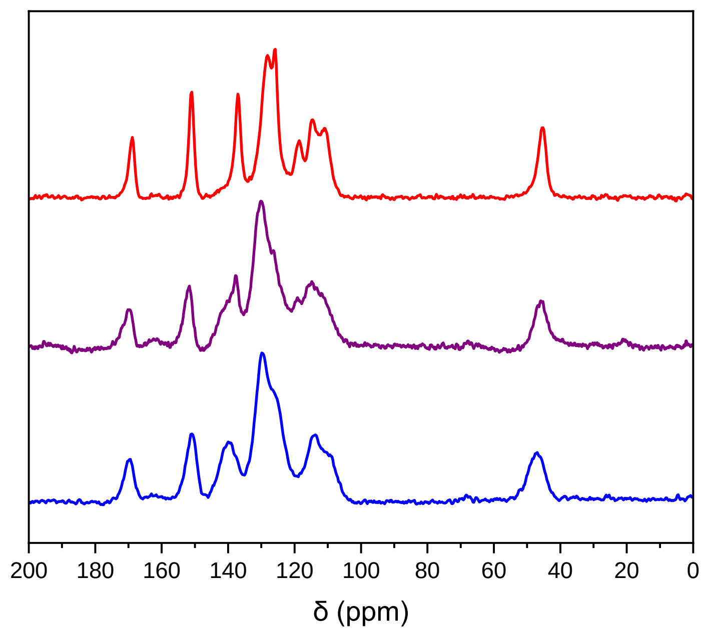

Figure S49: Comparison of ^{13}C-CPMAS ssNMR spectra of rPI-3-COF obtained by reduction of PI-3-COF (red), rPI-3-COF obtained by one-pot reductive crystallization (purple) and disordered rPI-3-COF (blue).

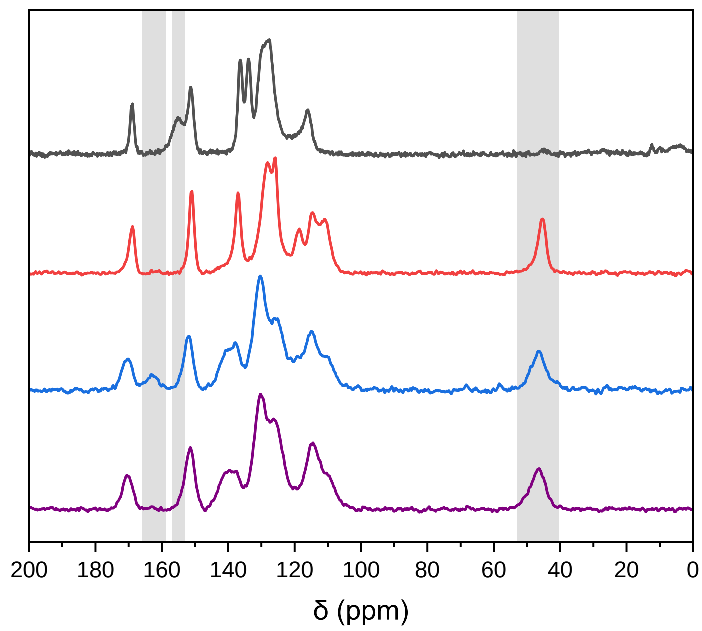

Figure S50: Comparison of ^{13}C-CPMAS ssNMR spectra of PI-3-COF (black), rPI-3-COF (red), pfrPI-3-COF (blue) and disordered rPI-3-COF (purple). Grey areas indicate N-formyl, imine, and secondary amine carbons, respectively.

2.4 N_2 Adsorption Data

2.4.1 Sorption Isotherms of Imine-linked COFs

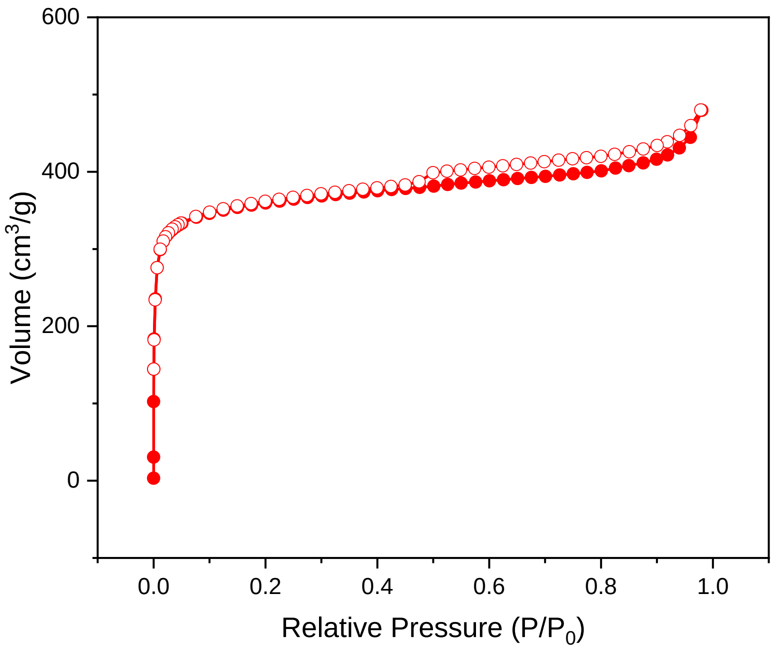

Figure S51: N_2 sorption isotherm of PI-3-COF. Filled dots represent data points of the adsorption branch, hollow dots those of the desorption branch, respectively.

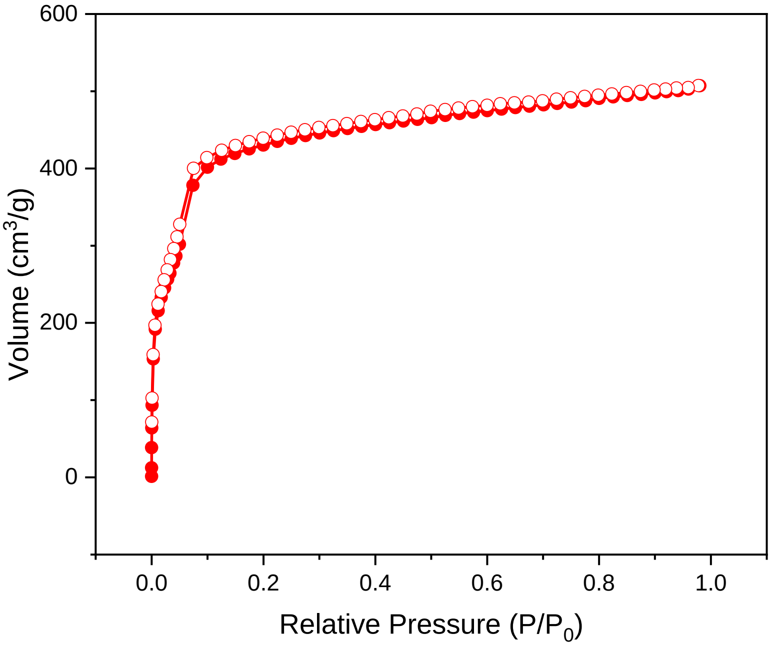

Figure S52: N_2 sorption isotherm of TTI-COF. Filled dots represent data points of the adsorption branch, hollow dots those of the desorption branch, respectively.

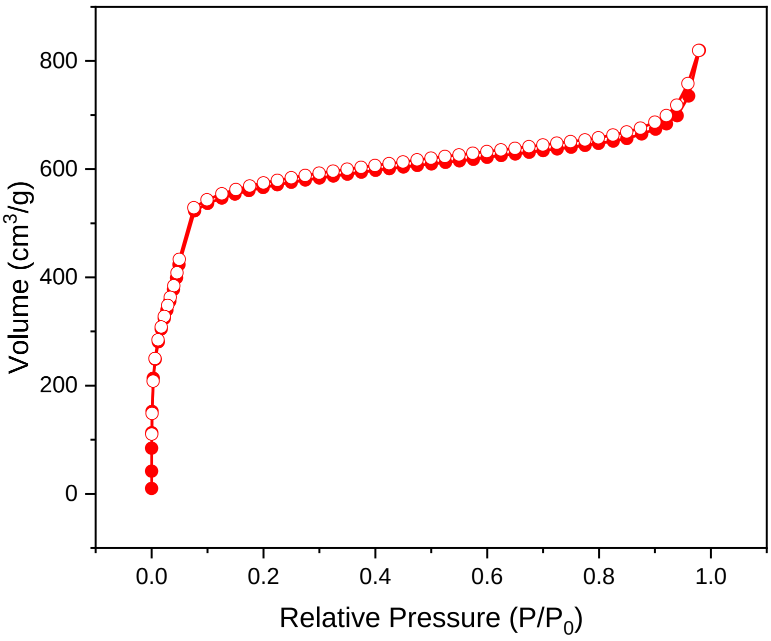

Figure S53: N_2 sorption isotherm of Py1P-COF. Filled dots represent data points of the adsorption branch, hollow dots those of the desorption branch, respectively.

2.4.2 Sorption Isotherms of Amine-linked COFs

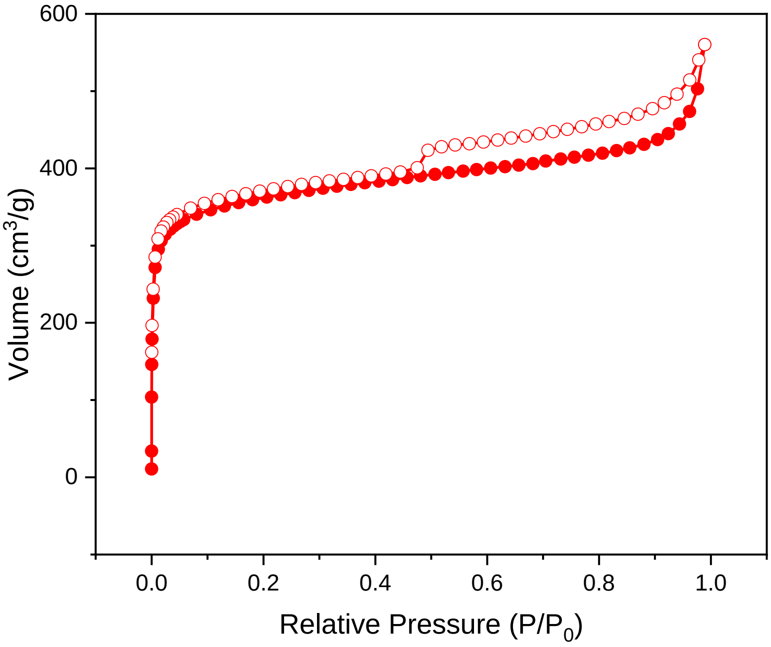

Figure S54: N_2 sorption isotherm of rPI-3-COF. Filled dots represent data points of the adsorption branch, hollow dots those of the desorption branch, respectively.

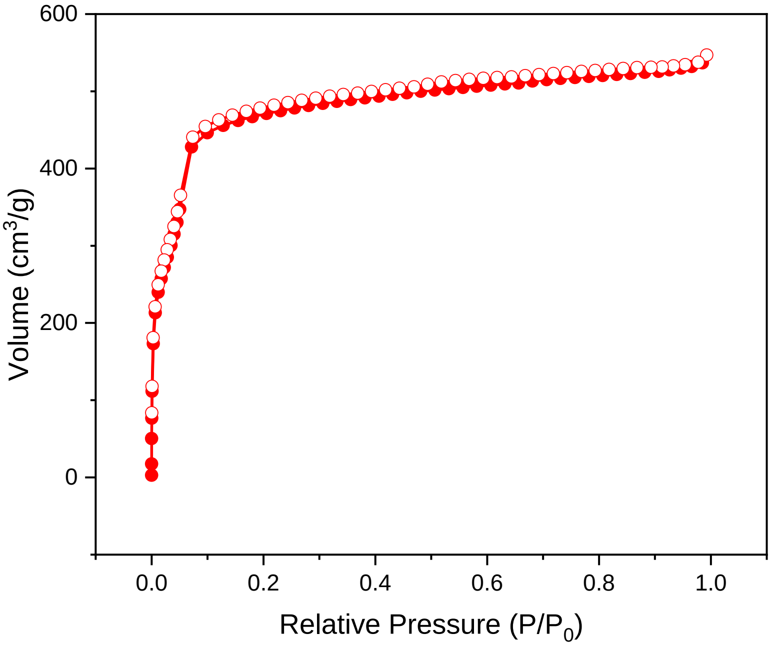

Figure S55: N_2 sorption isotherm of rTTI-COF. Filled dots represent data points of the adsorption branch, hollow dots those of the desorption branch, respectively.

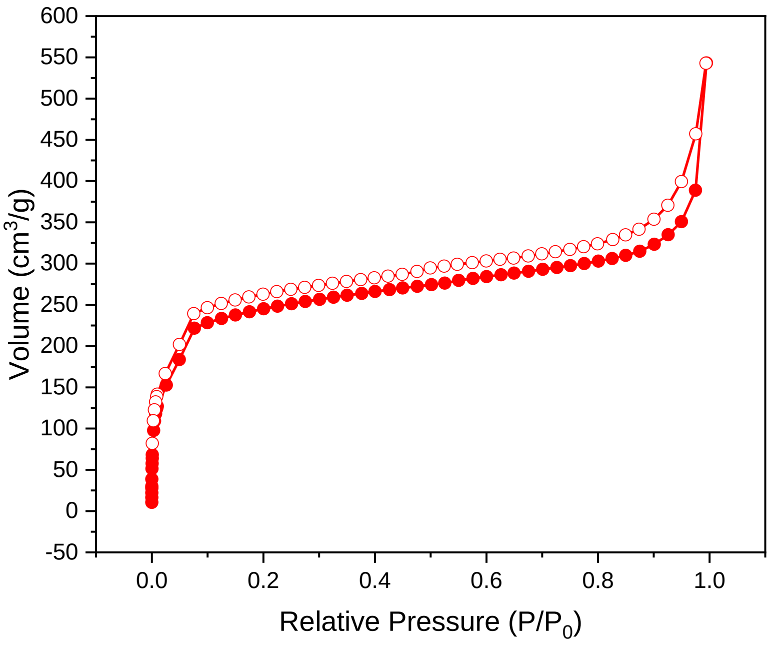

Figure S56: N_2 sorption isotherm of rPy1P-COF. Filled dots represent data points of the adsorption branch, hollow dots those of the desorption branch, respectively.

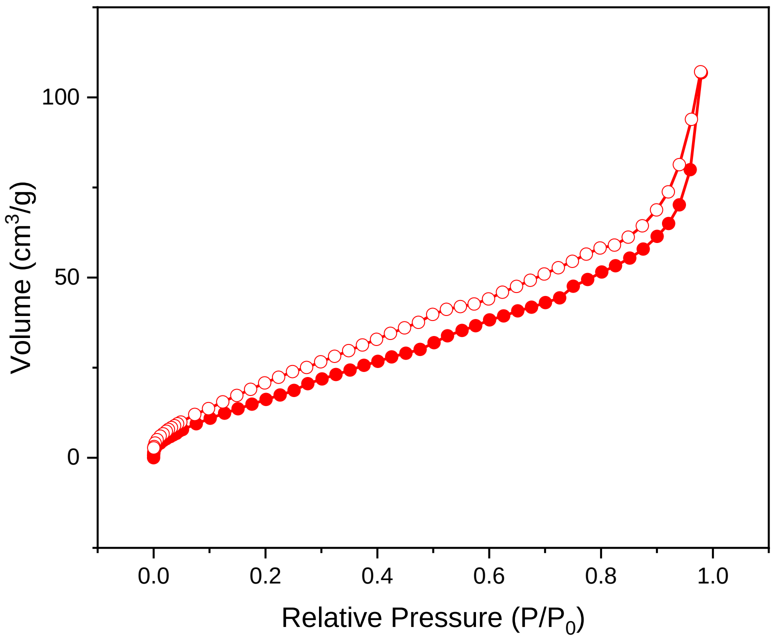

Figure S57: N_2 sorption isotherm of disordered rPI-3-COF. Filled dots represent data points of the adsorption branch, hollow dots those of the desorption branch, respectively.

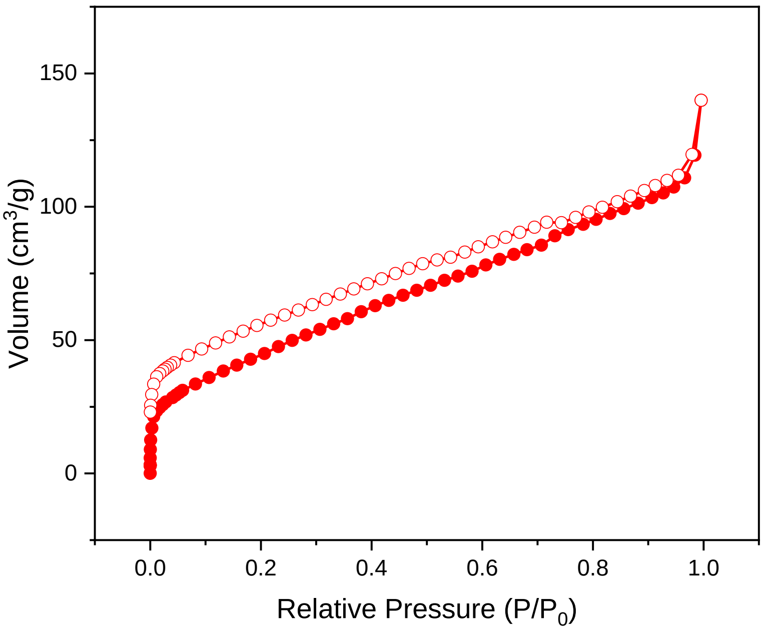

Figure S58: N_2 sorption isotherm of rPI-3-COF obtained from one-pot crystallization/reduction. Filled dots represent data points of the adsorption branch, hollow dots those of the desorption branch, respectively.

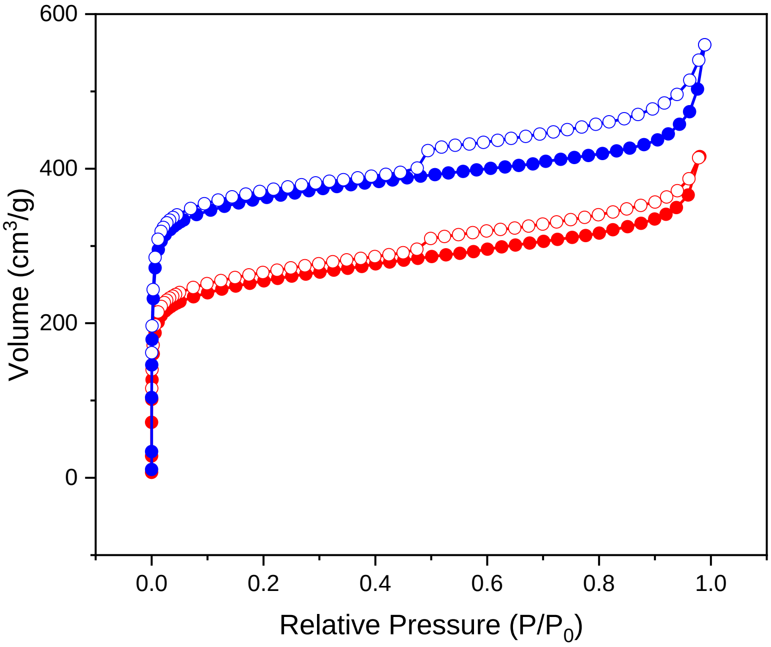

Figure S59: N_2 sorption isotherm comparison of different samples of rPI-3-COF. The activated sample ($scCO_2$ extraction; blue) shows a significant increase in nitrogen adsorption volume, compared to the non-activated sample (red). Filled dots represent data points of the adsorption branch, hollow dots those of the desorption branch, respectively.

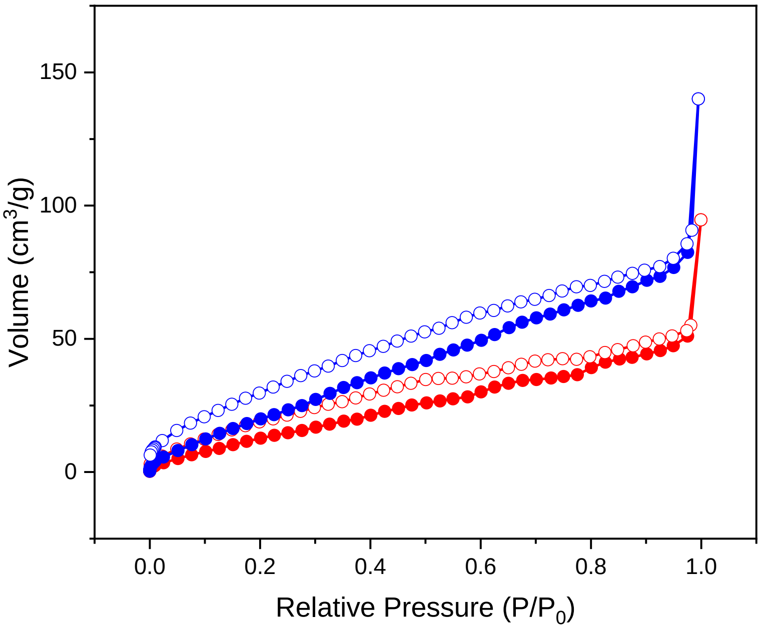

Figure S60: N_2 sorption isotherm comparison of BzCl-rTTI (blue) and TDI-rTTI (red). Both samples show a significantly reduced N_2 uptake, due to clogged pores after functionalization with an excess of the corresponding reagent. Filled dots represent data points of the adsorption branch, hollow dots those of the desorption branch, respectively.

2.4.3 Pore Size Distribution in Imine-Linked COFs

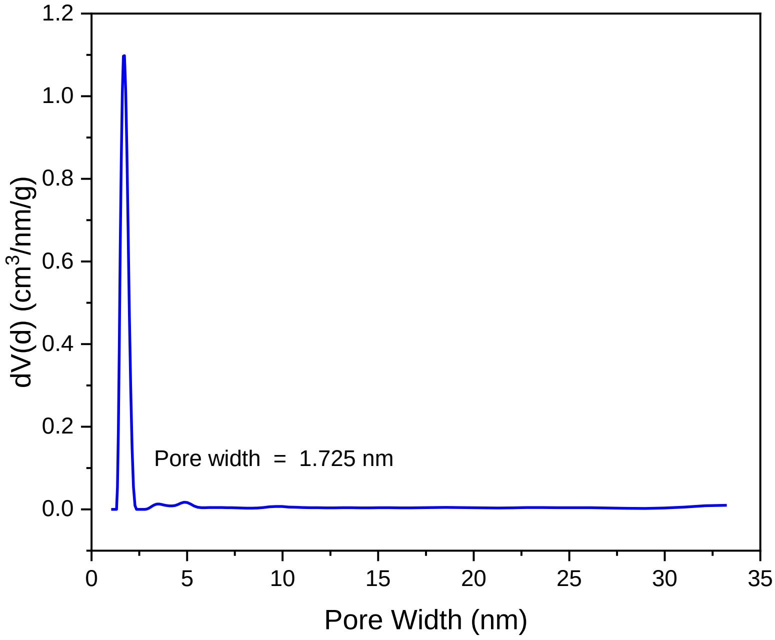

Figure S61: Pore size distribution in PI-3-COF.

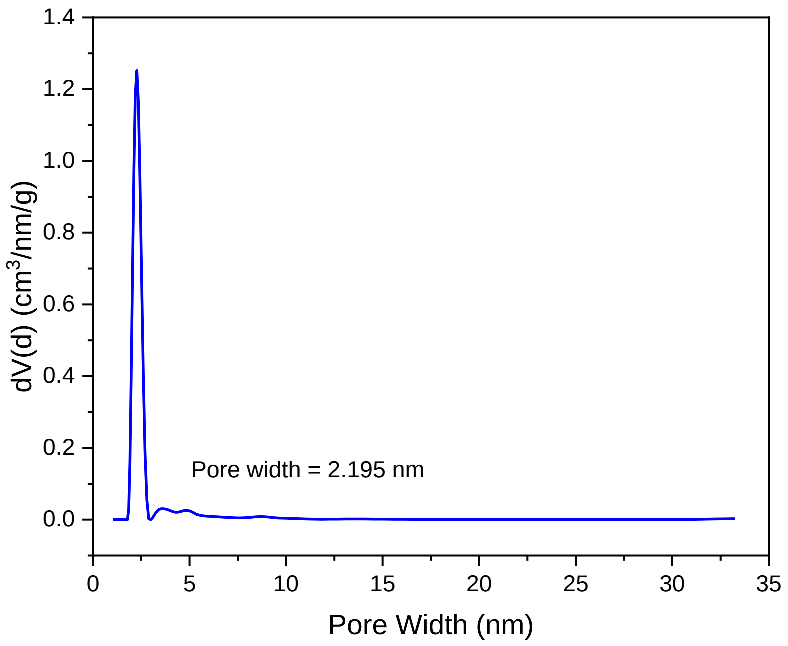

Figure S62: Pore size distribution in TTI-COF.

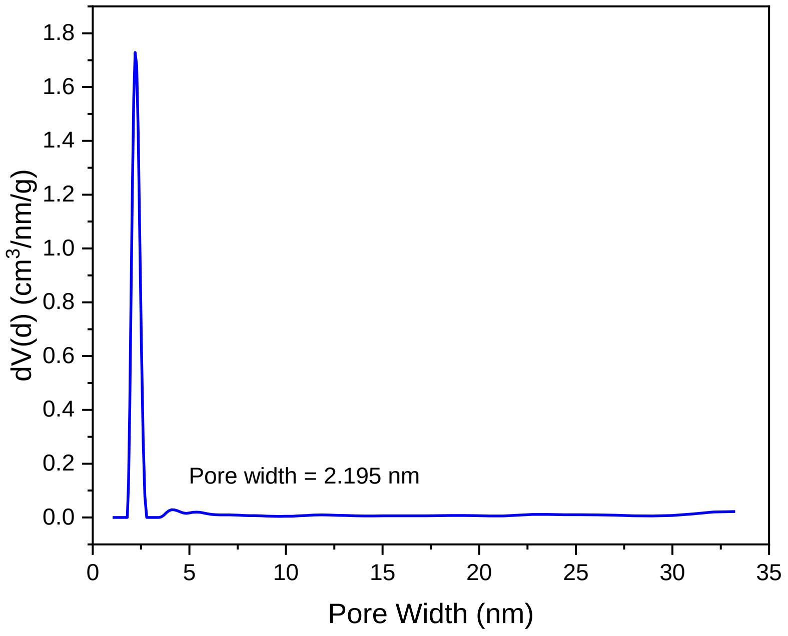

Figure S63: Pore size distribution in Py1P-COF.

2.4.4 Pore Size Distribution in Amine-linked COFs

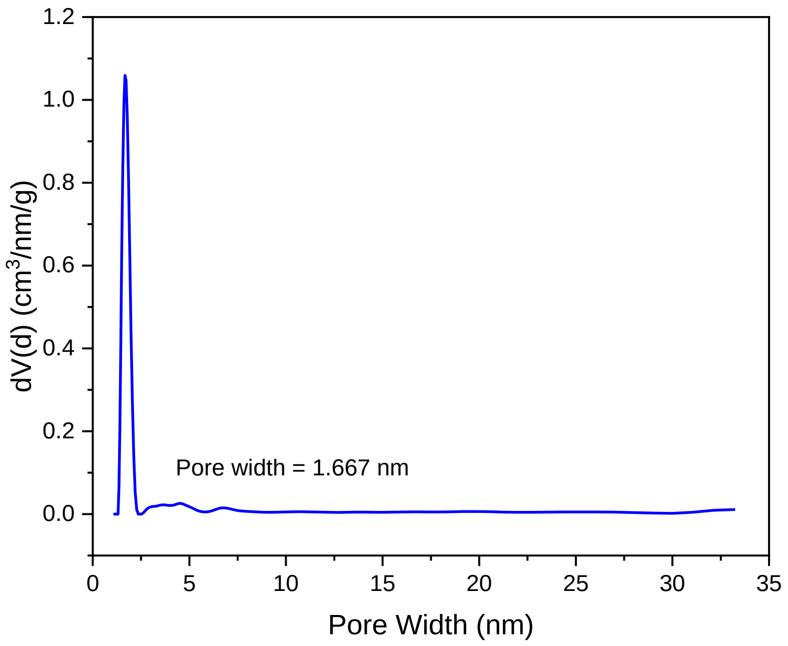

Figure S64: Pore size distribution in rPI-3-COF.

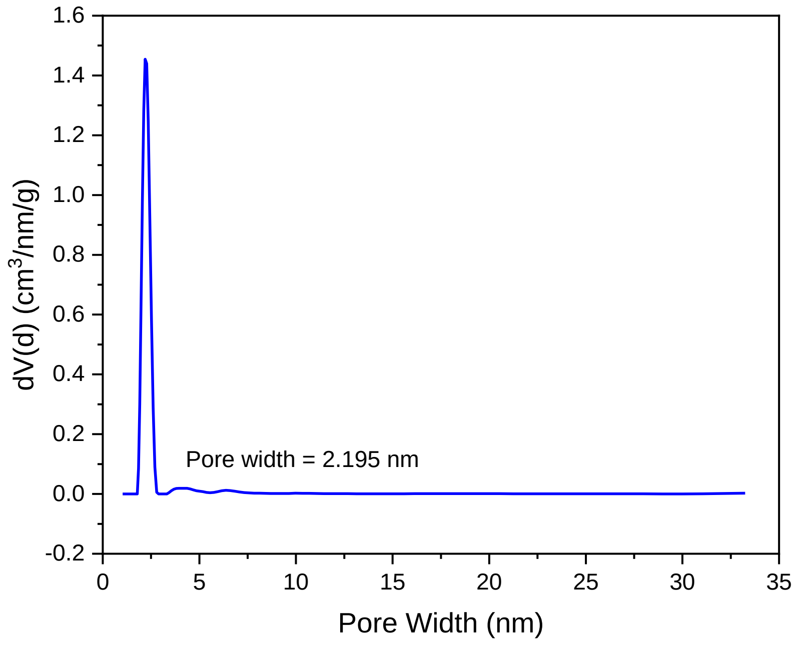

Figure S65: Pore size distribution in rTTI-COF.

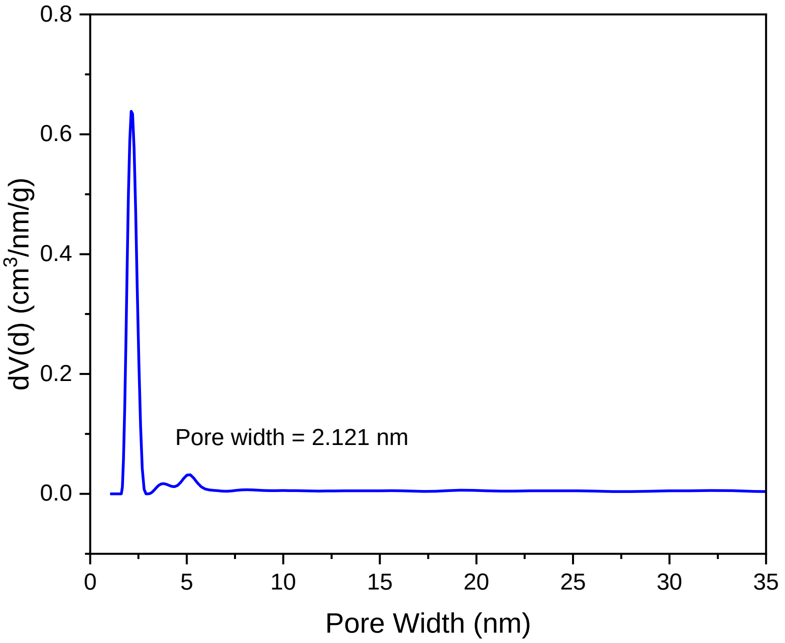

Figure S66: Pore size distribution in rPy1P-COF.

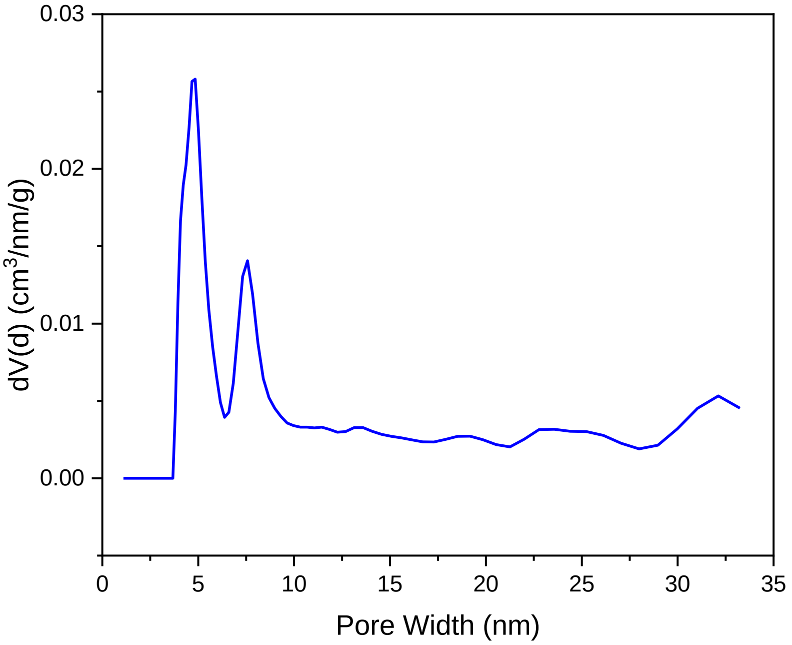

Figure S67: Pore size distribution in disordered rPI-3-COF.

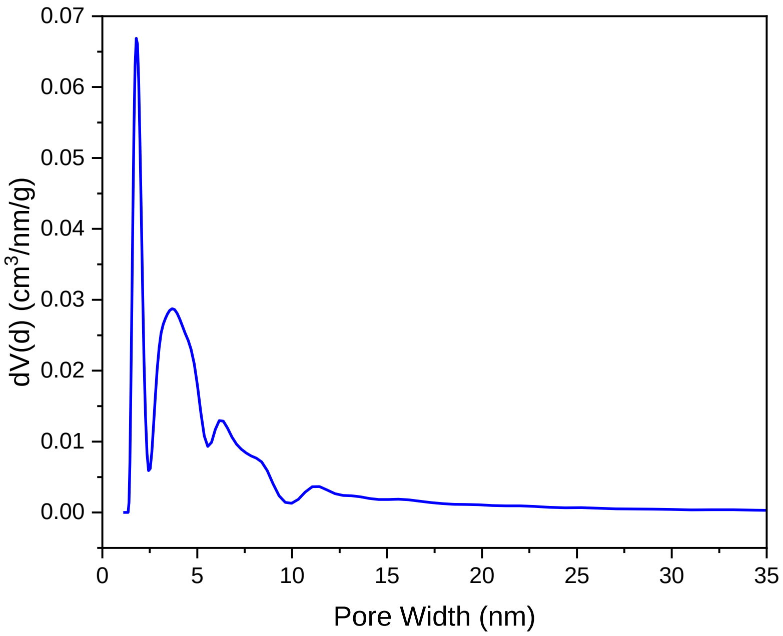

Figure S68: Pore size distribution rPI-3-COF obtained from one-pot crystallization/reduction.

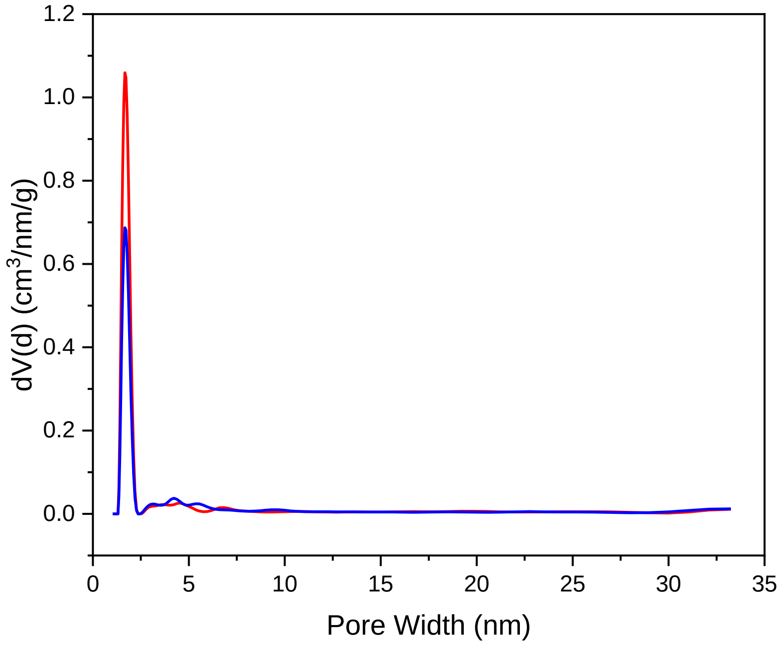

Figure S69: Pore size distribution comparison of different samples of rPI-3-COF. The activated sample ($scCO_2$ extraction; red) shows a significant increase in nitrogen adsorption volume, compared to the non-activated sample (blue).

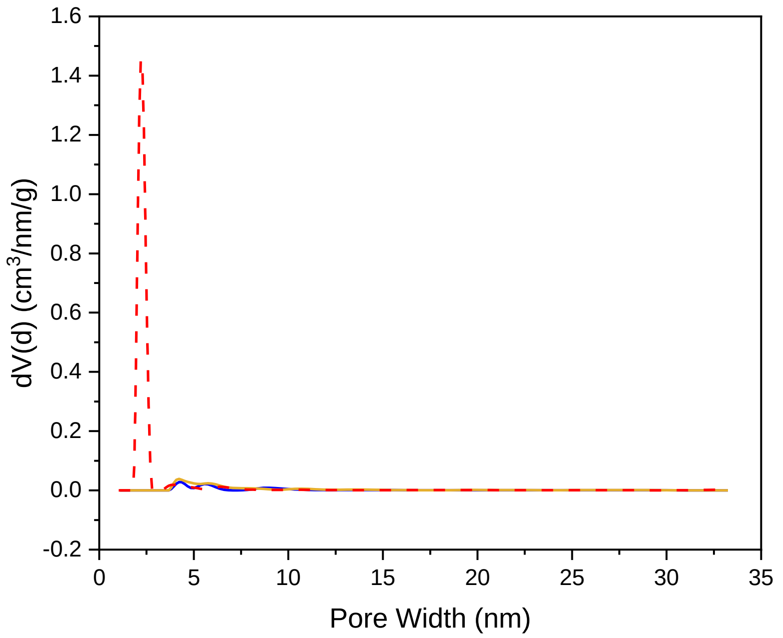

Figure S70: Pore size distribution comparison of rTTI-COF (red) and its functionalized derivatives BzCl-rTTI-COF (orange) and TDI-rTTI-COF (blue), which show a significant decrease in nitrogen adsorption volume around the pore-diameter of 2.195 nm, due to pore clogging.

2.4.5 BET Plots

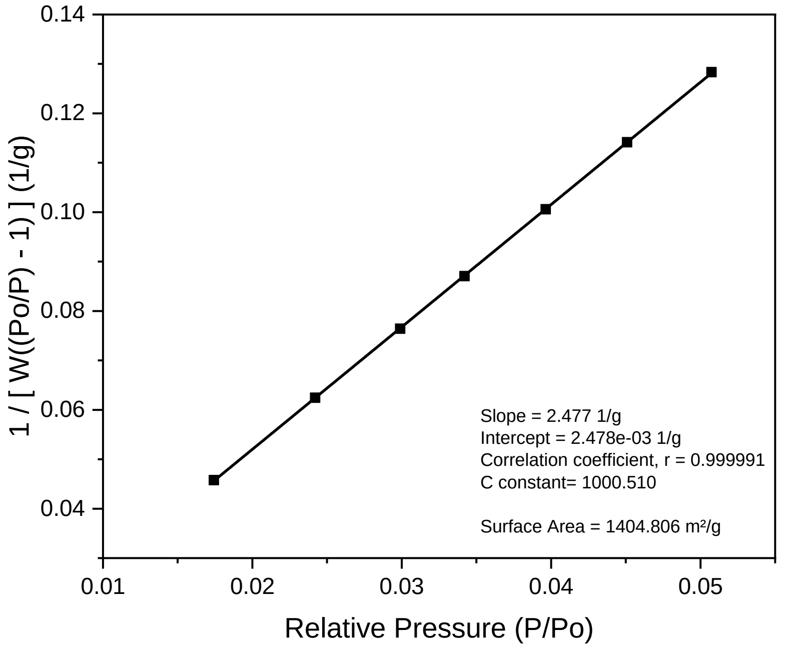

Figure S71: BET-Plot of PI-3-COF.

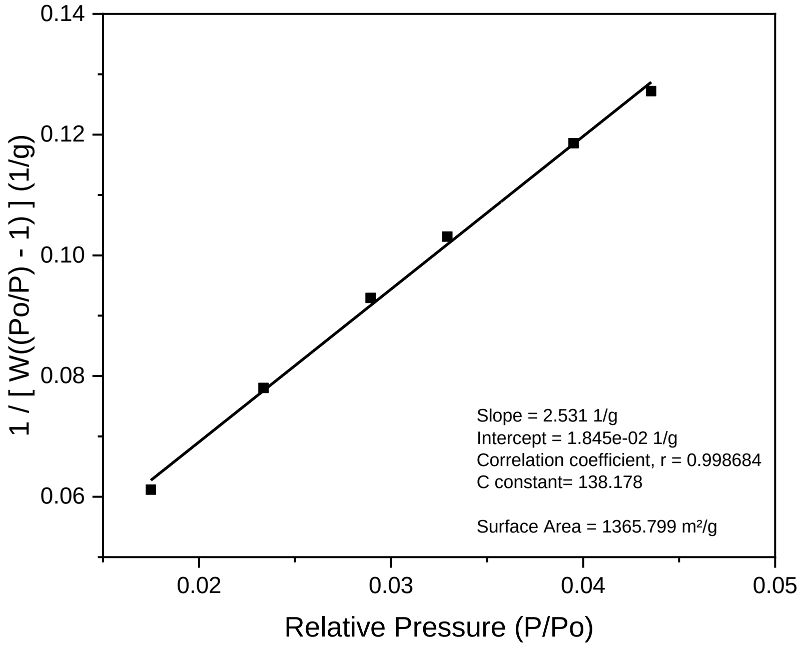

Figure S72: BET-Plot of TTI-COF.

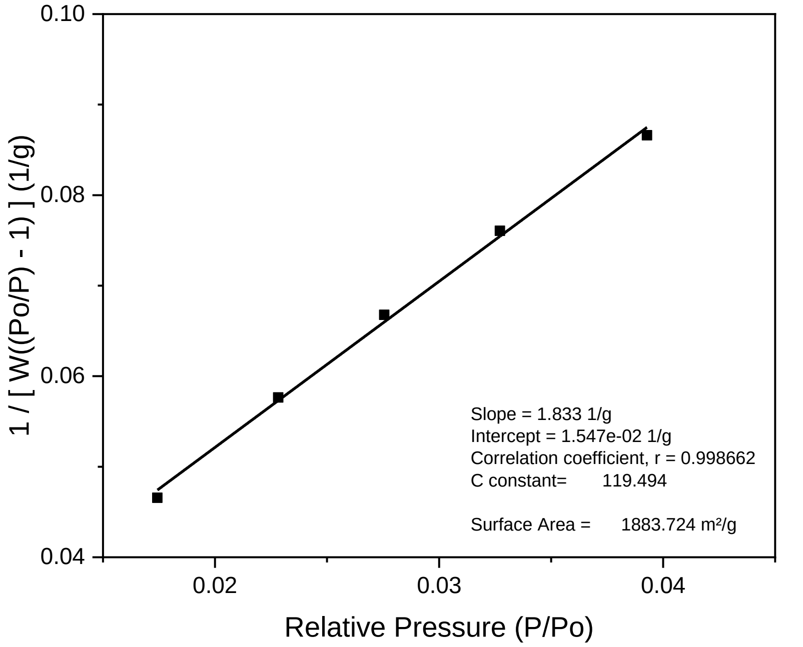

Figure S73: BET-Plot of Py1P-COF.

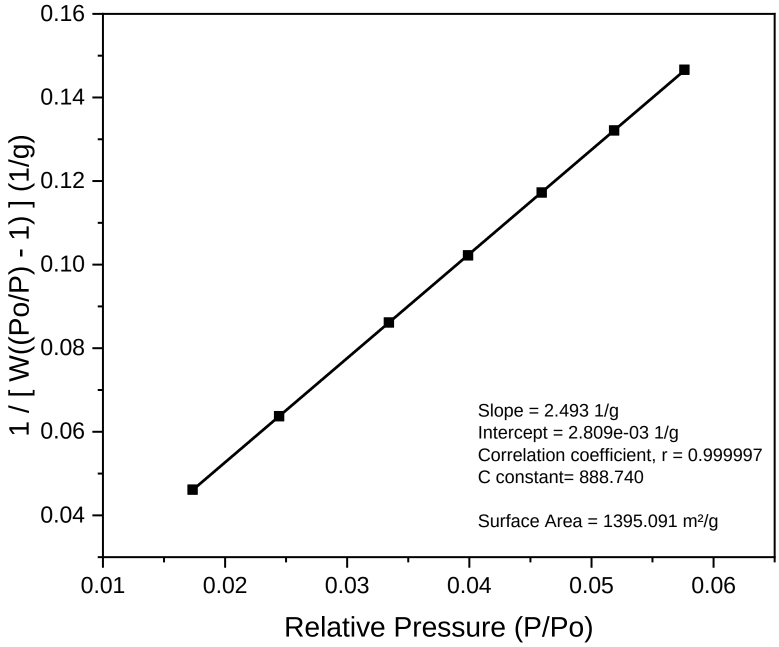

Figure S74: BET-Plot of rPI-3-COF.

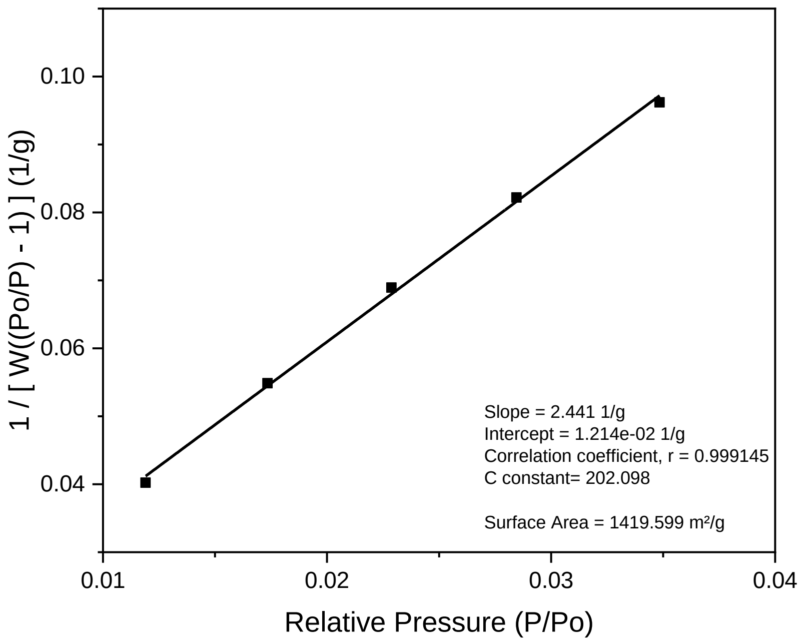

Figure S75: BET-Plot of rTTI-COF.

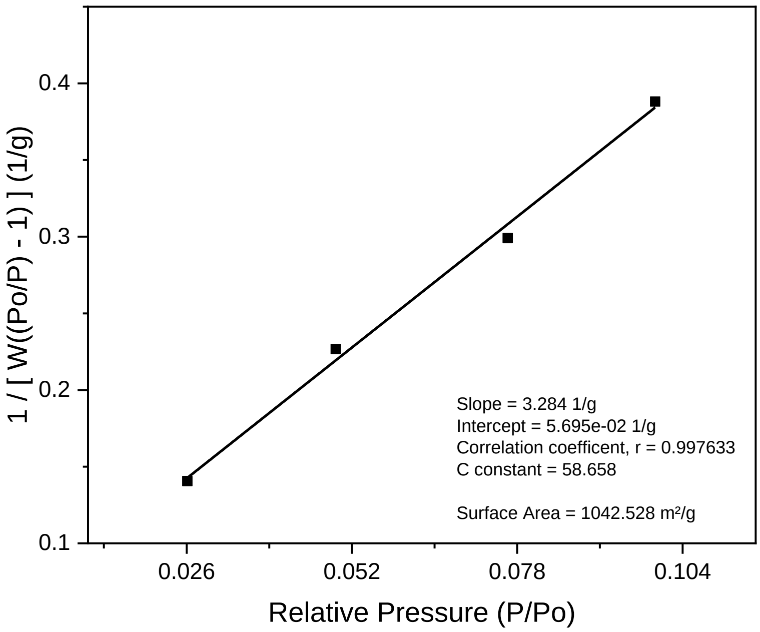

Figure S76: BET-Plot of rPy1P-COF.

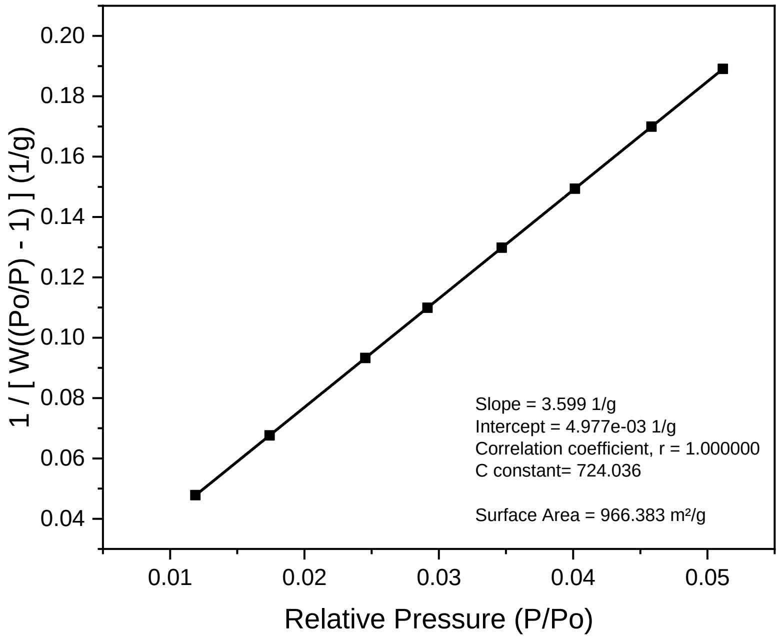

Figure S77: BET-Plot of rPI-3-COF, without $scCO_2$ activation.

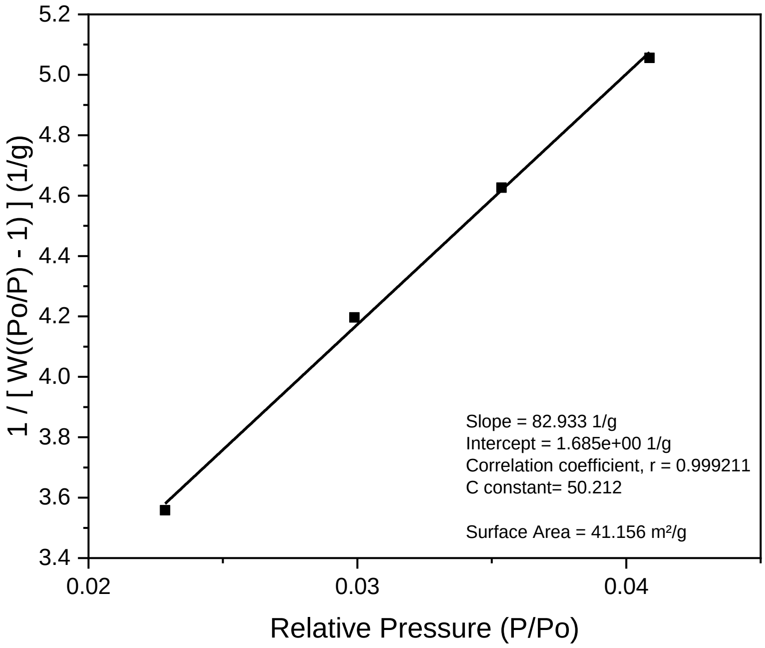

Figure S78: BET-Plot of disordered rPI-3-COF.

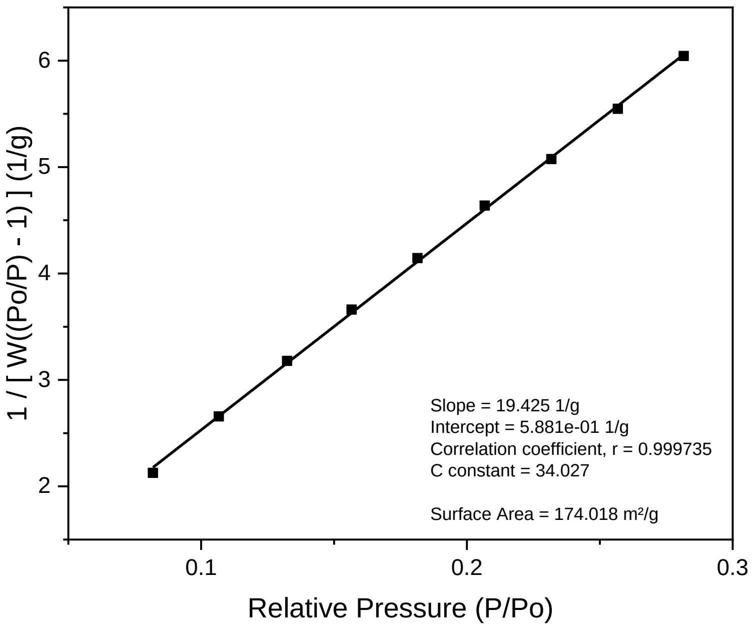

Figure S79: BET-Plot of rPI-3-COF obtained from one-pot crystallization/reduction.

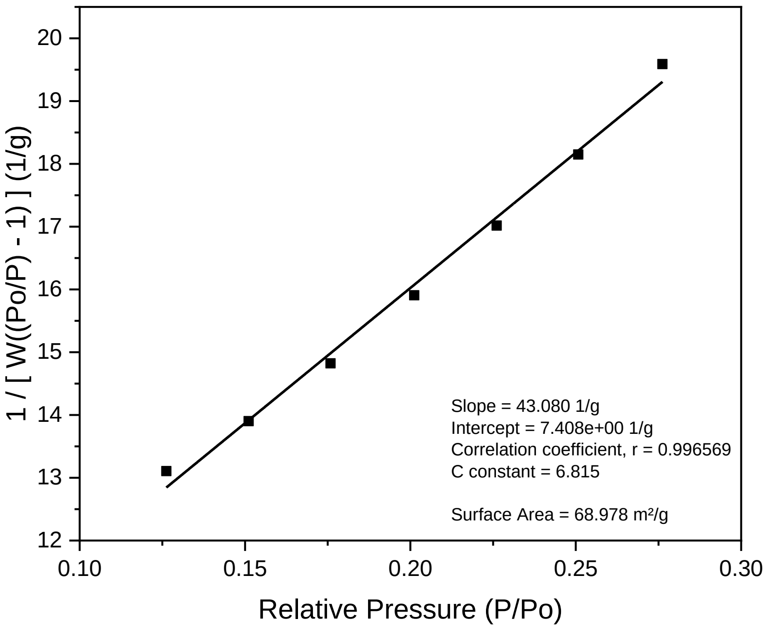

Figure S80: BET-Plot of TDI-rTTI-COF.

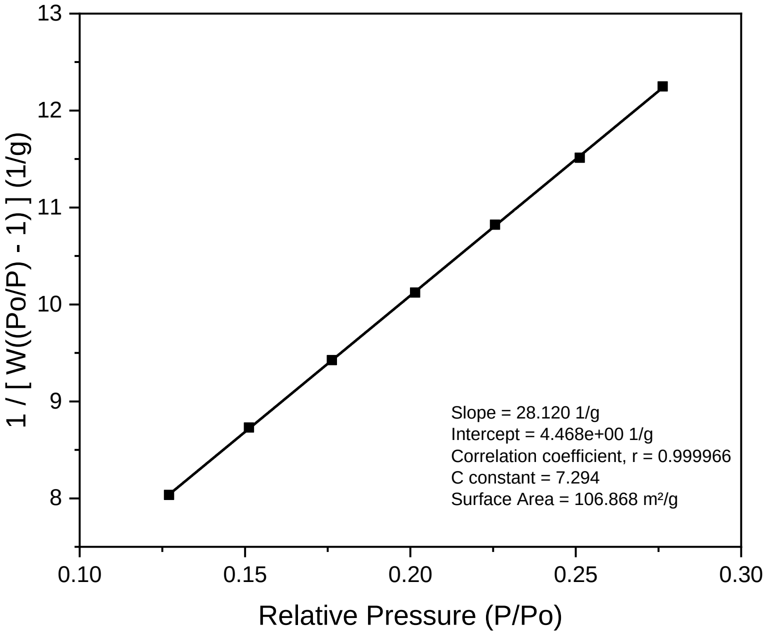

Figure S81: BET-Plot of BzCl-rTTI-COF.

2.5 Scanning Electron Microscopy

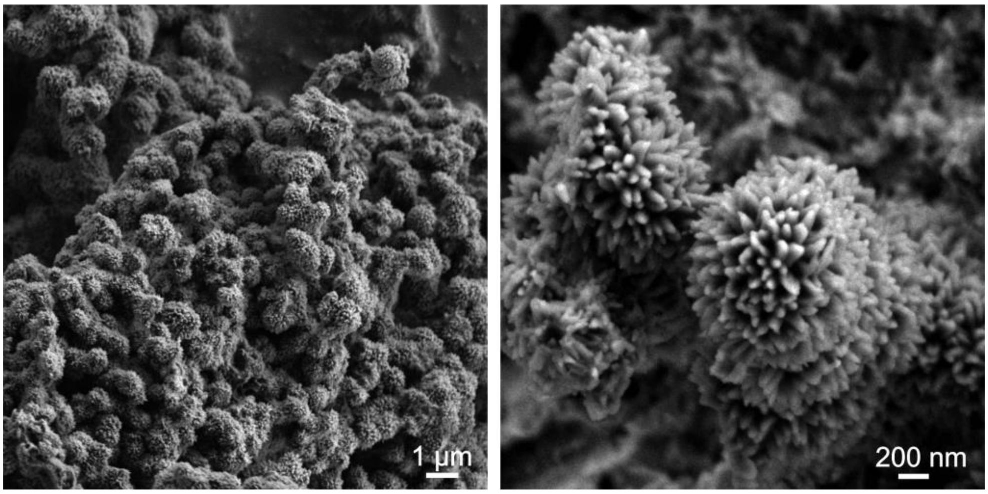

Figure S82: SEM images of PI-3-COF.

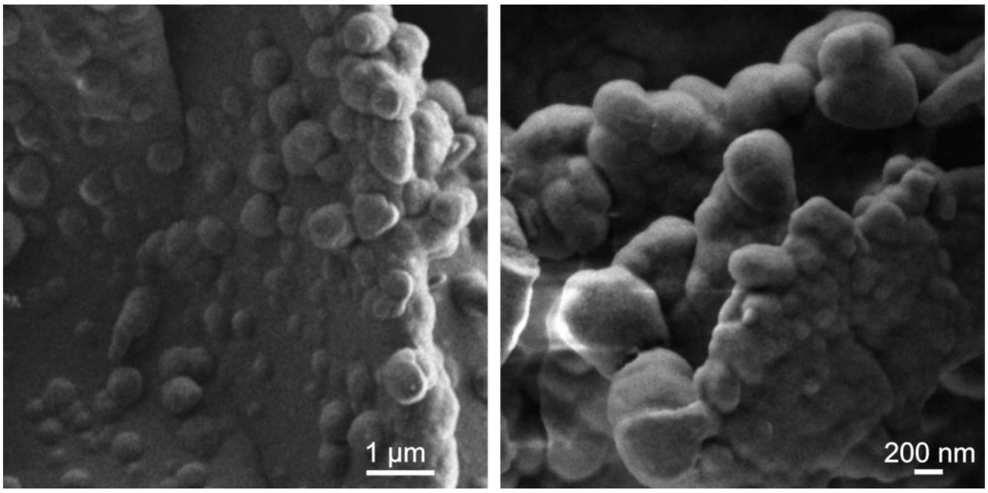

Figure S83: SEM images of TTI-COF.

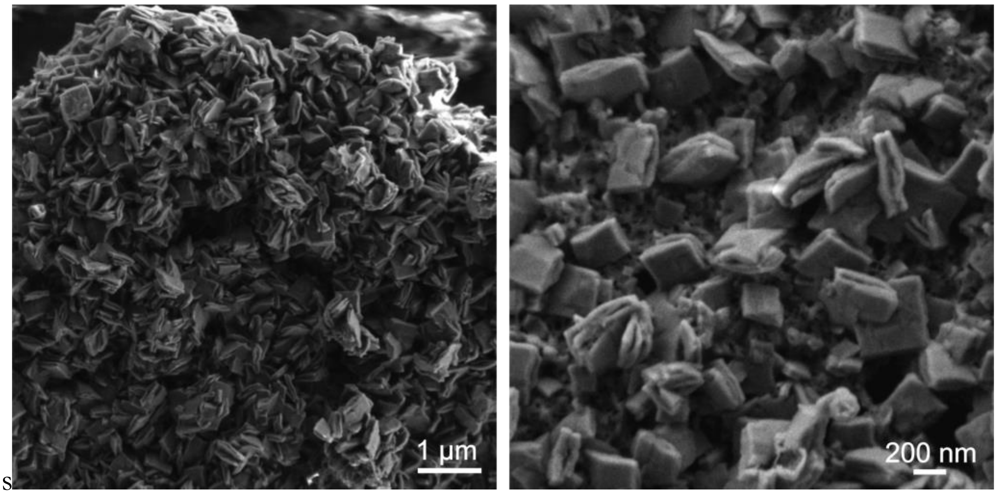

Figure S84: SEM images of Py1P-COF.

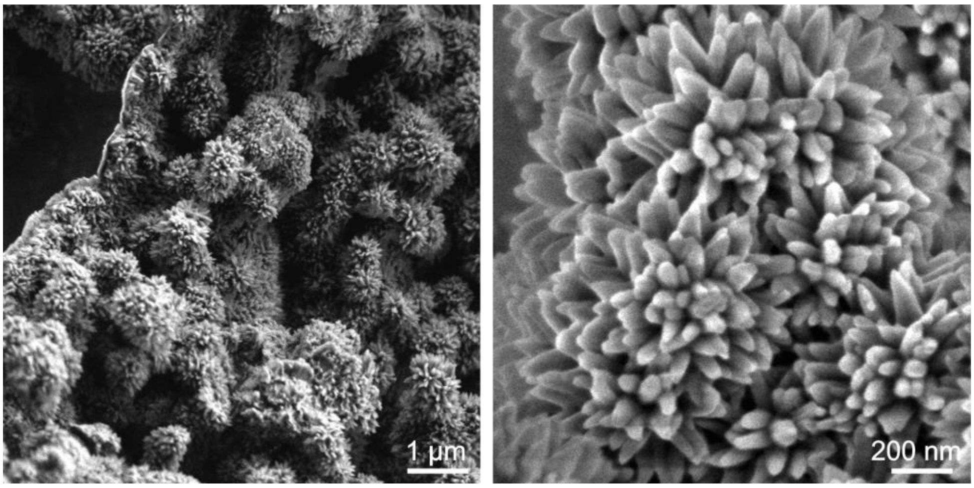

Figure S85: SEM images of rPI-3-COF.

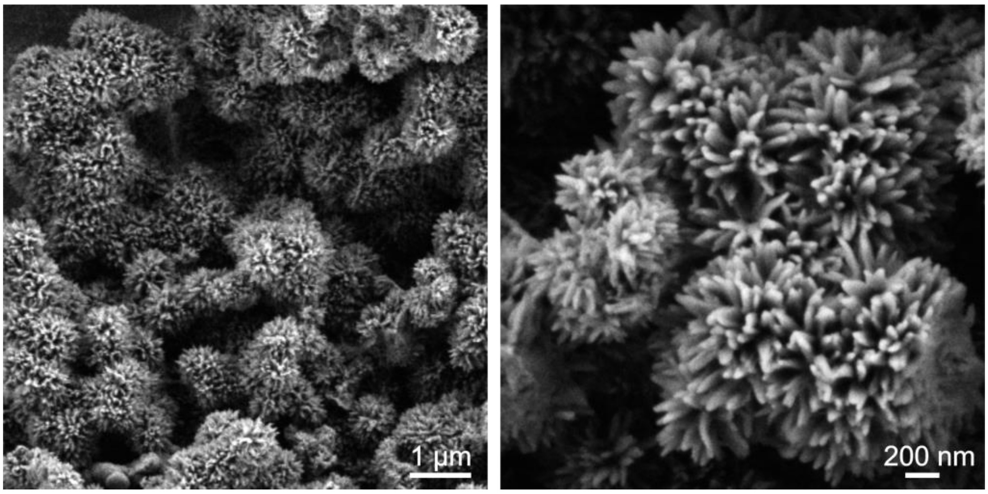

Figure S86: SEM images of disordered rPI-3-COF.

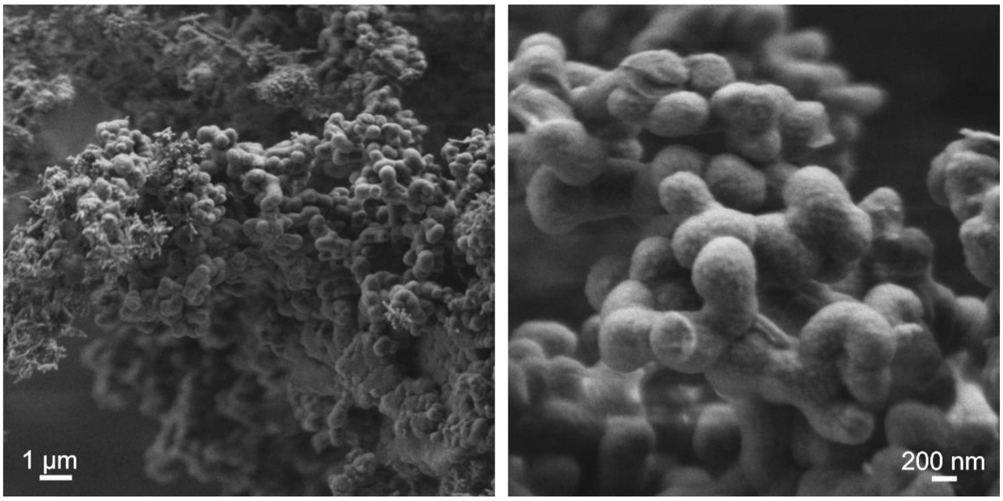

Figure S87: SEM images of rPI-3-COF obtained by one-pot crystallization/reduction.

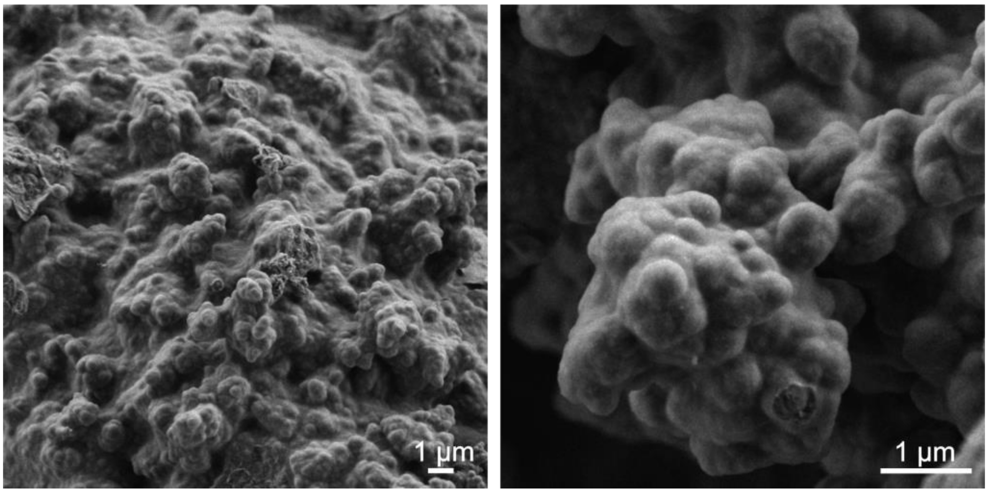

Figure S88: SEM images of rTTI-COF.

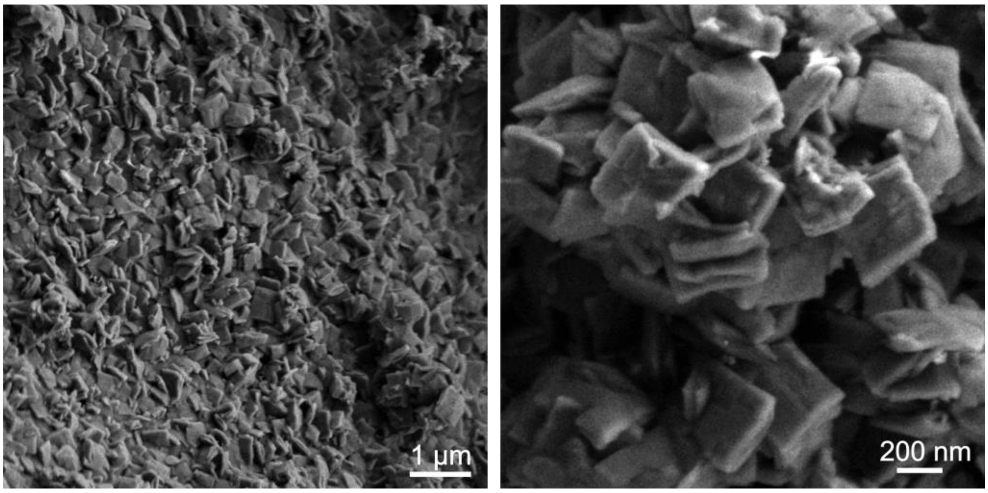

Figure S89: SEM images of rPy1P-COF.

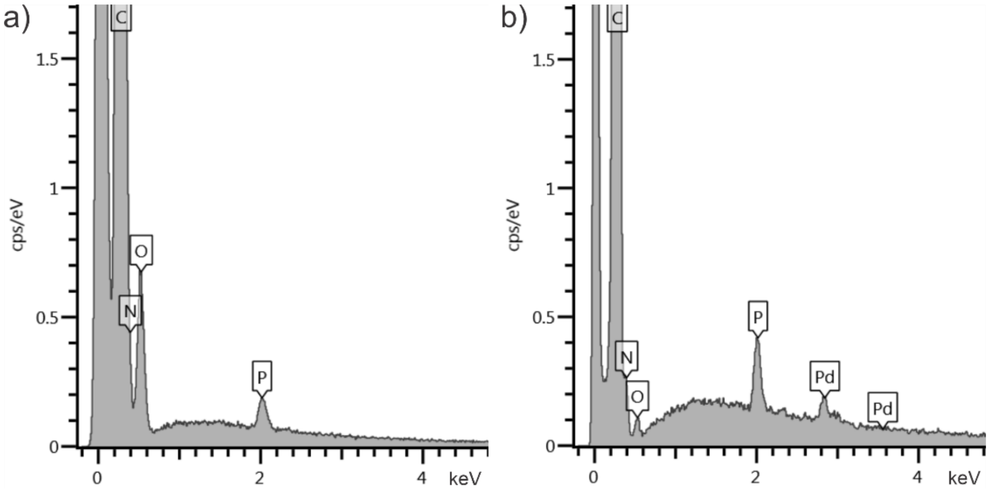

Figure S90: SEM-EDX spectra of a purified (a) and Pd-contaminated sample of 4,4',4'',4'''-(pyrene-1,3,6,8-tetrayl)tetraaniline measured at 20kV.

2.6 Transmission Electron Microscopy

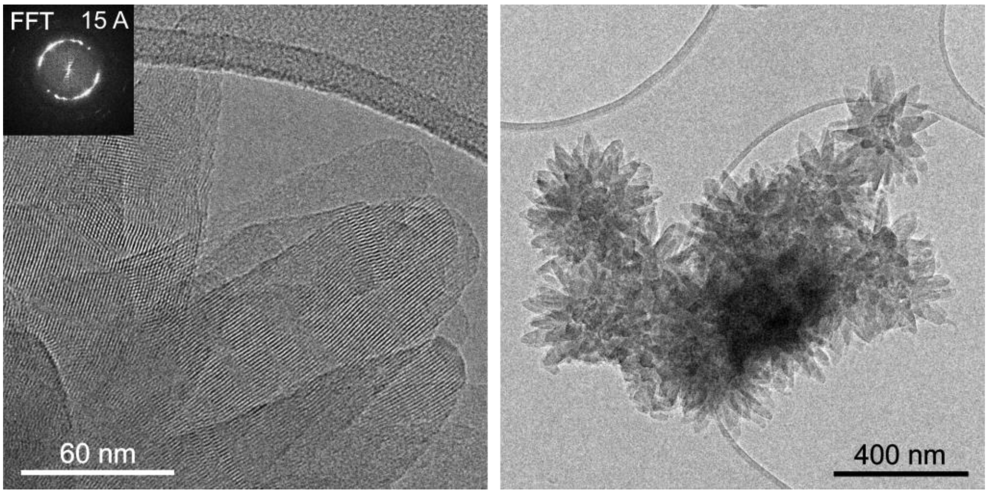

Figure S91: TEM images of PI-3-COF.

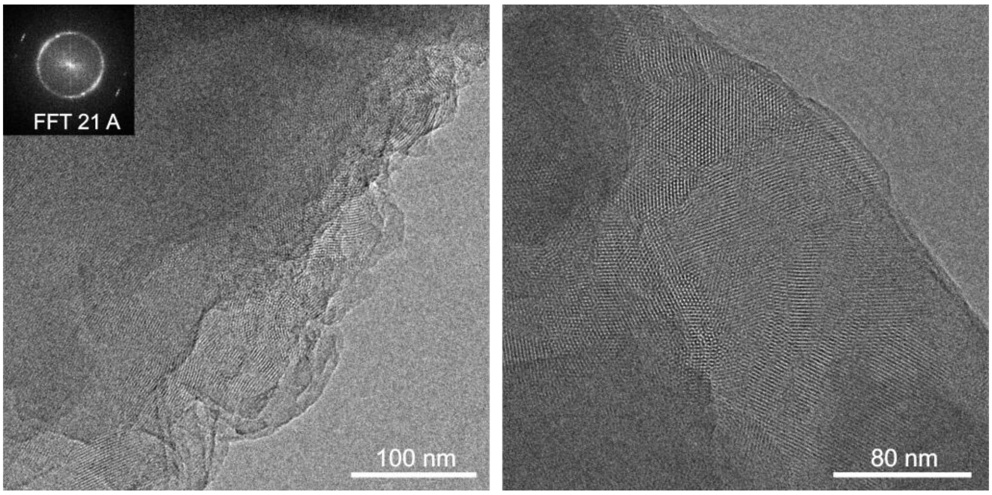

Figure S92: TEM images of TTI-COF.

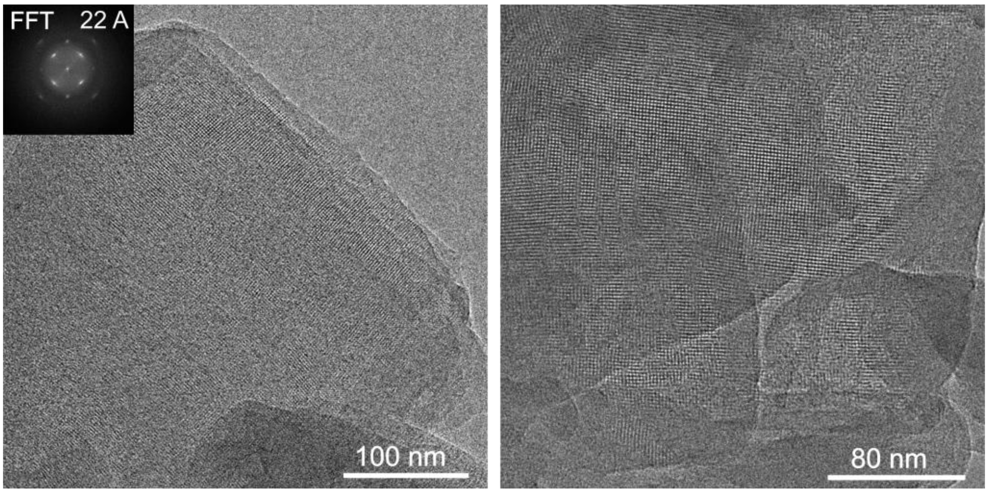

Figure S93: TEM images of Py1P-COF.

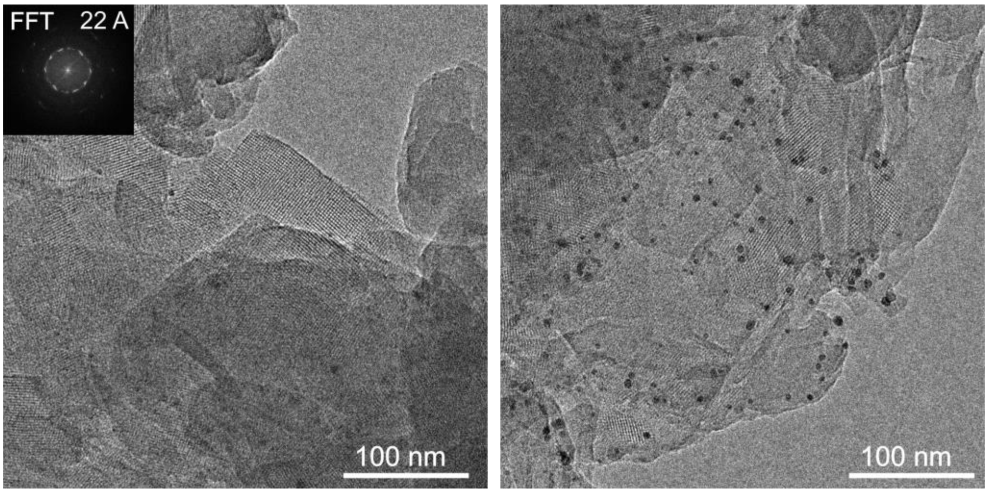

Figure S94: TEM images of a Pd-contaminated (black dots) Py1P-COF sample.

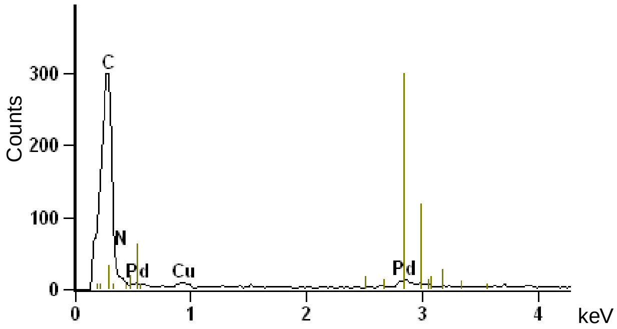

Figure S95: TEM-EDX spectrum (point analysis) of a Pd-contaminated Py1P-COF sample (black). For comparison, a spectrum for palladium is shown in green.

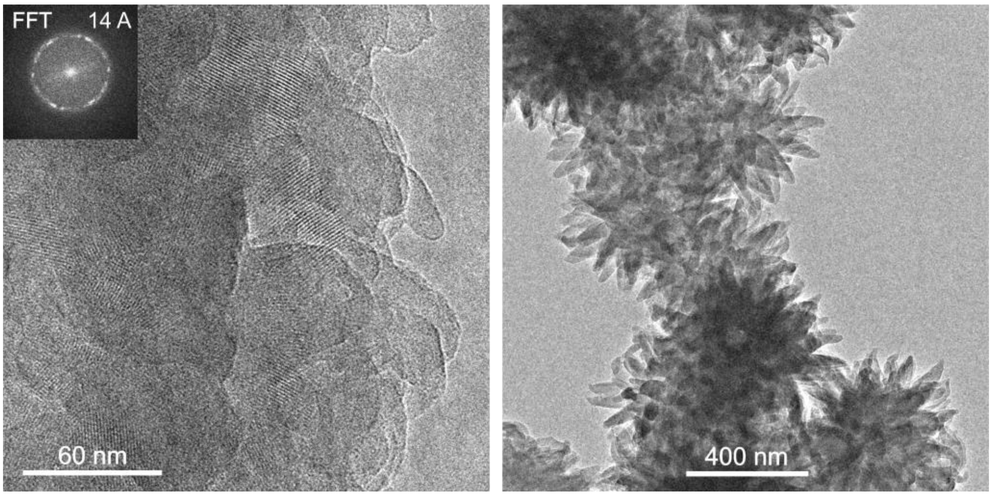

Figure S96: TEM images of rPI-3-COF.

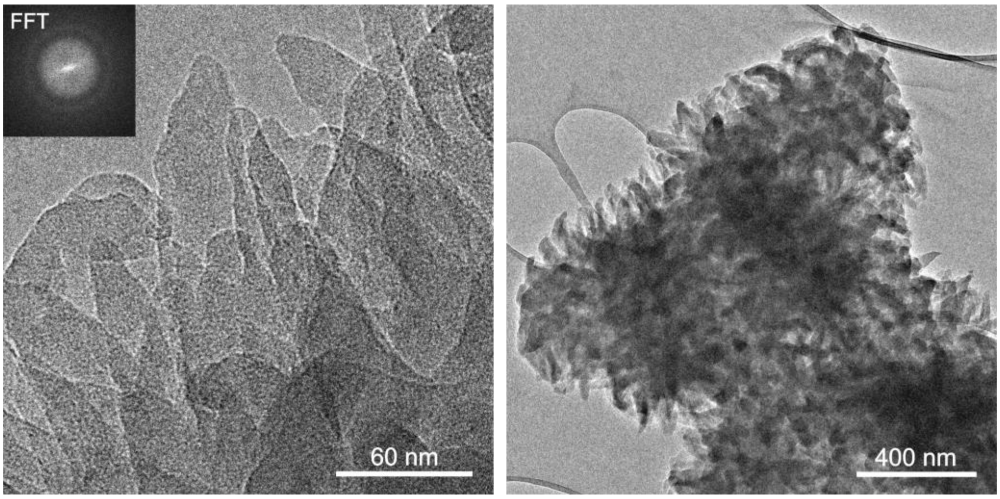

Figure S97: TEM images of disordered rPI-3-COF.

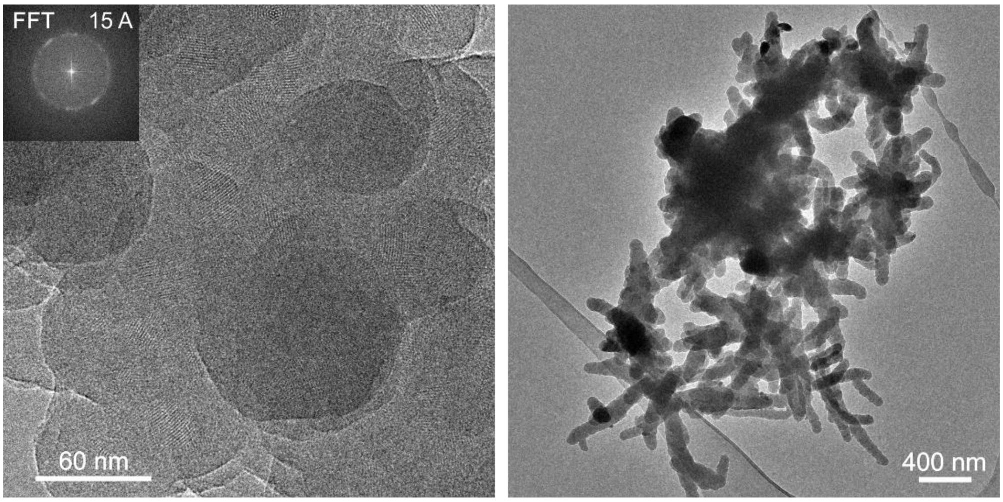

Figure S98: TEM images of rPI-3-COF obtained by one-pot crystallization/reduction.

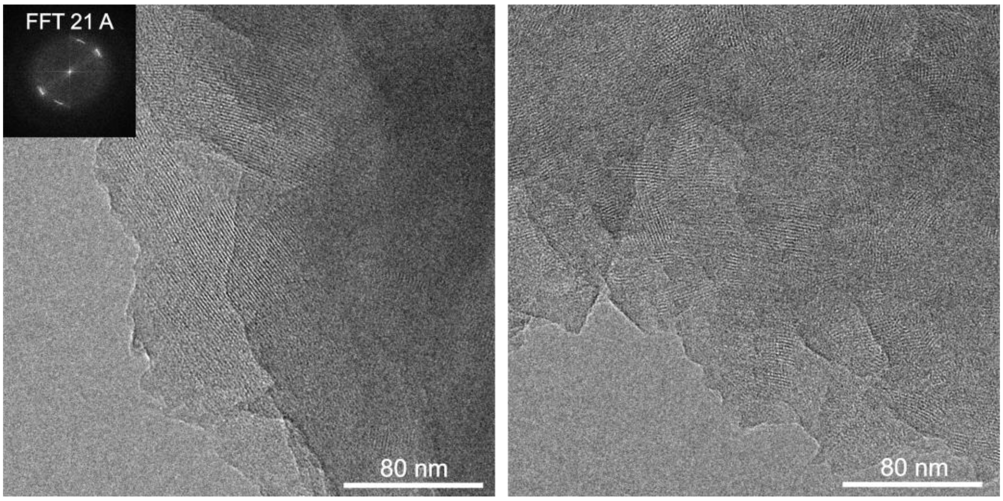

Figure S99: TEM images of rTTI-COF.

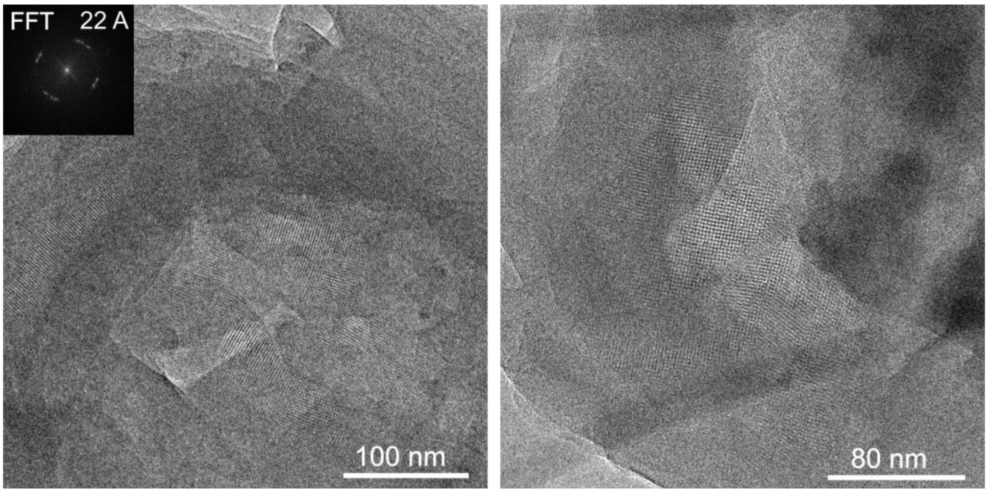

Figure S100: TEM images of rPy1P-COF.

2.7 Stability Tests

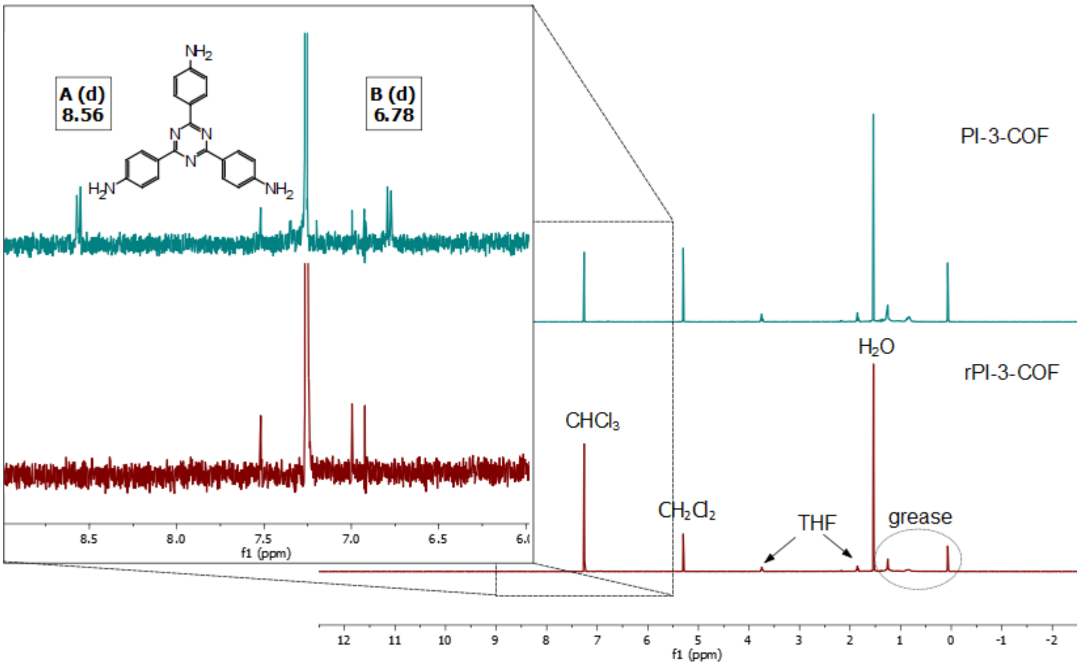

Figure S101: ^{1}H-NMR spectrum (400 MHz, $CDCl_3$) of degradation products from hydrolysis of PI-3-COF and rPI-3-COF samples treated with 3M NaOH in a THF:Water (1:1) mixture. Only the imine-linked framework (PI-3-COF) decomposed partially (hydrolysis) and shows signals of the amine monomer.

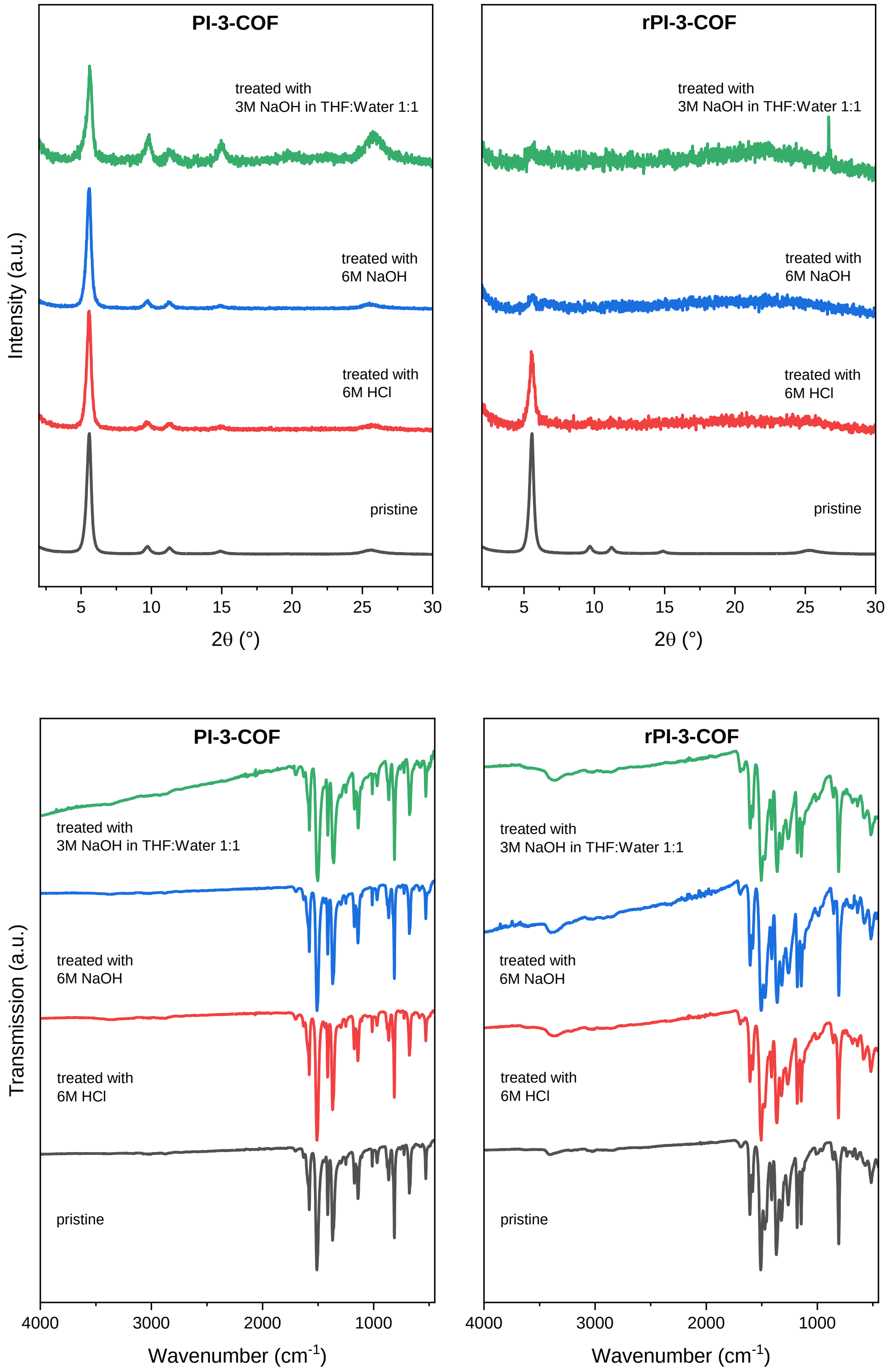

Figure S102: XRPD and FT-IR comparison of amine- and imine-linked COF samples stirred in acidic and basic solutions for 24h. Note that reduced crystallinity for rPI-3-COF is caused by increasing disorder in the framework instead of hydrolytic decomposition (see Figure S101).

3 Quantum-Chemical Calculations

3.1 Geometry Optimization

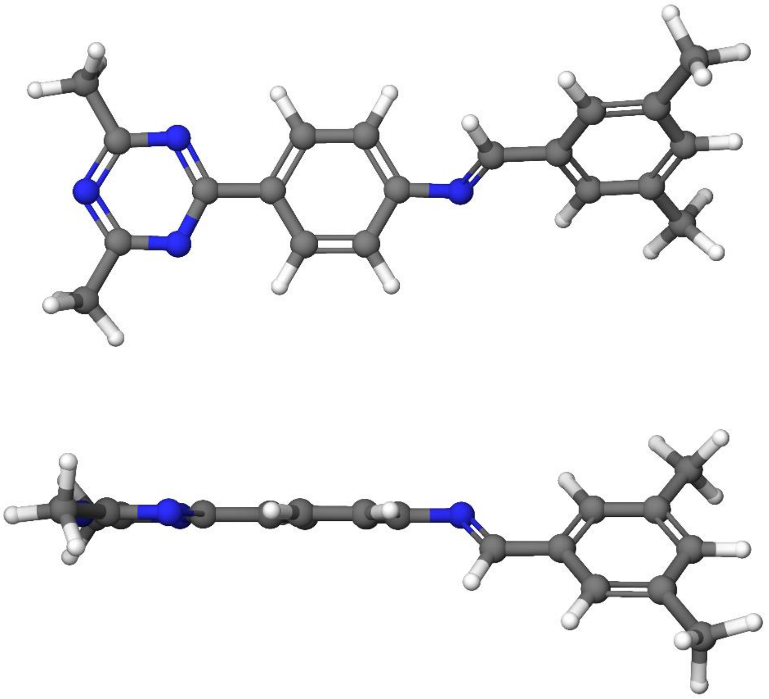

Figure S103: Optimized structure for a representative molecular model system of PI-3-COF (PI-3 M), obtained on PBE0-D3/def2-TZVP level of theory.

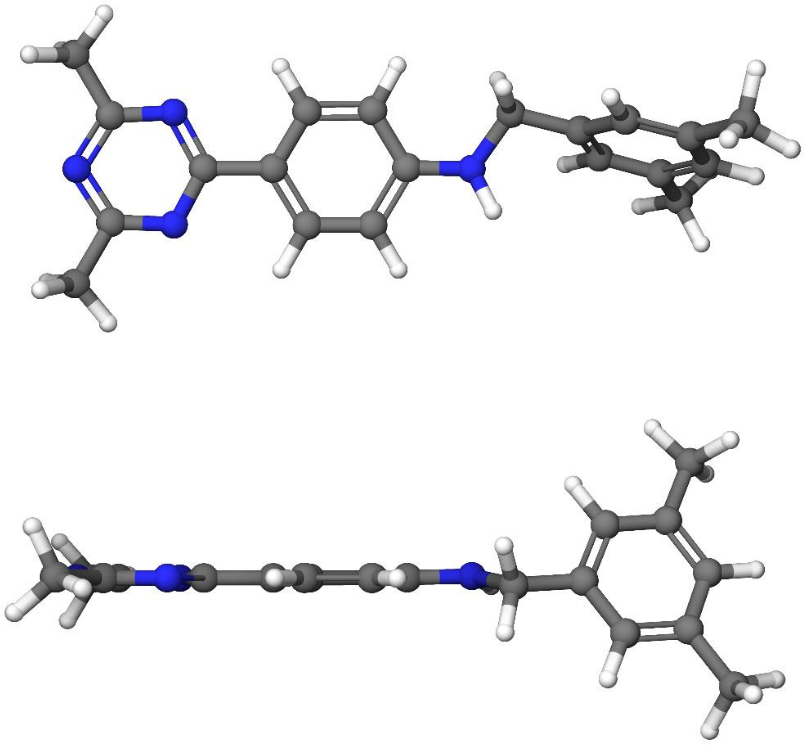

Figure S104: Optimized structure for a representative molecular model system of rPI-3-COF (rPI-3 M), obtained on PBE0-D3/def2-TZVP level of theory.

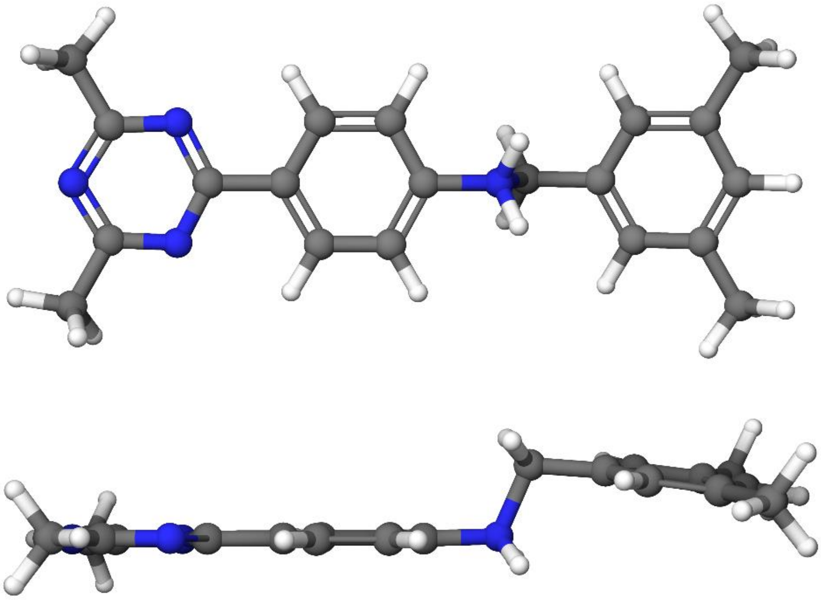

Figure S105: Optimized structure for a protonated representative molecular model system of rPI-3-COF (H+rPI-3 M), obtained on PBE0-D3/def2-TZVP level of theory. Note that the effect of the counter ion is neglected in this model.

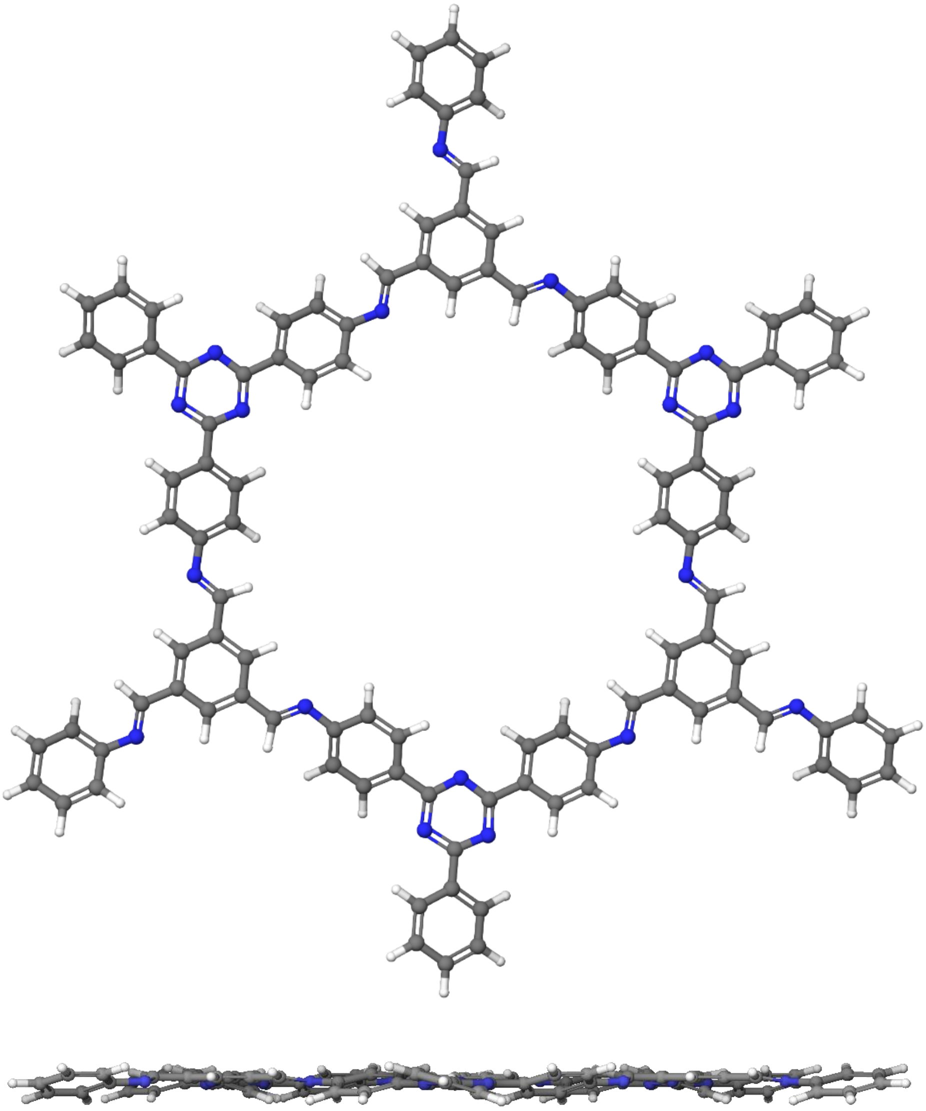

Figure S106: Optimized structure for a representative single-pore model system of PI-3-COF (PI-3 SP), obtained on RI-PBE-D3/def2-TZVP level of theory.

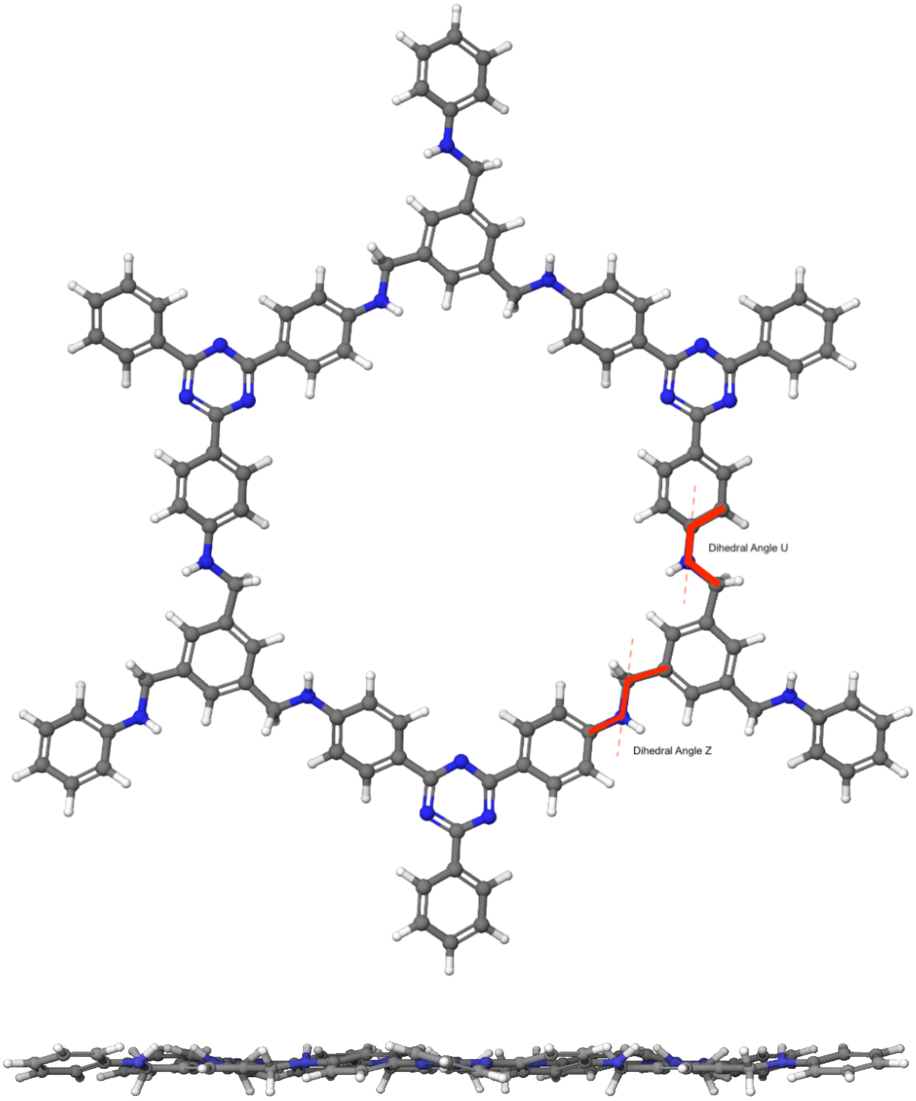

Figure S107: Optimized structure for a representative single-pore model system of rPI-3-COF (rPI-3 SP), obtained on RI-PBE-D3/def2-TZVP level of theory.

Table S4: Comparison of **U** and **Z** dihedral angles [°], obtained from optimized geometries for the presented model systems.

Model System	Level of Theory	U (CCNC)	ΔU (CCNC)	Z (CNCC)	ΔZ (CNCC)
PI-3 M	PBE0-D3/def2-TZVP	43.59		-176.82	
PI-3 M	RI-PBE-D3/def2-TZVP	43.29	-0.30	-175.80	1.02
PI-3 SP	RI-PBE-D3/def2-TZVP	27.40	-15.89	-177.64	-1.84
rPI-3 M	PBE0-D3/def2-TZVP	10.18		-179.68	
rPI-3 M	RI-PBE-D3/def2-TZVP	8.97	-1.21	-179.55	0.13
rPI-3 SP	RI-PBE-D3/def2-TZVP	6.46	-2.51	-178.51	1.04
H+rPI-3 M	PBE0-D3/def2-TZVP	-85.71		-179.57	
	RI-PBE-D3/def2-TZVP	-86.6	-0.89	-179.48	0.09

3.2 NMR Chemical Shifts

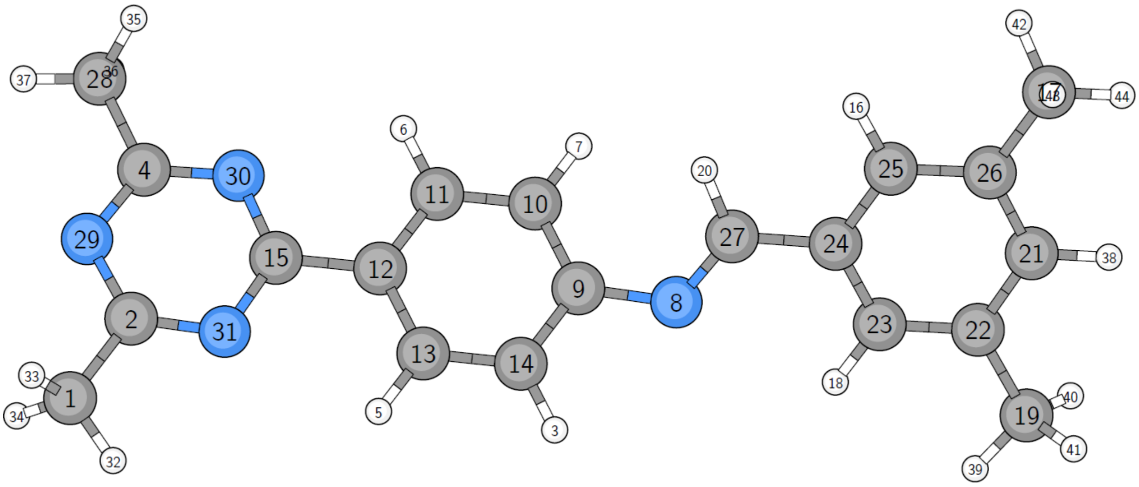

Figure S108: Atom labels for the PI-3 M model.

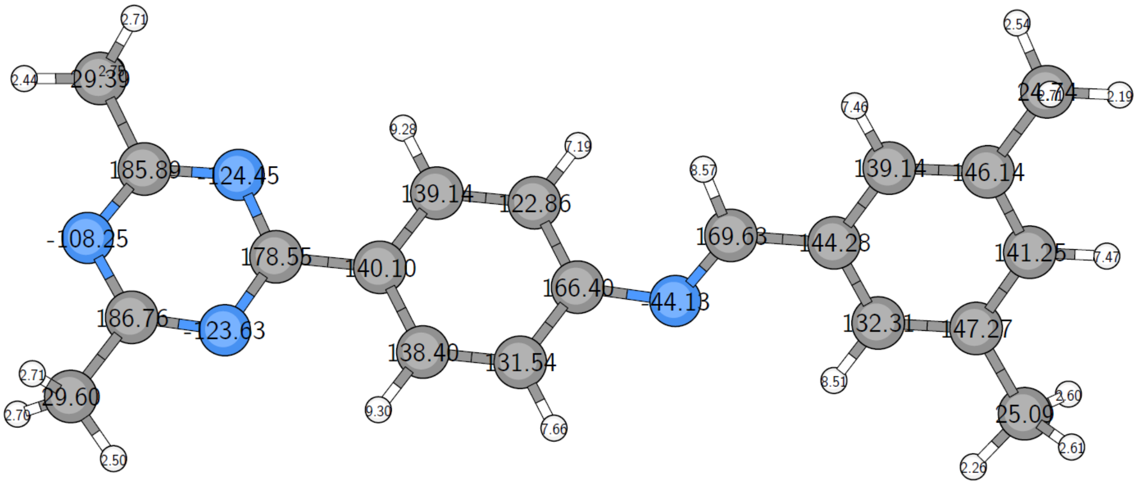

Figure S109: Calculated NMR Chemical Shifts for the PI-3 M model system, obtained on B97-2/pcS-2//PBE0-D3/def2-TZVP level of theory.

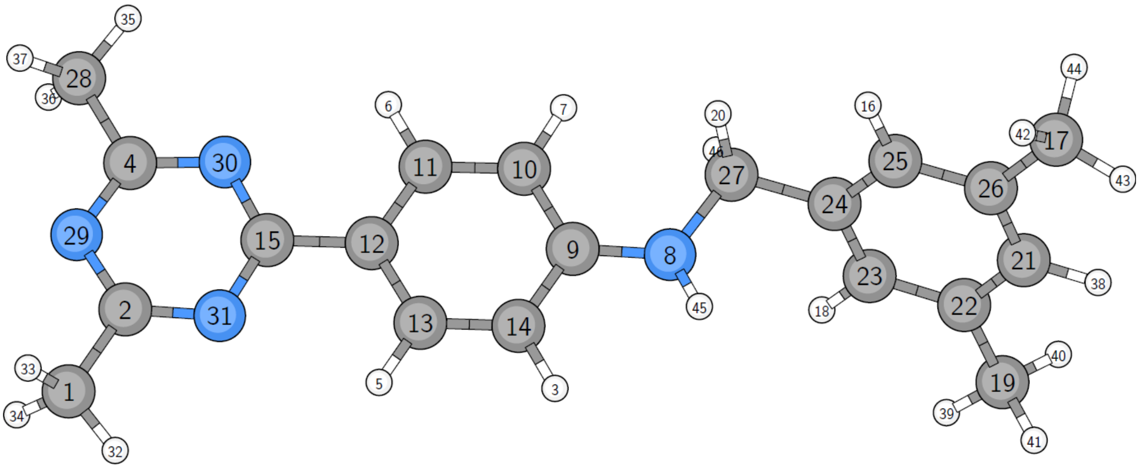

Figure S110: Atom labels for the rPI-3 M model.

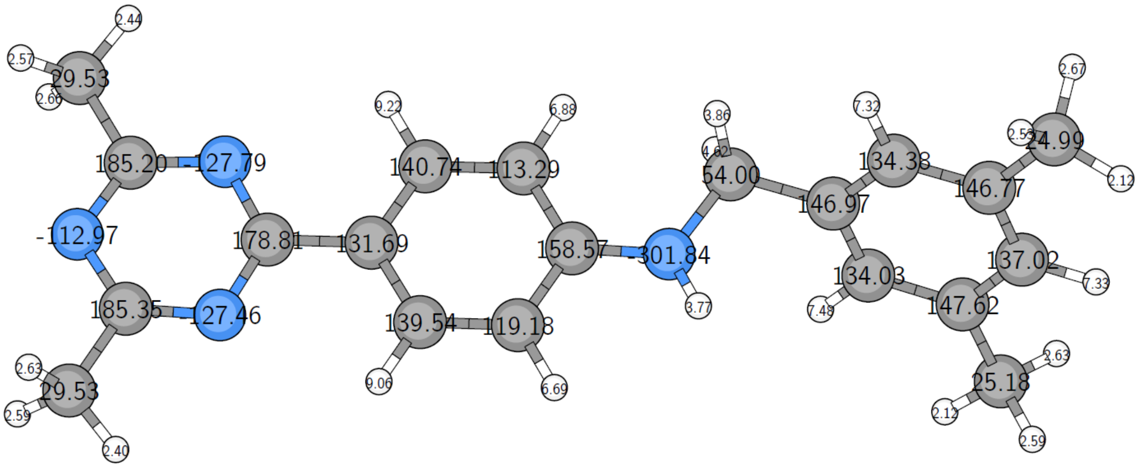

Figure S111: Calculated NMR Chemical Shifts for the rPI-3 M model system, obtained on B97-2/pcS-2//PBE0-D3/def2-TZVP level of theory.

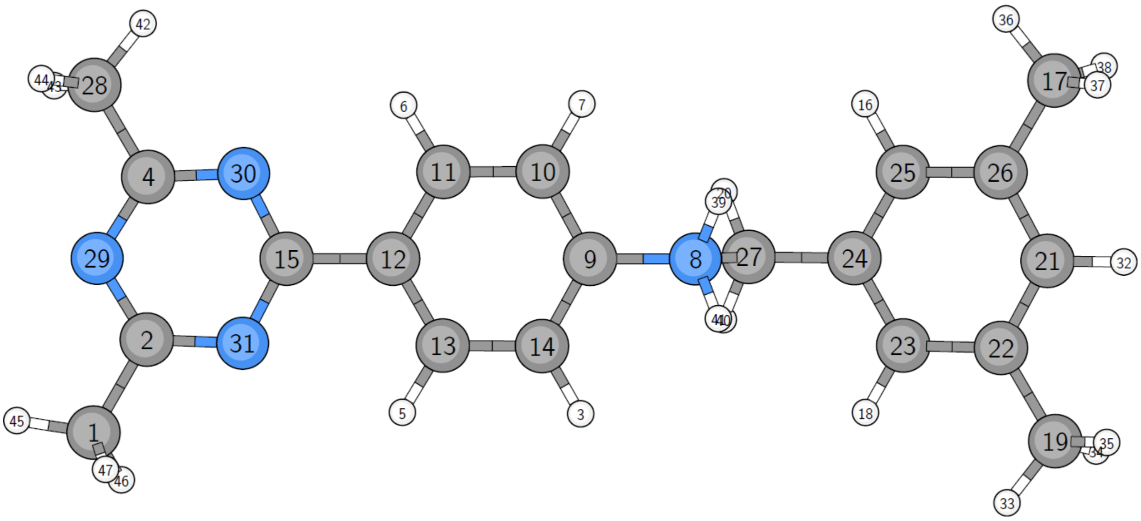

Figure S112: Atom labels for the H+rPI-3 M model.

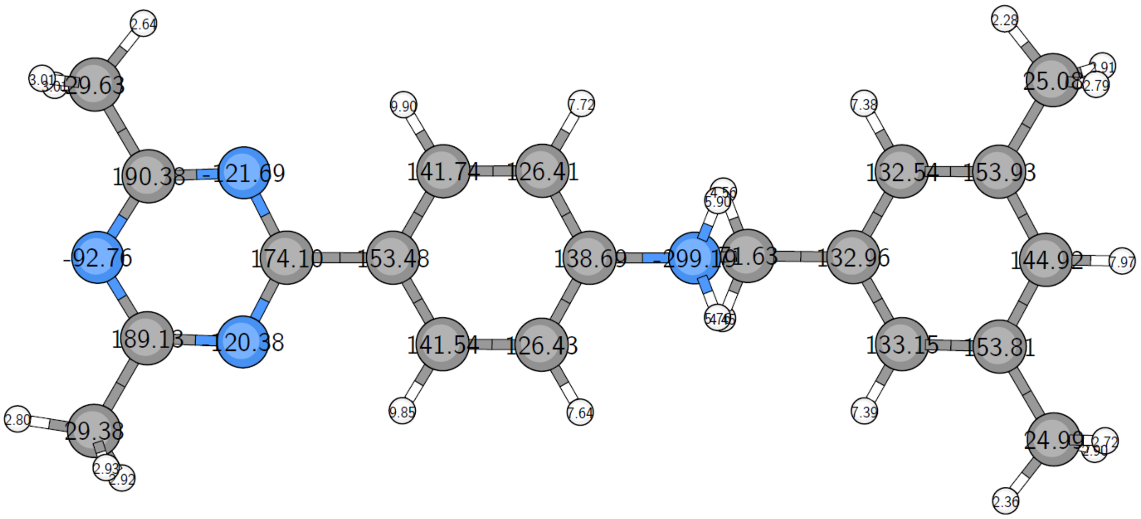

Figure S113: Calculated NMR Chemical Shifts for the H+rPI-3 M model system, obtained on B97-2/pcS-2//PBE0-D3/def2-TZVP level of theory.

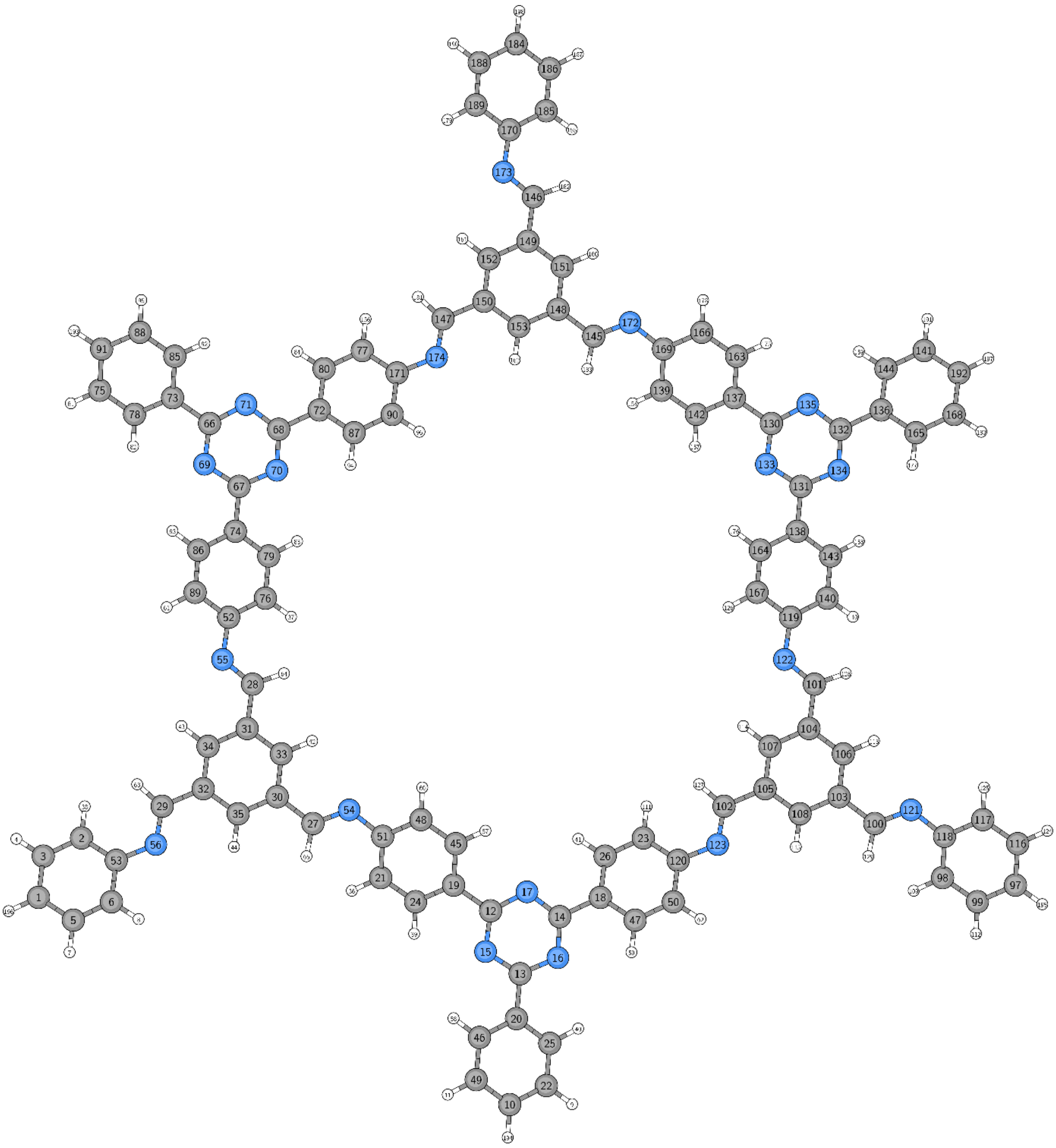

Figure S114: Atom labels for the PI-3 SP model.

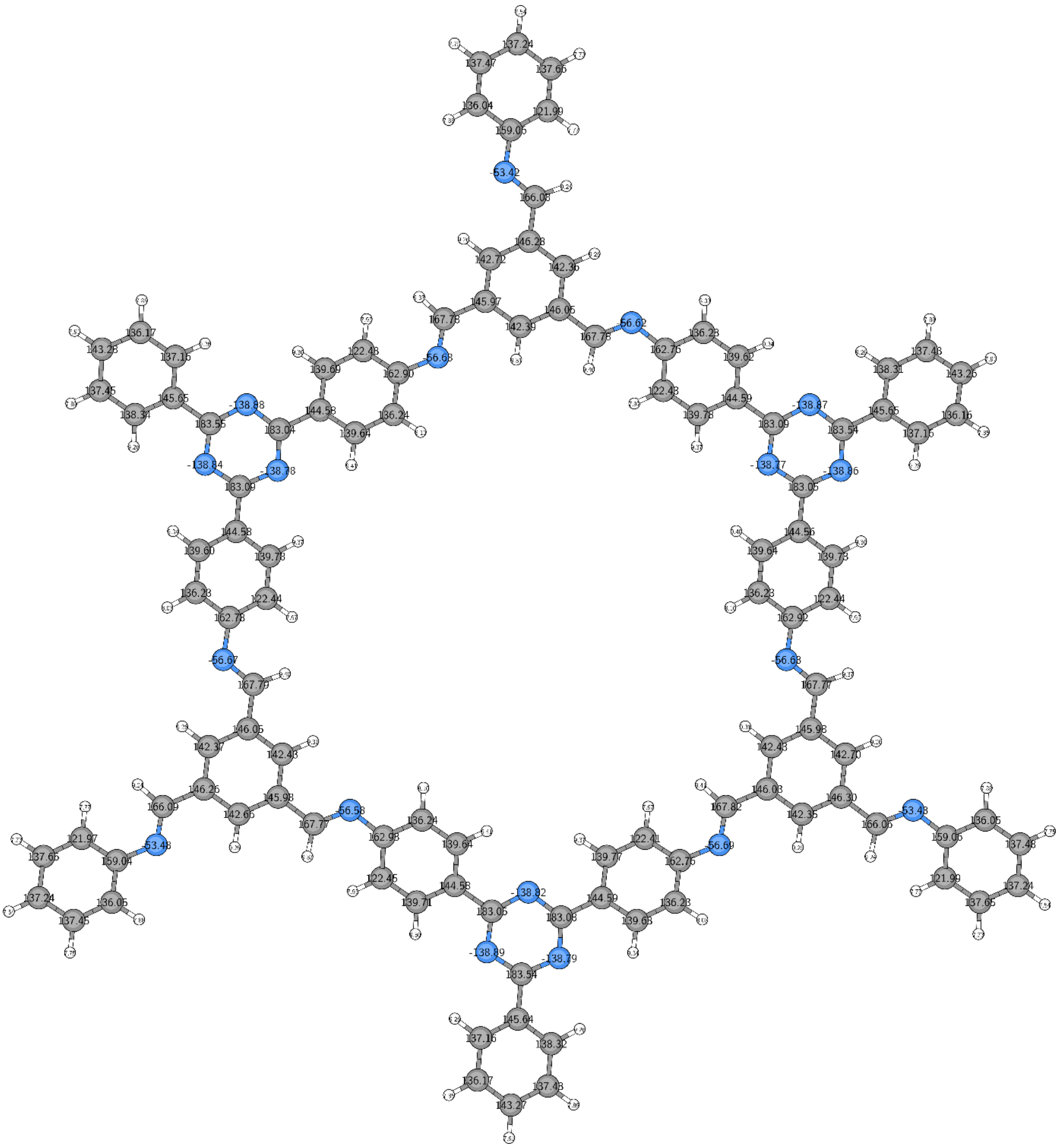

Figure S115: Calculated NMR Chemical Shifts for the PI-3 SP model system, obtained on B97-2/pcS-2//PBE0-D3/def2-TZVP level of theory.

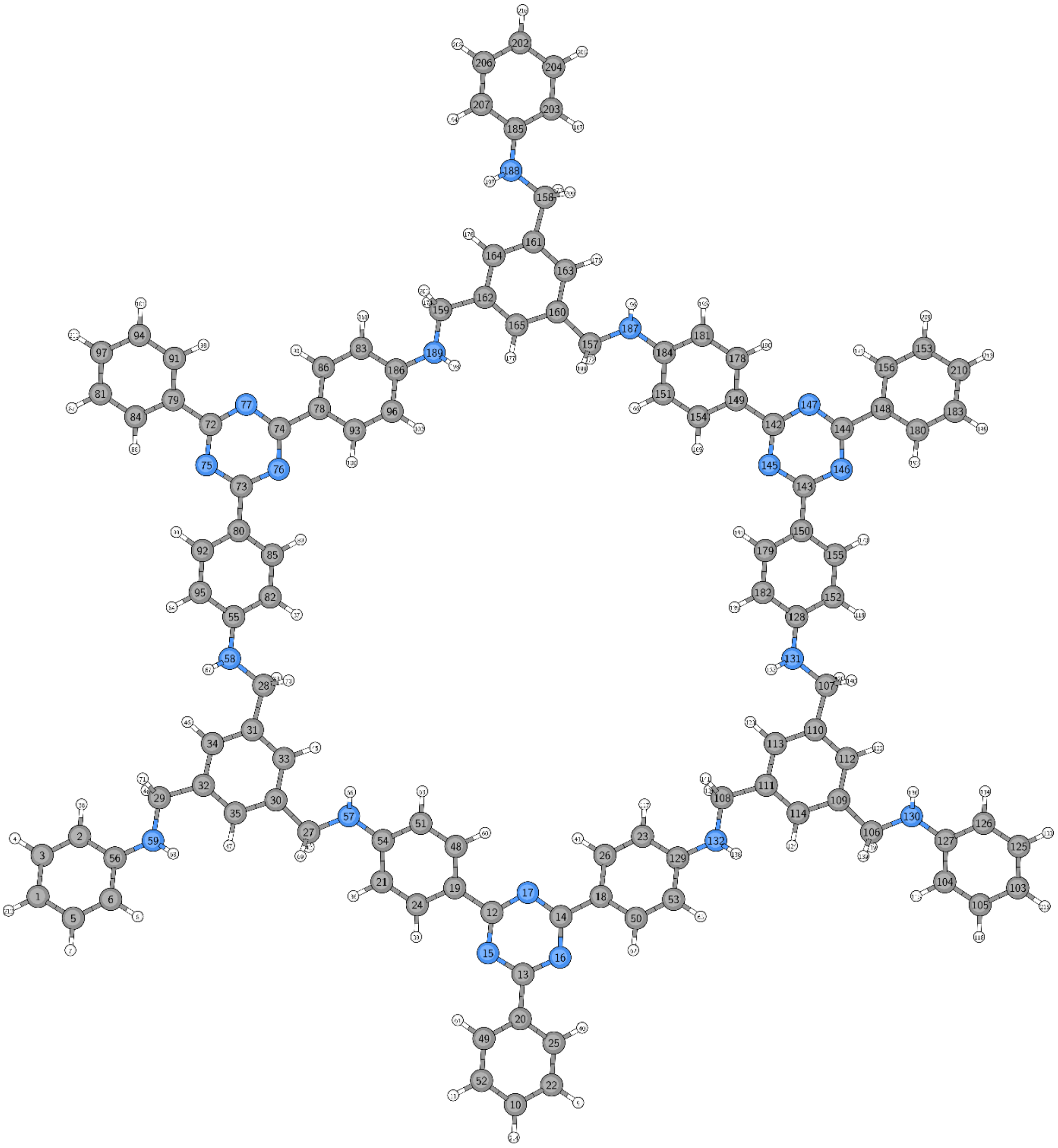

Figure S116: Atom labels for the rPI-3 SP model.

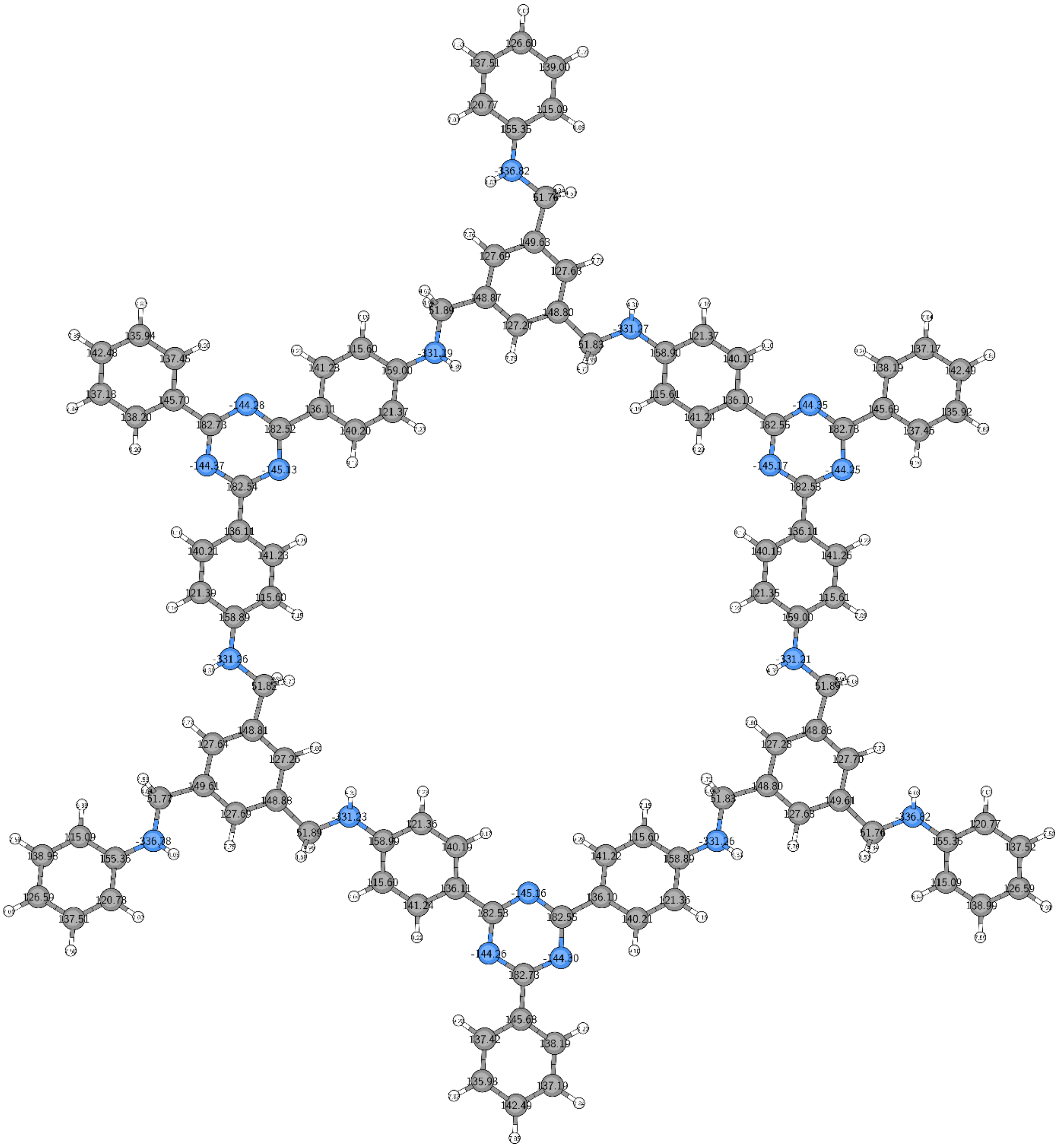

Figure S117: Calculated NMR Chemical Shifts for the rPI-3 SP model system, obtained on PBE0-D3/def2-TZVP level of theory.

Table S5: Calculated imine/amine ^{15}N NMR chemical shifts [ppm] obtained from optimized geometries for the presented model systems. Note that an overestimation of ring strain in the single pore calculation can cause deviations of the calculated NMR shifts, as these are influenced by the conformation of the dihedral angle U (Table S6).

Model	Level of Theory	Atom Number	NMR Shift	Delta
PI-3 M	PBE0-D3/def2-TZVP// B97-2/pcS-2	8	-44.13	
PI-3 M	RI-PBE-D3/def2-TZVP// B97-2/pcS-2	8	-54.63	-10.50
PI-3 SP	RI-PBE-D3/def2-TZVP// B97-2/pcS-2	172	-56.62	
		122	-56.63	
		123	-56.69	
		54	-56.58	
		55	-56.67	
		174	-56.68	
		Average =	-56.65	-2.01
rPI-3 M	PBE0-D3/def2-TZVP// B97-2/pcS-2	8	-301.84	
rPI-3 M	RI-PBE-D3/def2-TZVP// B97-2/pcS-2	8	-314.69	
rPI-3 SP	RI-PBE-D3/def2-TZVP// B97-2/pcS-2	187	-331.27	
		131	-331.21	
		132	-331.26	
		57	-331.23	
		58	-331.26	
		189	-331.19	
		Average =	-331.24	-16.54
H+rPI-3 M	PBE0-D3/def2-TZVP// B97-2/pcS-2	8	-299.19	
H+rPI-3 M	RI-PBE-D3/def2-TZVP// B97-2/pcS-2	8	-312.52	-13.33

Table S6: Calculated ^{15}N NMR chemical shifts [ppm] and dihedral angles U [°], obtained from optimized geometries for the presented model systems.

Model	Level of Theory	NMR Shift	Delta	Dihedral Angle	Delta
PI-3 M	PBE0-D3/def2-TZVP// B97-2/pcS-2	-44.13		43.59	
PI-3 M	RI-PBE-D3/def2-TZVP// B97-2/pcS-2	-54.63	-10.50	43.29	-0.30
PI-3 SP	RI-PBE-D3/def2-TZVP// B97-2/pcS-2	-56.65	-2.01	27.40	-15.89
rPI-3 M	PBE0-D3/def2-TZVP// B97-2/pcS-2	-301.84		10.18	
rPI-3 M	RI-PBE-D3/def2-TZVP// B97-2/pcS-2	-314.69	-12.85	8.97	-1.21
rPI-3 SP	RI-PBE-D3/def2-TZVP// B97-2/pcS-2	-331.24	-16.54	6.46	-2.51

4 Pair Distribution Function (PDF) Analysis

4.1 Methods

4.1.1 Data processing

Analysis of the PDF provides a useful method for interpreting structure information from total scattering data. It provides a sensitive probe of the local structure in amorphous and nanostructured materials, because it does not require symmetry, and treats both Bragg and diffuse scattering equally[31]. A diffraction measurement over a wide range of momentum transfer and with good statistics is required to obtain suitable PDFs for structure analysis. Starting with a 2D data collection of diffracted intensities, azimuthal integration results in the 1D, isotropic powder pattern $I(Q)$, which includes all coherent scattering interferences from the atoms in the sample, defined by the Debye equation,[32] as

$$I_c(Q) = \sum_i \sum_j f(Q)_i f(Q)_j \frac{sin(Qr_{ij})}{Qr_{ij}}. \qquad (1)$$

In order to obtain the pair distribution function, by the formalism of Egami and Billinge[31], the measured powder diffraction intensities are first normalized by the average form factor squared to obtain the total scattering structure function $S(Q)$, defined as

$$S(Q) = \frac{I_c(Q) - \langle f(Q)^2 \rangle + \langle f(Q) \rangle^2}{\langle f(Q) \rangle^2}. \qquad (2)$$

The experimental PDF, denoted $G(r)$, is the truncated Fourier transform of the reduced, total scattering structure function, $F(Q) = Q[S(Q) - 1]$, as

$$G(r) = \frac{2}{\pi} \int_{Q_{min}}^{Q_{max}} F(Q) sin(Qr) dQ, \qquad (3)$$

where $G(r)$ is the magnitude of the scattering momentum transfer for elastic scattering,

$$Q = 4\pi sin\theta / \lambda, \qquad (4)$$

where λ is the probe wavelength and 2θ is the scattering angle. In practice, values of Q_{min} and Q_{max} are determined by the experimental setup, and Q_{max} is often reduced below the experimental maximum to reduce the effects of low signal-to-noise in the high-Q region on the Fourier transformation. To aid in qualitative assessment of the long-distance structural correlations, a modification function can be applied to $F(Q)$ prior to Fourier transformation by,

$$G(r) = \frac{2}{\pi}\int_{Q_{min}}^{Q_{max}} M(Q)F(Q)sin(Qr)dQ \tag{5}$$

and,

$$M(Q) = \frac{sin(Qr_{ij})}{Qr_{ij}}. \tag{6}$$

$M(Q)$ is called a modification function, in this case a Lorch function[33, 34], which damps the intensity of $F(Q)$ to 0 at Q_{max}. This reduces the effects of termination from high- Q signal and noise intensities in the reduced structure function, which suppresses non-structural high frequency oscillations in the PDF.

4.1.2 Real-space structure refinement

The PDF gives the scaled probability of finding two atoms in a material a distance r apart and is relative to the average density of atom pairs in the material. For a macroscopic scatterer, $G(r)$ is calculated from a known structure model according to

$$G(r) = 4\pi r[\rho(r) - \rho_0], \tag{7}$$

$$\rho(r) = \frac{1}{4\pi r^2 N}\sum_i \sum_{j\neq i} \frac{f_i f_j}{\langle f\rangle^2}\delta(r - r_{ij}). \tag{8}$$

Here, ρ_0 is the average number density of the material and ϱ(r) is the local atomic pair density, which is the mean weighted density of neighbor atoms at distance r from an atom at the origin. The sums in $\rho(r)$ run over all atoms in the sample, f_i is the scattering factor of atom i, $\langle f\rangle$ is the average scattering factor and r_{ij} is the distance between atoms i and j. In this study, Eqs. (4) and (5) were used to fit the PDF generated from a structure model to the experimental PDFs in using the program PDFgui.[5] The delta functions in Eq. (5) were broadened to account for atom displacements and a finite Q-range in the experiment, and the equation was modified to account for signal damping and broadening due to experimental effects. PDF modelling was performed by adjusting the lattice parameters *a*=*b* and *c*, atomic displacement parameters (ADPs) $U_{11} = U_{22} \neq U_{33}$, correlated motion of neighbouring atoms δ2, domain size (*spdiameter*), and a global scale factor. The refinements were run by minimizing R_w, calculated as

$$R_w = \sqrt{\frac{\sum_{i=1}^{n}[G_{obs}(r_i) - G_{calc}(r_i, P)]^2}{\sum_{i=1}^{n} G_{obs}(r_i)^2}}, \tag{9}$$

Where P is the set of refined parameters, which was used to quantify the goodness-of-fit for the model. The model was constructed in each case, for the amine and imine structures, with 2D layers which had been relaxed by quantum chemical calculations (see Section 3.1).The

relaxed layer was then placed into a starting cell based on the repeat distances in the relaxed layer, and the approximate stacking distance from XRPD analysis. Additional supercells were constructed with 2, 4, 8 and 16 layers to test the effects of random interlayer translational disorder.

4.2 Results

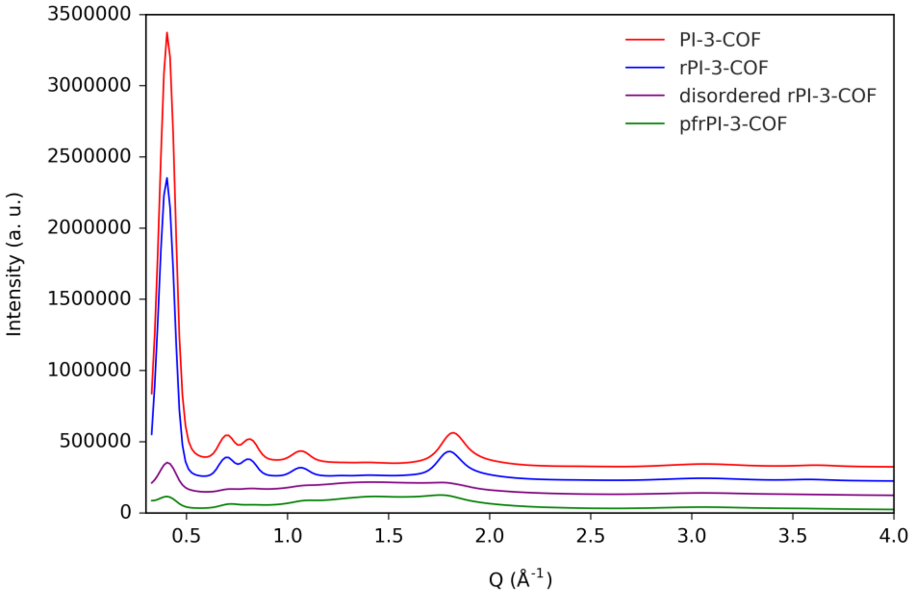

Figure S118: The XRPD patterns for all samples are plotted in intensity versus *Q*. The patterns were rescaled by normalization of the diffuse, high-*Q* intensities and are offset for clarity. The ordered COFs, PI-3-COF and rPI-3-COF, show sharp Bragg diffraction peaks indexed by $P\bar{6}$ symmetry to 100, 2-10, 200, 3-20, and primarily 001 from low to high, respectively. The disordered COFs, disordered rPI-3-COF and pfrPI-3-COF, also show features for the same peak indexing, though significantly reduced in intensity, indicating that at least some content maintains intralayer connectivity and an ordered stacking arrangement.

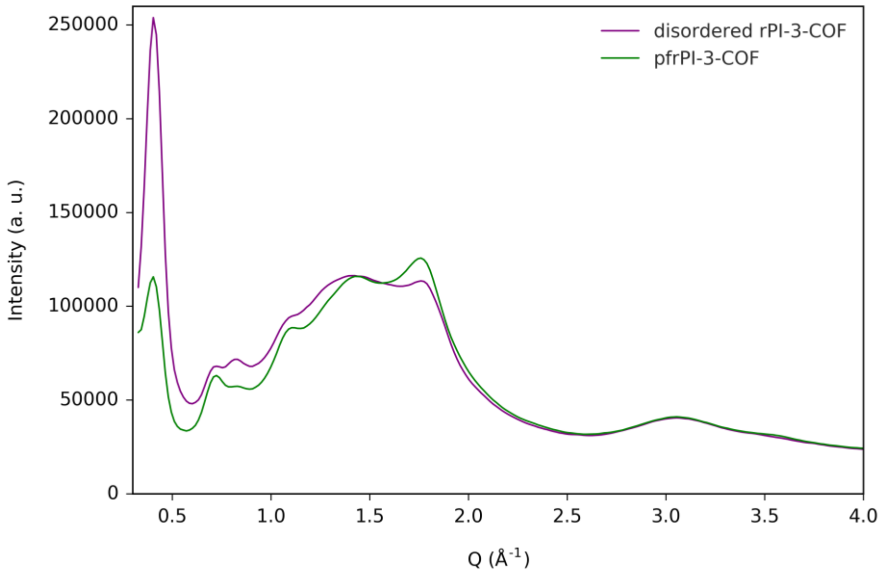

Figure S119: The XRPD patterns for disordered samples are overlayed in intensity versus *Q*. The patterns were rescaled by normalization of the diffuse, high-*Q* intensities. The 100 and 200 peaks are substantially more prominent in the disordered rPI-3-COF, while the 001 peak is more prominent in pfrPI-3-COF, relative to the 100 peak. This indicates differences in the disordered sample structures, possibly larger lateral intralayer coherence in the in disordered rPI-3-COF, but more layers contributing to ordered stacking domains in pfrPI-3-COF. Formyl groups in pfrPI-3-COF may also play a role in decreasing the planarity of the layers, which could decrease in-plane coherence, i.e. 100 peak. However, differences in relative intensities may also be due to the presence of formyl groups sticking into the pores in pfrPI-3-COF, which reduces the scattering contrast between pore and framework.

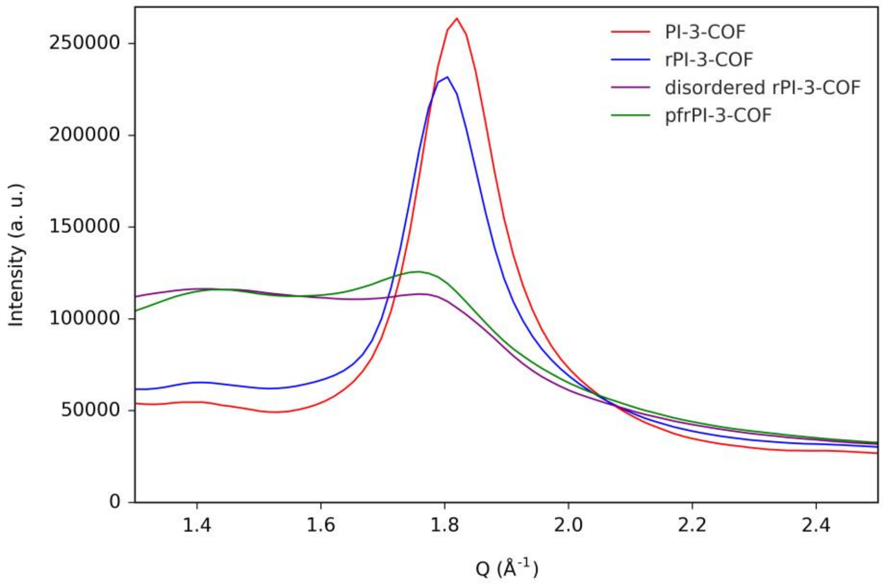

Figure S120: The XRPD patterns for all samples are overlayed in intensity versus *Q*, focused on the 001 peaks. The patterns were rescaled by normalization of the diffuse, high-*Q* intensities. It is clear that at least some content in every sample exists in a state of ordered interlayer stacking along the [001] direction. However, the stacking distances are not equivalent. Pseudo-Voigt functions with a linear baseline were fit to the peaks to determine their positions. PI-3-COF (red) shows the shortest interlayer distance at 3.4528(9) Å which is likely due to stronger bonding and more planar conformation of the imine bonding leading to a more ordered framework structure, therefore allowing closer stacking. rPI-3-COF (blue) stacking distance is slightly longer at 3.485(1) Å, which is likely due to increased disorder of the framework

due to larger torsions of the amine bonding. The next largest stacking distance is disordered rPI-3-COF (purple) at 3.515(3) Å, and the largest, pfrPI-3-COF (green) 3.547(2) Å. The larger stacking distances in the disordered forms makes sense due to reduced stacking coherence and likely increased disorder in the amine torsion angles. Furthermore, interactions of the formyl groups in pfrPI-3-COF may also play a role in increasing the stacking distance between layers. A similar peak fitting analysis was performed on the 100 peak. In this case, the positions resulted in *d*-spacings of 15.50(1) (PI-3-COF), 15.66(1) (rPI-3-COF), 15.415(7) (disordered rPI-3-COF), 15.361(8) (pfrPI-3-COF). This could suggest, for the segments of all samples which are coherently ordered in the *ab* plane, that the imine to amine conversion results in a larger pore-to-pore spacing due to the expanded N-C bond lengths, but that the disordering process leads to a contracted pore-to-pore spacing, which may have to do with larger and more random out-of-plane torsions due to larger neighbor-layer offsets.

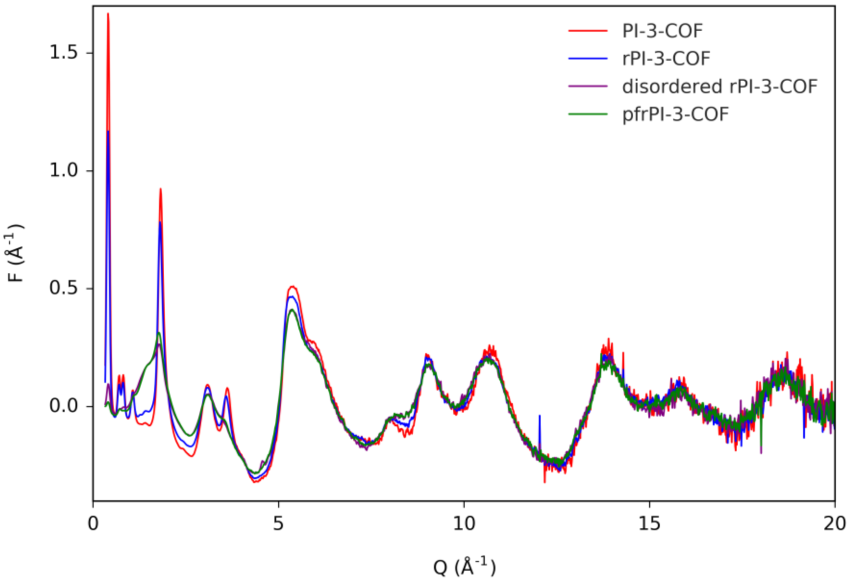

Figure S121: The reduced total scattering patterns for all samples are overlayed. Distinct structural information, not clearly visible in the raw diffraction patterns, is evident out to high *Q* values, which is made apparent by the normalization procedure. High similarity in the patterns from approximately 5-20 $Å^{-1}$ is a good indicator of similarity in local molecular motifs present in the structure, e.g. imine versus amine bonded layers. For example a shift to lower *Q* (longer *d*-spacing) of the feature at 10-12 $Å^{-1}$ is present in all amine-based samples.

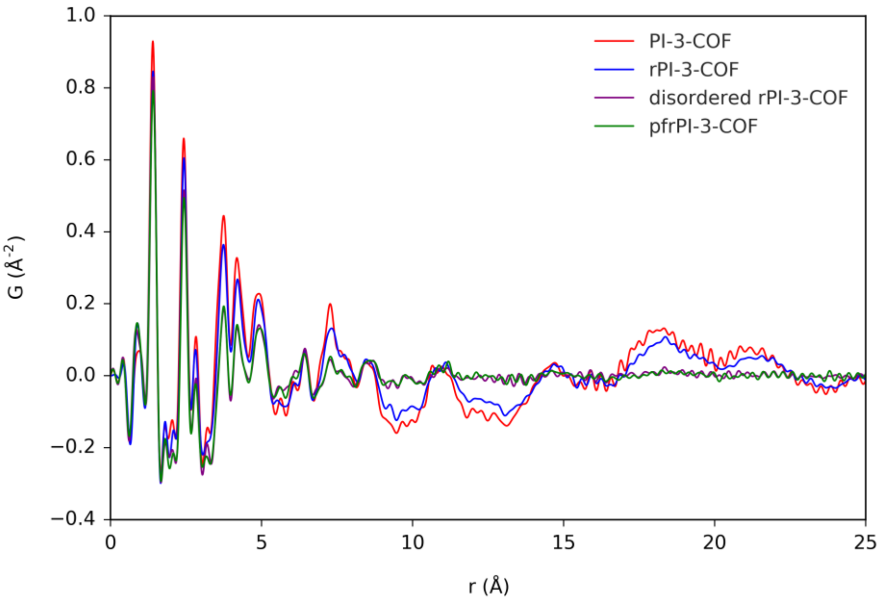

Figure S122: The PDFs, $G(r)$, for all samples are overlayed. All samples show high similarity in the peak positions and relative amplitudes up to approximately 7 Å, which is further evidence that that layer connectivity is still intact in the disordered state. Beyond this, there is some similarity in positions but drastic differences in the relative amplitude between ordered and disordered sample structures. There are distinct medium- and long-range order structuring in PI-3-COF and rPI-3-COF, which consists of two primary oscillations, one with shorter wavelength due to the ordering of the stacked layers, and one with longer wavelength due to the ordering of the porous channels. There is an apparent loss of structural coherence beyond approximately 12 Å for the disordered samples, indicating that the spatial relationships of the stacked layers, and of the porous channels, are significantly reduced.

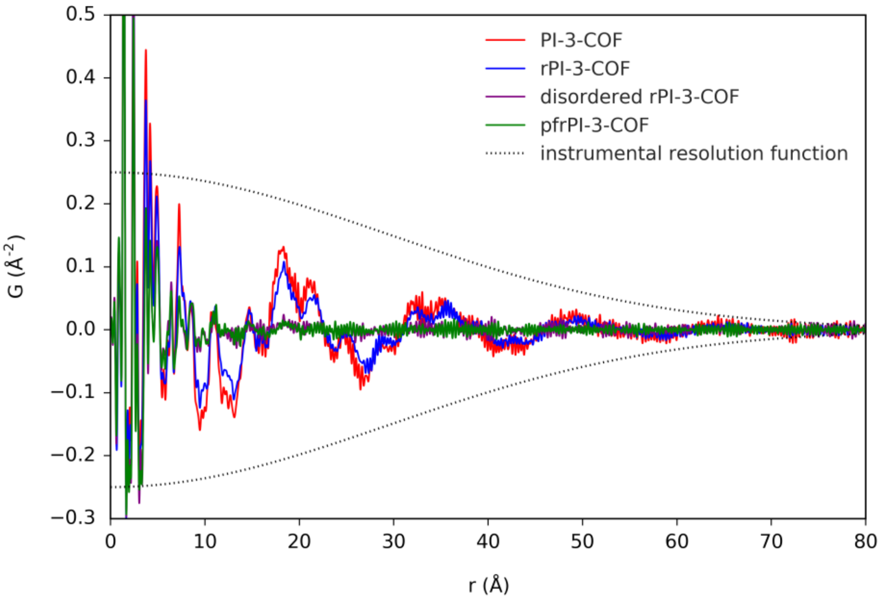

Figure S123: The PDFs, $G(r)$, for all samples are overlayed and plotted over a longer distance range. Here, density modulations due to stacked layers and porous channels can be observed for the crystalline samples out to much higher distances, whereas the disordered samples are flat, indicating no long range order. The long range signals are damped due to the limited reciprocal space resolution of the measurement.

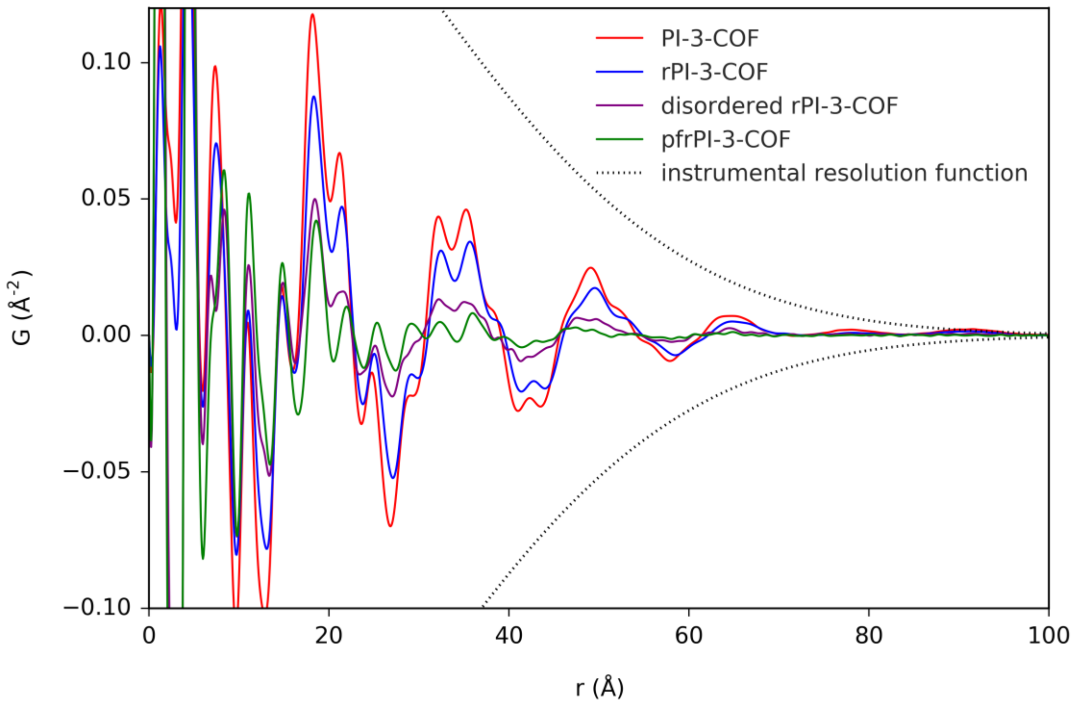

Figure S124: The PDFs, $G(r)$, for all samples are again overlayed and plotted over a longer distance range. However, the data have been reprocessed using a Qmax of 5.0 $Å^{-1}$ and a Lorch function as described in the methods section to remove effects of noise and termination on the resulting PDF data. The plot is also magnified close into the low amplitude signals at higher distances. Here, we can see the structural signals from the 100 and 001 Bragg peaks, previously noted in the diffraction patterns of the disordered samples. First, an oscillation distinctly coming from stacked layers is present for pfrPI-3-COF (green) up to at least 50-60 Å (14-17 layers). It is also present in disordered rPI-3-COF (purple), but broadened and with reduced amplitude. As with the diffraction data, the roles are reversed for ordering of the porous channels, distinct oscillations between pore and framework in disordered rPI-3-COF to at least 60 Å (4-5 channels).

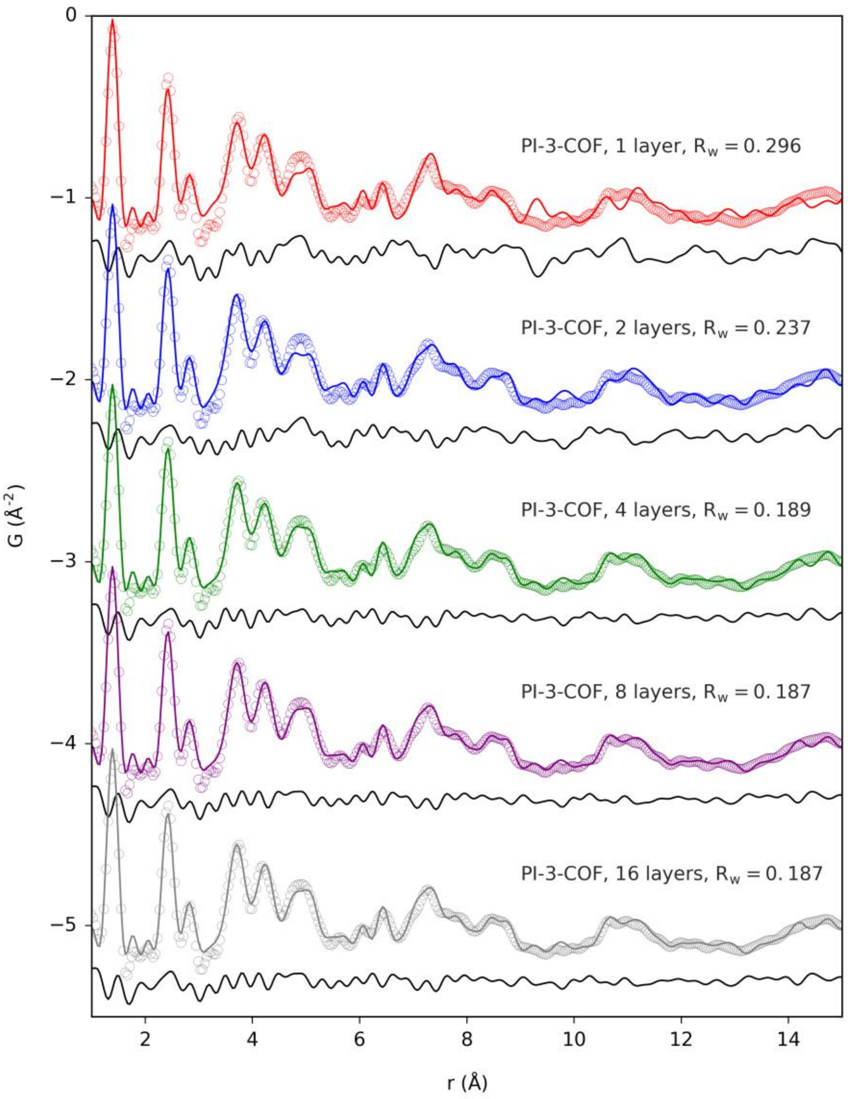

Figure S125: Structure refinement (solid line) to the PDF from PI-3-COF (dots) for models with 1, 2, 4, 8, and 16 layers. The layers allowed to randomly translate forward or backward along the 1-10 direction. Difference curves are shown offset below the fit (black).

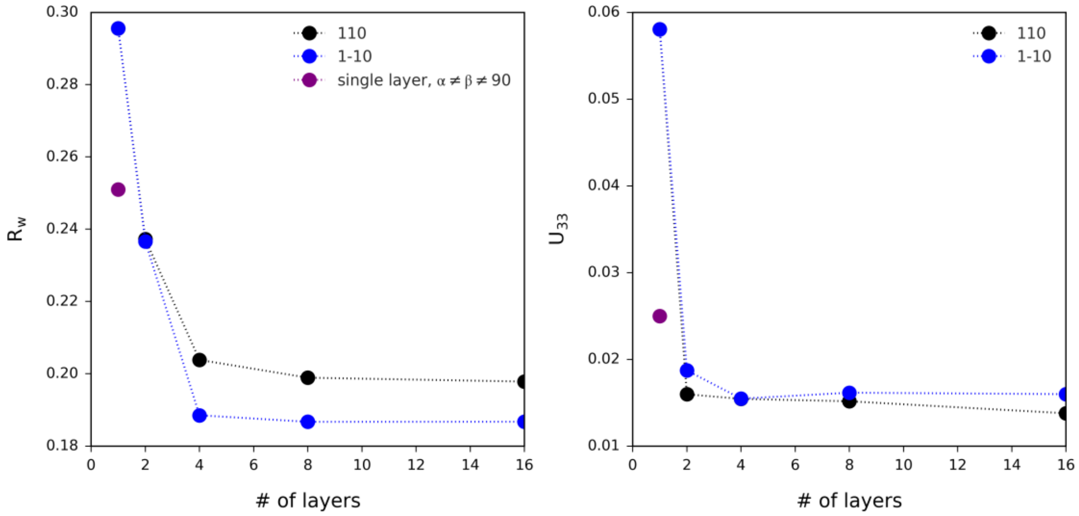

Figure S126: R_w (left) and U_{33} (right) values resulting from fitting of structural models with increasing numbers of layers allowed to translate forward and backward along either 110 or 1-10 directions to the PDF data from PI-3-COF. The R_w value decreases dramatically on adding a single or few layers, indicating that the structuring prefers a staggered relationship between neighboring layers rather than perfectly eclipsed stacking as indicated by the average structure. Furthermore, disorder in the stacking vectors can also account for the very large interlayer ADP (U_{33}) observed when modelling with only a single layer, which is artificially enlarged to reduce the contribution from non-preferred, eclipsed interlayer atom-pair distances. The data appears to prefer translation along the 1-10 direction as opposed to 110, although this does not account for the possibility of combining random displacements along the other two equivalent directions, which could be equally likely. In addition, the model with a single layer was also refined allowing the α and β angles to assume non-90° angles which also significantly increased the goodness-of-fit, supporting the preference for locally slipped stacking of the neighboring layers.

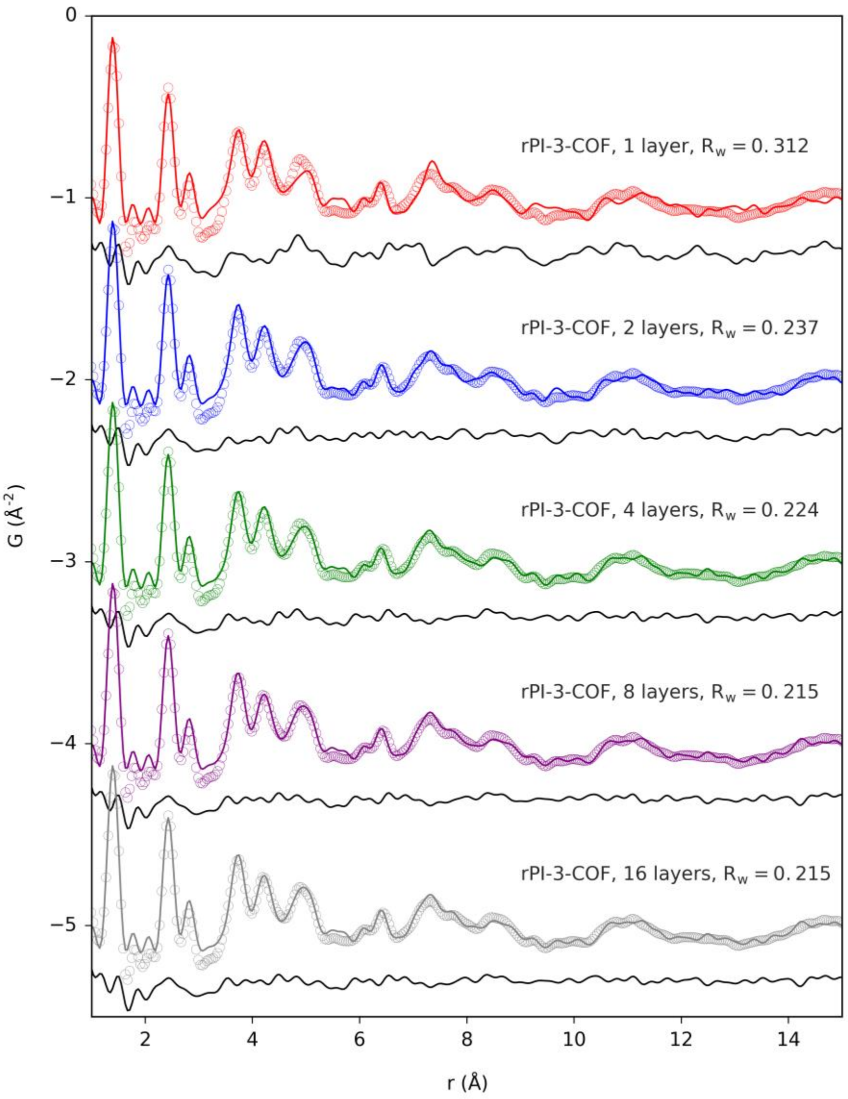

Figure S127: Structure refinement (solid line) to the PDF from rPI-3-COF (dots) for models with 1, 2, 4, 8, and 16 layers. The layers allowed to randomly translate forward or backward along the 1-10 direction. Difference curves are shown offset below the fit (black).

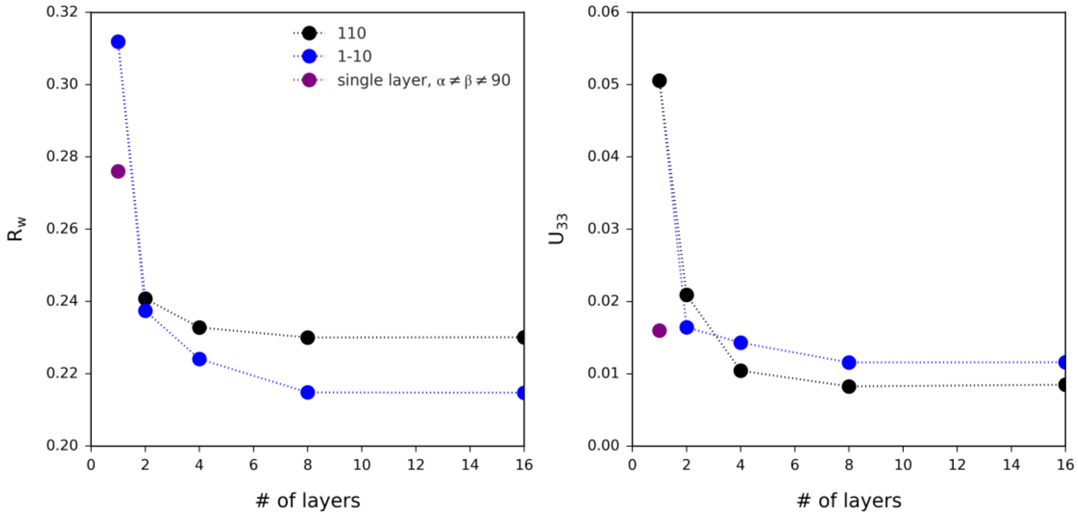

Figure S128: R_w (left) and U_{33} (right) values resulting from fitting of structural models with increasing numbers of layers allowed to translate forward and backward along either 110 or 1-10 directions to the PDF data from rPI-3-COF. The R_w value decreases dramatically on adding a single or few layers, indicating that the structuring prefers a staggered relationship between neighboring layers rather than perfectly eclipsed stacking as indicated by the average structure. Furthermore, disorder in the stacking vectors can also account for the very large interlayer ADP (U_{33}) observed when modeling with only a single layer, which is artificially enlarged to reduce the contribution from non-preferred, eclipsed interlayer atom-pair distances. The data appears to prefer translation along the 1-10 direction as opposed to 110, although this does not account for the possibility of combining random displacements along the other two equivalent directions, which could be equally likely. In addition, the model with a single layer was also refined allowing the α and β angles to assume non-90° angles which also significantly increased the goodness-of-fit, supporting the preference for locally slipped stacking of the neighboring layers.

We consider the stacking vector to be the translation of one layer along the *ab* plane from an eclipsed position with respect to the neighboring layer in the hexagonal cell, α=β=90°. For the 1-10 direction, the shift can be calculated by $2*f*a*\cos(30°)$ where f is the fractional shift value, and then relative shifts can be obtained from the difference between subsequent neighbor shifts. Thus the average shifts obtained from the 16 layer models are 1.48(58) and 1.88(99) Å for the imine and amine PDF refinements, respectively, which match well within the ranges suggested by Spitler et al. 1.7-1.8 Å[35] and Koo, Dichtel & Clancy i.e. 1.5-2.8 Å[36], giving direct experimental evidence for random local shifting of neighboring layers despite the fact that the average crystallographic structure appears eclipsed.

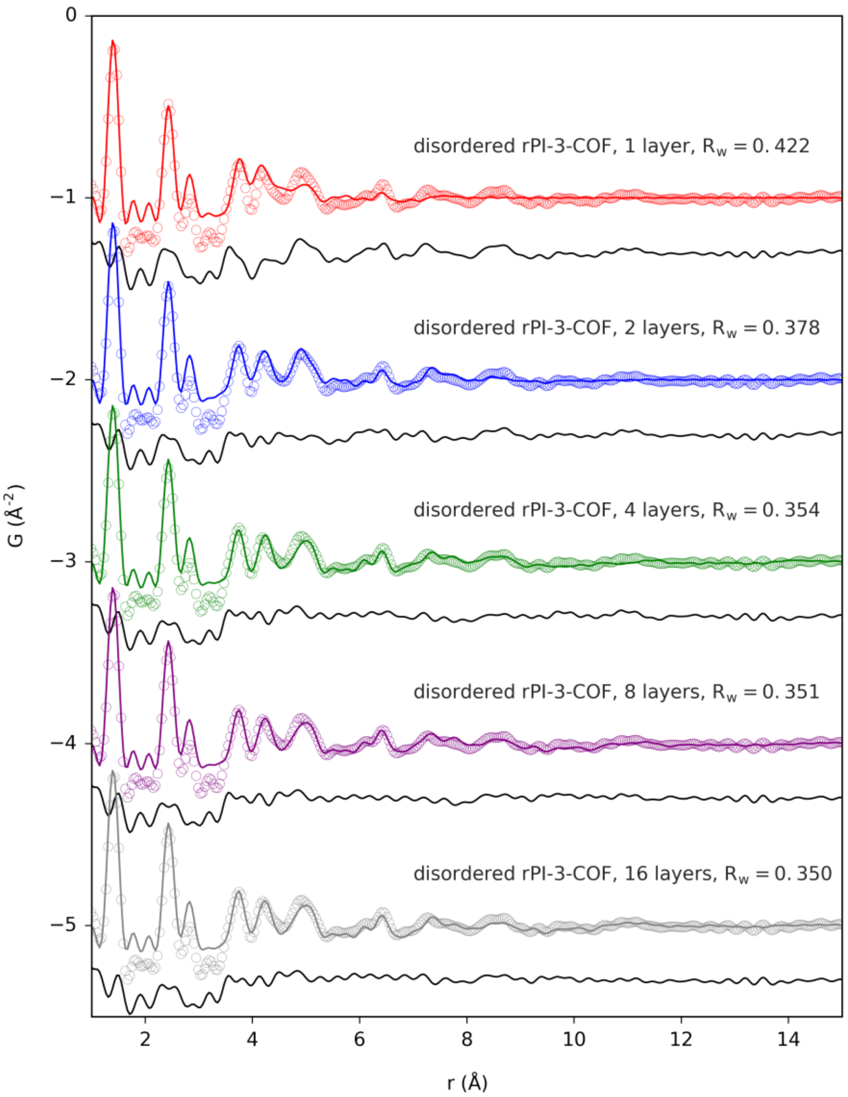

Figure S129: Structure refinement (solid line) to the PDF from disordered rPI-3-COF (dots) for models with 1, 2, 4, 8, and 16 layers. The layers allowed to freely translate in the *ab* plane. Difference curves are shown offset below the fit (black).

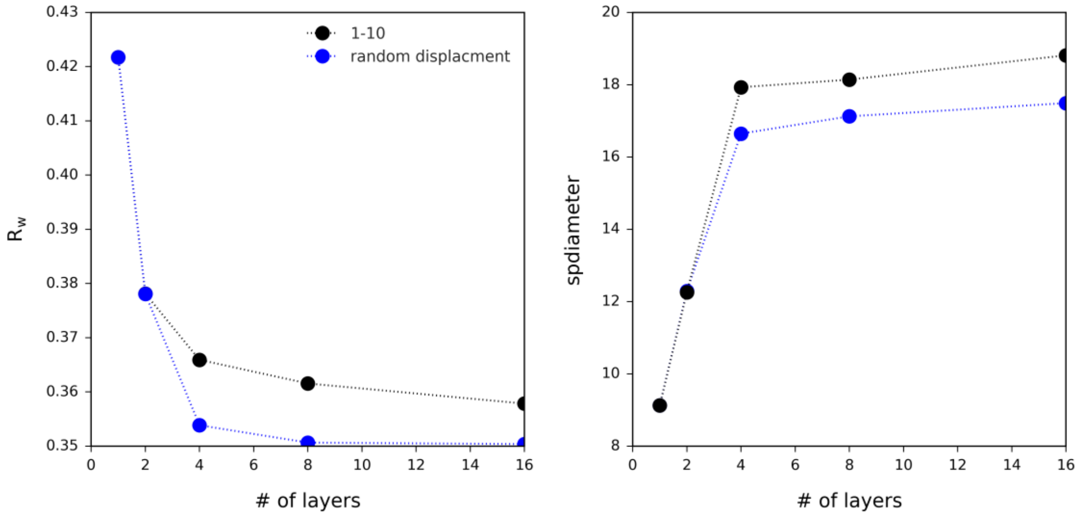

Figure S130: R_w (left) and the coherence length (spdiameter from PDFgui program) (right) values resulting from fitting of structural models with increasing numbers of layers allowed to translate forward and backward along the 1-10 direction as in the previous model, and freely in the *ab* plane, to the PDF data from disordered rPI-3-COF. This refinement prefers larger magnitude, and random direction, translations to reduce the contribution of coherent intermolecular atom-pairs to the simulated PDF. As the intermolecular signal is reduced by random translations, the dampening of the simulated signal by spdiameter is reduced, eventually returning ~17-19 Å, which is approximately the in-plane coherence we might expect given the distances in which sharp features exist in the measured PDF.

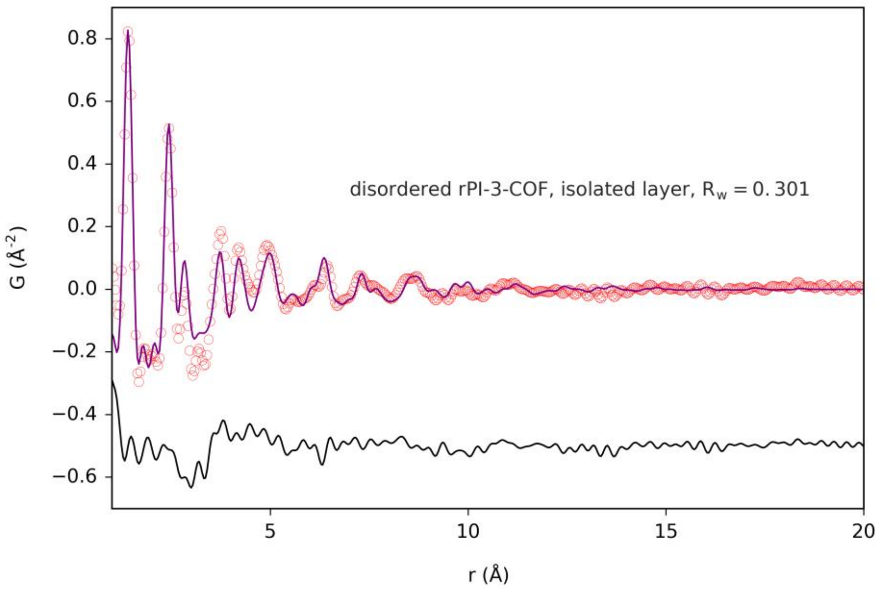

Figure S131: An additional refinement of a single, isolated layer of the amine structure was attempted to completely eliminate all intermolecular structural correlations from the simulated PDF. This gives the best fit, further supporting that all sharp features in the disordered sample PDFs come from intramolecular correlations from a connected layer, at least up to ~20 Å.

References

1. Chupas P. J. et al. Rapid-acquisition pair distribution function (RA-PDF) analysis. *J. Appl. Crystallogr.* **2003**, *36*, 1342-1347.

2. Hammersley A. P. et al. Two-dimensional detector software: From real detector to idealised image or two-theta scan. *High Press. Res.* **1996**, *14*, 235-248.

3. Juhás P., Davis T., Farrow C. L., Billinge S. J. L. PDFgetX3: a rapid and highly automatable program for processing powder diffraction data into total scattering pair distribution functions. *J. Appl. Crystallogr.* **2013**, *46*, 560-566.

4. Yang X., Juhas P., Farrow C. L., Billinge S. J. xPDFsuite: an end-to-end software solution for high throughput pair distribution function transformation, visualization and analysis. **2015**. Preprint at https://arxiv.org/abs/1402.3163.

5. Farrow C. L. et al. PDFfit2 and PDFgui: computer programs for studying nanostructure in crystals. *J. Phys.: Condens. Matter* **2007**, *19*, 335219.

6. Perdew J. P., Burke K., Ernzerhof M. Generalized Gradient Approximation Made Simple. *Phys. Rev. Lett.* **1996**, *77*, 3865-3868.

7. Grimme S., Antony J., Ehrlich S., Krieg H. A consistent and accurate ab initio parametrization of density functional dispersion correction (DFT-D) for the 94 elements H-Pu. *J. Chem. Phys.* **2010**, *132*, 154104.

8. Schäfer A., Huber C., Ahlrichs R. Fully optimized contracted Gaussian basis sets of triple zeta valence quality for atoms Li to Kr. *J. Chem. Phys.* **1994**, *100*, 5829-5835.

9. Eichkorn K., Weigend F., Treutler O., Ahlrichs R. Auxiliary basis sets for main row atoms and transition metals and their use to approximate Coulomb potentials. *Theor. Chem. Acc.* **1997**, *97*, 119-124.

10. Burow A. M., Sierka M. Linear Scaling Hierarchical Integration Scheme for the Exchange-Correlation Term in Molecular and Periodic Systems. *J. Chem. Theory Comput.* **2011**, *7*, 3097-3104.

11. Burow A. M., Sierka M., Mohamed F. Resolution of identity approximation for the Coulomb term in molecular and periodic systems. *J. Chem. Phys.* **2009**, *131*, 214101.

12. Grajciar L. Low-memory iterative density fitting. *J. Comput. Chem.* **2015**, *36*, 1521-1535.

13. Lazarski R., Burow A. M., Grajciar L., Sierka M. Density functional theory for molecular and periodic systems using density fitting and continuous fast multipole method: Analytical gradients. *J. Comput. Chem.* **2016**, *37*, 2518-2526.

14. Łazarski R., Burow A. M., Sierka M. Density Functional Theory for Molecular and Periodic Systems Using Density Fitting and Continuous Fast Multipole Methods. *J. Chem. Theory Comput.* **2015**, *11*, 3029-3041.

15. TURBOMOLE V7.3 2018, a development of University of Karlsruhe and Forschungszentrum Karlsruhe GmbH, 1989-2007, TURBOMOLE GmbH, since 2007; available from http://www.turbomole.com.

16. Adamo C., Barone V. Toward reliable density functional methods without adjustable parameters: The PBE0 model. *J. Chem. Phys.* **1999**, *110*, 6158-6170.

17. Ernzerhof M., Scuseria G. E. Assessment of the Perdew–Burke–Ernzerhof exchange-correlation functional. *J. Chem. Phys.* **1999**, *110*, 5029-5036.

18. Wilson P. J., Bradley T. J., Tozer D. J. Hybrid exchange-correlation functional determined from thermochemical data and ab initio potentials. *J. Chem. Phys.* **2001**, *115*, 9233-9242.

19. Jensen F. Basis Set Convergence of Nuclear Magnetic Shielding Constants Calculated by Density Functional Methods. *J. Chem. Theory Comput.* **2008**, *4*, 719-727.

20. Kussmann J., Ochsenfeld C. Preselective Screening for Linear-Scaling Exact Exchange-Gradient Calculations for Graphics Processing Units and General Strong-Scaling Massively Parallel Calculations. *J. Chem. Theory Comput.* **2015**, *11*, 918-922.

21. Kussmann J., Ochsenfeld C. Pre-selective screening for matrix elements in linear-scaling exact exchange calculations. *J. Chem. Phys.* **2013**, *138*, 134114.

22. Gattuso G. et al. Amino Surface-Functionalized Tris(calix[4]arene) Dendrons with Rigid C3-Symmetric Propeller Cores. *Eur. J. Org. Chem.* **2011**, *28*, 5696-5703.

23. Gomes R., Bhaumik A. A new triazine functionalized luminescent covalent organic framework for nitroaromatic sensing and CO2 storage. *RSC Adv.* **2016**, *6*, 28047-28054.

24. Tanaka H., Shizu K., Nakanotani H., Adachi C. Twisted Intramolecular Charge Transfer State for Long-Wavelength Thermally Activated Delayed Fluorescence. *Chem. Mater.* **2013**, *25*, 3766-3771.

25. Li S.-H., Huang H.-P., Yu S.-Y., Li X.-P. Design and Synthesis of Polypyrazolyl Compounds as a New Type of Versatile Building Blocks. *Chin. J. Chem* . **2006**, *24*, 1225-1229.

26. Auras F. et al. Synchronized Offset Stacking: A Concept for Growing Large-Domain and Highly Crystalline 2D Covalent Organic Frameworks. *J. Am. Chem. Soc.* **2016**, *138*, 16703-16710.

27. Bai L. et al. Nanoscale covalent organic frameworks as smart carriers for drug delivery. *Chem. Commun.* **2016**, *52*, 4128-4131.

28. Haase F. et al. Topochemical conversion of an imine- into a thiazole-linked covalent organic framework enabling real structure analysis. *Nat. Commun.* **2018**, *9*, 2600.

29. Haase F. et al. Tuning the stacking behaviour of a 2D covalent organic framework through non-covalent interactions. *Mater. Chem. Front.* **2017**, *1*, 1354-1361.

30. Coelho A. A. TOPAS and TOPAS-Academic: an optimization program integrating computer algebra and crystallographic objects written in C++. *J. Appl. Crystallogr.* **2018**, *51*, 210-218.

31. Egami T., Billinge S. J. L. *Underneath the Bragg peaks: structural analysis of complex materials*; Pergamon: Oxford, 2012.

32. Debye P. Zerstreuung von Röntgenstrahlen. *Ann. Phys.* **1915**, *351*, 809-823.

33. Lorch E. Neutron diffraction by germania, silica and radiation-damaged silica glasses. *J. Phys. C* **1969**, *2*, 229-237.

34. Soper A. K., Barney E. R. On the use of modification functions when Fourier transforming total scattering data. *J. Appl. Crystallogr.* **2012**, *45*, 1314-1317.

35. Spitler E. L. et al. A 2D covalent organic framework with 4.7-nm pores and insight into its interlayer stacking. *J. Am. Chem. Soc.* **2011**, *133*, 19416-19421.

36. Koo B. T., Dichtel W. R., Clancy P. A classification scheme for the stacking of two-dimensional boronate ester-linked covalent organic frameworks. *J. Mater. Chem.* **2012**, *22*, 17460-17469.
